# Duodenal-Jejunal Bypass Liner for the management of Type 2 Diabetes Mellitus and Obesity: A Multicenter Randomized Controlled Trial

**DOI:** 10.1097/SLA.0000000000004980

**Published:** 2021-06-15

**Authors:** Aruchuna Ruban, Alexander D. Miras, Michael A. Glaysher, Anthony P. Goldstone, Christina G. Prechtl, Nicholas Johnson, Navpreet Chhina, Werd Al-Najim, Madhawi Aldhwayan, Natalia Klimowska-Nassar, Claire Smith, Joanne Lord, Jia V. Li, Lilliam Flores, Moaz Al-Lababidi, Georgios K. Dimitriadis, Mayank Patel, Michael Moore, Harvinder Chahal, Ahmed R. Ahmed, Jonathan Cousins, Ghadah Aldubaikhi, Ben Glover, Emanuela Falaschetti, Hutan Ashrafian, Carel W. le Roux, Ara Darzi, James P. Byrne, Julian P. Teare

**Affiliations:** ∗ Department of Surgery and Cancer, Imperial College, London, UK; † Department of Metabolism, Digestion and Reproduction, Imperial College, London, UK; ‡ Division of Surgery, University Hospital Southampton NHS Foundation Trust, Southampton, UK; § PsychoNeuroEndocrinology Research Group, Centre for Neuropsychopharmacology, Division of Psychiatry and Computational, Cognitive and Clinical Neuroimaging Laboratory, Department of Brain Sciences, Imperial College London, Hammersmith Hospital, London, UK; ¶ Imperial College London, Department of Public Health, Imperial Clinical Trials Unit, London, UK; || Psychiatry and Computational, Cognitive and Clinical Neuroimaging Laboratory, Department of Brain Sciences, Imperial College London, UK; ∗∗ Diabetes Complications Research Center, University College Dublin, Ireland; †† Department of Community Health Sciences, College of Applied Medical Sciences, King Saud University, Riyadh, Saudi Arabia; ‡‡ Southampton Health Technology Assessment Center, University of Southampton, Southampton, UK; §§ Section of Nutritional research, Department of Metabolism, Digestion and Reproduction, Imperial College London, UK; ¶¶ Department of Endocrinology, King's College Hospital NHS Trust, London, UK; |||| University Hospital Southampton NHS Foundation Trust, Biomedical Research Center, Southampton, UK; ∗∗∗ Primary Care, Population Sciences and Medical Education, University of Southampton Medical School, Southampton, UK.

**Keywords:** duodenal-jejunal bypass liner, endobarrier, endoscopic bariatric therapies, obesity, type 2 diabetes mellitus

## Abstract

**Objective:**

The aim of this study was to examine the clinical efficacy and safety of the duodenal-jejunal bypass liner (DJBL) while in situ for 12 months and for 12 months after explantation.

**Summary Background Data:**

This is the largest randomized controlled trial (RCT) of the DJBL, a medical device used for the treatment of people with type 2 diabetes mellitus (T2DM) and obesity. Endoscopic interventions have been developed as potential alternatives to those not eligible or fearful of the risks of metabolic surgery.

**Methods:**

In this multicenter open-label RCT, 170 adults with inadequately controlled T2DM and obesity were randomized to intensive medical care with or without the DJBL. Primary outcome was the percentage of participants achieving a glycated hemoglobin reduction of ≥20% at 12 months. Secondary outcomes included weight loss and cardiometabolic risk factors at 12 and 24 months.

**Results:**

There were no significant differences in the percentage of patients achieving the primary outcome between both groups at 12 months [DJBL 54.6% (n = 30) vs control 55.2% (n = 32); odds ratio (OR) 0.93, 95% confidence interval (CI): 0.44–2.0; *P* = 0.85]. Twenty-four percent (n = 16) patients achieved ≥15% weight loss in the DJBL group compared to 4% (n = 2) in the controls at 12 months (OR 8.3, 95% CI: 1.8–39; *P* = .007). The DJBL group experienced superior reductions in systolic blood pressure, serum cholesterol, and alanine transaminase at 12 months. There were more adverse events in the DJBL group.

**Conclusions:**

The addition of the DJBL to intensive medical care was associated with superior weight loss, improvements in cardiometabolic risk factors, and fatty liver disease markers, but not glycemia, only while the device was in situ. The benefits of the devices need to be balanced against the higher rate of adverse events when making clinical decisions.

**Trial Registration:**

ISRCTN30845205. isrctn.org; Efficacy and Mechanism Evaluation Programme, a Medical Research Council and National Institute for Health Research (NIHR) partnership reference 12/10/04.

The endoluminal duodenal-jejunal bypass liner (DJBL) is an innovative medical device developed and used for the treatment of adults with type 2 diabetes mellitus (T2DM) and/or obesity. The aim underlying its conception and design was to mimic part of the impressive metabolic and weight loss effects of intestinal bypass surgical procedures such as the Roux-en-Y gastric bypass.^1^ The DJBL consists of a single use impermeable fluoropolymer sleeve which is implanted in the duodenum endoscopically and lines 60 cm of the small intestine.^2^ As a result food bypasses the proximal intestinal mucosa by traveling inside the sleeve and comes into contact with biliopancreatic secretions once it exits the sleeve. The device is normally implanted as a day case procedure under general anesthesia or sedation, and explanted electively after 12 months. It is an endoscopic treatment that could fill the large treatment gap between lifestyle/pharmacotherapy and metabolic surgery for T2DM and/or obesity.^2^

Two systematic reviews and meta-analyses of randomized controlled trials (RCTs) and observational studies have examined the clinical efficacy of the DJBL in people with obesity with/without T2DM and yielded encouraging results.^3,4^ In the first meta-analysis, which was performed predominantly on people with obesity, participants in the DJBL group lost 5.1 kg more weight than people that underwent medical care, and whilst HbA1c reduced substantially in both groups there were no significant differences between groups.^3^ The second review assessed people with obesity and T2DM and reported superior reductions in both HbA1c (1.3% or 13.3 mmol/mol) and weight (total body weight loss 18.9%).^4^ The safety profile of the DJBL was considered acceptable and consisted predominantly of self-limiting gastrointestinal side effects. However, the meta-analyses demonstrated significant risk of bias and/or heterogeneity, and called for larger RCTs with longer follow-up.

Our aim was to determine the position of this device in the treatment algorithm for patients with T2DM and obesity. We thus conducted the largest RCT in the field to compare the clinical efficacy and safety of intensive medical care with the DJBL versus intensive medical care alone on glycemic control in people with T2DM and obesity whilst the device is in situ for 12 months and for the 12 months after explantation.

## METHODS

### Study Design

This was an open-label RCT conducted between November 2014 and January 2019 in 2 academic clinical centers in the UK, Imperial College London and University Hospital Southampton NHS Foundation Trust. The protocol has been published previously and can be reviewed within Supplemental Digital Content 1.^5^ The trial was funded by the NIHR, sponsored by Imperial College London and managed by the Imperial Clinical Trials Unit. The trial was approved by the Fulham Research Ethics Committee (reference 14/LO/0871) and conducted in accordance with the Declaration of Helsinki.

### Patients

Male and female participants, aged 18 to 65 years, with a BMI 30 to 50 kg/m^2^ and confirmed diagnosis of T2DM for at least 1 year, who had inadequate glycemic control (HbA1c 7.7%–11.0% or 58–97 mmol/mol) and were on glucose-lowering medications, excluding insulin, were eligible for the trial. Following written informed consent, 170 participants were randomized via the InForm database system at a 1:1 ratio, stratified by both site and BMI subgroup (30–40 and 40–50 kg/m^2^) to either intensive medical care *with* or intensive medical care *without* the DJBL (control group). A block randomization scheme with a random sequence of block sizes was used to ensure a balanced distribution of participants within each treatment arm.

### Procedures

Participants in both arms received dietary and physical activity counselling.^5^ Everyone was advised to follow a low-calorie diet which was based on daily amounts of 1200 to 1500 calories for women and 1500 and 1800 calories for men. In addition, it was recommended to eat regularly every day (5 times/ day), to control portion sizes, and intake of carbohydrates/starchy foods, to increase the intake of low glycemic index (GI) and high protein foods, as well as vegetables, and to reduce the intake of foods high in fat, sugar, and alcohol. Participants were also advised to include more physical activity in their daily routine for example to walk more every day. Their goal included 150 minutes (2.5 hours) a week of moderate intensity and 75 minutes a week of vigorous intensity aerobic activity and muscle strengthening activities >2 days a week. Any exercise was adjusted to individual needs and activity levels. Throughout the study, motivation, dietary compliance, and the average daily level of physical activity were recorded. The use of glucose-lowering medications was optimized by three Consultant Diabetologists and reflected best practice at the time of assessment in accordance with the guidelines of the American Diabetes Association.^6–9^ Preference was given to medications associated with weight loss or weight neutrality. Liraglutide 1.8 mg daily and dipeptidyl peptidase-4 (DPP-4) inhibitors were used throughout the trial and from 2015 onwards sodium-glucose co-transporter-2 (SGLT2) inhibitors were also used. Participants had the DJBL device implanted as a day case under a general anesthetic. The device was electively explanted after 12 months. Following explantation, participants were followed up for a further 12 months. Assessments took place at the NIHR Imperial and Southampton clinical research facilities.

### Outcomes

The primary outcome was an HbA1c reduction of ≥20% at 12 months post intervention in accordance with International Diabetes Federation guidelines.^10^ Prespecified secondary endpoints included HbA1c <6% (or <42 mmol/mol), blood pressure <135/85, total body weight loss ≥15%, reduction in the number of medications and rates of adverse events.

### Statistical Analyses

Primary and secondary outcomes were analyzed to investigate treatment effect using multivariate logistic regression, including for stratification variables (BMI group and site). To detect a success rate of 35% in the DJBL group versus 15% in the control group with 80% power, 170 subjects were randomized (full details in Supplemental Digital Content 2). Missing data implications for the primary endpoint analysis were assessed using multiple imputation methods alongside a per-protocol analysis to assess treatment adherence. Exploratory analyses were also undertaken using a mixed-model approach alongside post-hoc multivariate regression and correlation analyses. Statistical tests were carried out using SAS v9.4 and were 2-tailed with a 5% significance level and performed according to the intention-to-treat principle with results presented as mean ± SD (unless specified otherwise). To ensure data integrity, a data monitoring and ethics committee (DMEC) met every 6 months to review study progress. Full details of all statistical methodology can be found in the Statistical Analysis Plan within Supplemental Digital Content 2.

## RESULTS

**FIGURE 1:**
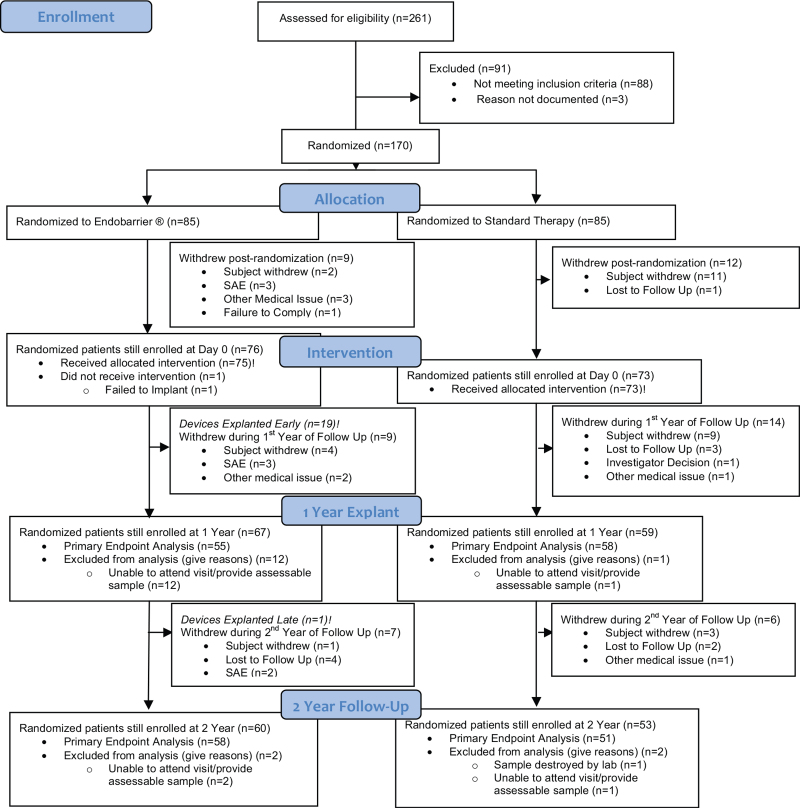
CONSORT Diagram.

A total of 170 participants underwent randomization from March 2015 through December 2016. Eight patients in the DJBL and 14 in the control group dropped out during the first 12 months, whereas 18 and 7 withdrew between months 12and 24. For the primary analysis; 55 and 58 patients (DJBL and control arms, respectively) were included within the ITT population at year 1 with 58 and 51 patients at year 2 (Fig. 1). Of those randomized, 54% were male, with a mean (±SD) age of 52 (±8) years; BMI 36.3 (±4.6) kg/m^2^, HbA1c 72 (±10) mmol/mol/8.8 (±0.9) %, and median (interquartile range) duration of T2DM 7.2 (4.0–10.2) years (Table 1).

**TABLE 1:** Baseline Characteristics^∗^

	DJBL (N = 85)	Control (N = 85)
Sex		
Male	46 (54%)	46 (54%)
Female	39 (46%)	39 (46%)
Age, y	51.6 (7.9)	51.9 (8.5)
Ethnic origin		
White	70 (82%)	62 (73%)
Black	3 (4%)	13 (15%)
Asian	11 (13%)	9 (11%)
Other	1 (1%)	1 (1%)
Weight, kg	107.9 (17.1)	104.2 (14.9)
Waist circumference, cm	118.7 (12.3)	117.8 (16.0)
Body mass index, kg/m^2^	36.8 (5.0)	35.8 (4.2)
HbA1c, mmol/mol	73.7 (10.3)	71.2 (9.7)
HbA1c (%)	8.9 (0.9)	8.7 (0.9)
Duration of T2DM, y	7.1 (4.4)	7.8 (4.5)
No. of T2DM b control medications taken, median (IQR)	2 (1–2)	2 (1–3)
Patients with hypertension at baseline, n (%)	50 (59%)	53 (62%)
Systolic blood pressure, mm Hg	130.3 (11.6)	133.3 (15.0)
Diastolic blood pressure, mm Hg	82.0 (9.7)	83.5 (10.6)
Total cholesterol, mmol/L	4.55 (0.96)	4.42 (1.00)
High-density lipoprotein cholesterol, mmol/L	1.15 (0.25)	1.15 (0.30)
Low-density lipoprotein cholesterol, mmol/L	2.47 (0.85)	2.43 (0.91)
Triglycerides, mmol/L	2.02 (1.09)	1.85 (0.82)
Aspartate transaminase, IU/L	28.7 (12.8)	28.0 (11.9)
Alanine aminotransferase, IU/L	41.1 (24.1)	37.6 (20.2)
Alkaline phosphatase, IU/L	87.7 (25.1)	89.6 (24.7)

∗ Baseline values taken at visit 3 or nearest preceding visit. Unless units are stated values are presented as mean (SD).

At 12 months, 30 of 55 participants (54.5%) achieved a 20% reduction in HbA1c in the DJBL group, compared to 32 of 58 (55.2%) in the control group [odds ratio (OR) 0.93, 95% confidence interval (CI): 0.44–2.0; *P* = 0.85] (Table 2). At the 24-month visit (ie, 12 months post explantation) 23 of 58 (39.7%) participants in the DJBL group achieved ≥20% reduction in HbA1c levels compared to 19 of 52 (36.5%) in the control group (OR 1.1, 95% CI: 0.52–2.5; *P* = 0.75). Results were unaffected by the per protocol analysis, with 29 of 54 (53.7%) patients achieving the endpoint in the DJBL group, and 29 of 52 (55.8%) in the control group (OR 0.93, 95% CI: 0.39–2.3; *P* = 0.88). Missing data (assumed missing-at-random) did not appear to have any bearing on the outcome of the primary endpoint analysis with multiple imputation using chained equations (MICE) across 50 iterations returning an estimated treatment effect on proportion of 0.025 (95% CI: −0.34 to 0.39; *P* = 0.89). Likewise, the endpoint rate required within the missing data to establish a change in the primary analysis result is significantly different when compared to the current endpoint rate in both treatment arms (Supplemental Digital Content 3).

**TABLE 2:** Primary and Secondary End Points at 12 and 24 months^∗^

	DJBL	Control	*P*
Primary endpoint—HbA1c			
Patients who achieved reduction of 20% at 12 mo	30 (54.6%)	32 (55.2%)	0.85^†^
Patients who achieved reduction of 20% at 24 mo	23 (39.7%)	19 (36.5%)	0.75^†^
12-mo HbA1c, mmol/mol	57.4 (12.9)	57.3 (13.7)	0.50^‡^
24-mo HbA1c, mmol/mol	64.8 (15.3)	62.6 (12.9)	0.71^‡^
Secondary endpoints—HbA1c			
Patients with HbA1c <42 mmol/mol at 12 mo	6 (10.9%)	4 (6.9%)	0.28^†^
Patients with HbA1c <42 mmol/mol at 24 mo	3 (5.2%)	0 (0.0%)	N/A
Secondary endpoints—weight			
Patients who achieved reduction of 15% at 12 mo	16 (24.2%)	2 (3.7%)	0.007^†^
Patients who achieved reduction of 15% at 24 mo	3 (5.2%)	1 (1.9%)	0.39^†^
12-mo Weight, kg	96.1 (16.1)	96.7 (14.4)	<0.001^‡^
24-mo Weight, kg	100.7 (16.2)	98.0 (14.7)	0.76^‡^
Secondary endpoints—rates of hypertension and blood pressure			
Patients who were nonhypertensive at 12 mo	45 (68.2%)	24 (44.4%)	0.01^†^
Patients who were nonhypertensive at 24 mo	31 (53.5%)	33 (63.5%)	0.42^†^
12-mo Systolic blood pressure, mm Hg	123.8 (14.1)	132.7 (14.8)	0.004^‡^
24-mo Systolic blood pressure, mm Hg	130.6 (16.2)	125.7 (13.9)	0.07^‡^
12-mo Diastolic blood pressure, mm Hg	77.7 (9.3)	81.5 (9.2)	0.02^‡^
24-mo Diastolic blood pressure, mm Hg	80.1 (9.9)	78.1 (8.4)	0.21^‡^
Secondary endpoints—diabetes medication			
No. of medications taken at 12 mo, median (IQR)	2 (1–3)	2 (1–3)	0.37^§^
No. of medications taken at 24 mo, median (IQR)	2 (1.5–3)	2 (1–3)	0.55^§^

IQR indicates interquartile range.

∗ Above figures are derived from the intention-to-treat population. Unless units are stated values are presented as mean (SD).

† *P* value derived from Logistic Regression model testing value at timepoint against treatment group, adjusting for covariates Site and BMI Group.

‡ *P* value is derived from testing the fixed effect for treatment group in a mixed-model analysing absolute value at timepoint adjusted for fixed effect covariates; baseline, age, BMI group, site and a random effect for intercept.

§ *P* value derived from regression model testing number of medications at timepoint against treatment group, adjusting for covariates Site and BMI Group.

**FIGURE 2:**
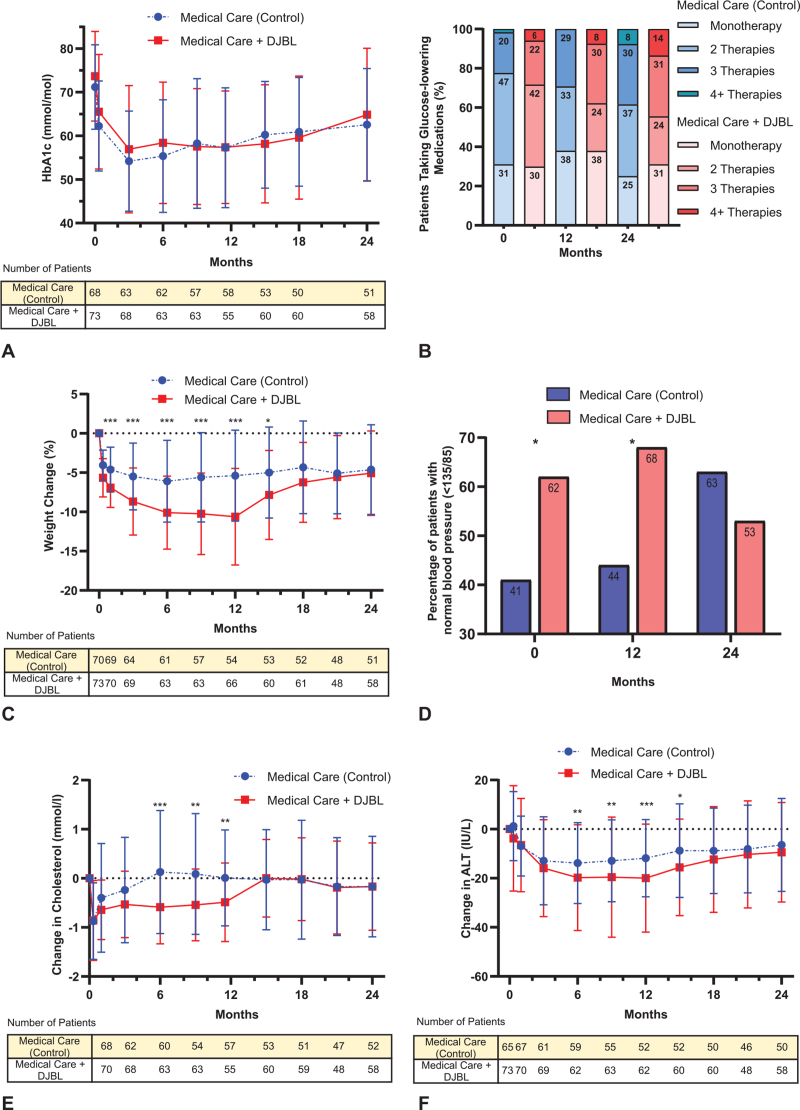
Changes in measures of diabetes control. ^∗^*P* < 0.05, ^∗∗^*P* < 0.01, ^∗∗∗^*P* < 0.001; *P* value is derived from testing the fixed effect for treatment group in a mixed-model analyzing absolute value at time point adjusted for fixed effect covariates; baseline, age, BMI group, site and a random effect for intercept. Data shown in plots A, C, E, F comprise of mean ± SD. A, absolute HbA1c values by treatment group over time. B, percentage of patients taking x number of glucose-lowering medications at M0, M12, and M24 by group. C, percentage weight-change values by treatment group over time. D, proportion of patients with normal blood pressure (≤135/85) at M0, M12, and M24 by group. E, Change in total cholesterol concentration values by treatment group over time. F, change in serum ALT concentration values by treatment group over time.

Over time, both treatment groups displayed a reduction in HbA1c levels with the greatest reduction at 3 months (Table 2, Figure 2A, eFigure 2A (Supplemental Digital Content 3)). However, there were no significant differences in the absolute reduction of HbA1c between the groups at either 12 [DJBL −15.9 ± 10.8 mmol/mol (1.5 ± 1.0%) vs control −13.3 ± 14.0 mmol/mol (−1.2% ± 1.3%), *P* = 0.50] or 24 months [DJBL −8.6 ± 15.8 mmol/mol (−0.8% ± 1.4%) vs control −8.0 ± 12.6 mmol/mol (−0.7% ± 1.2%), *P* = 0.71]. At 12 months, 6 (10.9%) patients achieved an HbA1c level of <42 mmol/mol in the DJBL group, compared with 4 (6.9%) patients in the control group. Using logistic regression, adjusting for the stratification variables of site and BMI group, the OR estimate for achieving this target in the DJBL group compared with the control group was 2.15 (95% CI 0.54–8.55; *P* = 0.28).

Post explant, at 15, 18, and 24 months, the numbers of patients who reached this HbA1c remission threshold in the DJBL group and the control group were 3 (5.0%) and 2 (3.8%), 3 (5.0%) and 2 (4.0%), and 3 (5.2%) and 0 (0.0%), respectively. There were no significant differences between the groups in the number of glucose-lowering medications used at baseline, 12 months or 24 months post-intervention (Table 2, Fig. 2B).

At 12 months, 16 of 66 participants (24.2%) achieved a ≥15% weight loss in the DJBL group, compared to 2 of 54 (3.7%) in the control group (OR 8.3, 95% CI: 1.8–39; *P* = 0.007; Table 2). However, there were no significant differences between the 2 groups at 24 months. Thirty-eight of 66 (57.6%) achieved a weight loss of ≥10% in the DJBL group, compared to 12 of 54 (22.2%) in the control group at 12 months (OR 4.50, 95% CI: 1.99–10.18; *P* < 0.001). However, there were no significant differences between the 2 groups at 24 months. DJBL resulted in significantly more weight loss at 12 months {DJBL −10.6% ± 6.2% vs control −5.4% ± 5.8%, *P* < 0.001; [Table 2, Fig. 2C, eFigure 2B, eFigure3 (Supplemental Digital Content 3)]}. At 24 months, there were no significant differences in weight loss between the two groups (DJBL −5.1% ± 5.4% vs control −4.6% ± 5.7%, *P* = 0.76).

At 12 months, 45 of 66 participants (68.2%) achieved a blood pressure below 135/85 mm Hg in the DJBL group, compared to 24 of 54 (44.4%) in the control group (OR 2.6, 95% CI: 1.2–5.5; *P* = 0.01, Table 2). At 24 months, 31 of 58 (53.5%) people in the DJBL group achieved this outcome compared to 33 of 52 (63.5%) in the control group (OR 0.72, 95% CI: 0.33–1.6; *P* = 0.42, Fig. 2D). Systolic and diastolic blood pressure were reduced significantly more in the DJBL compared to the control group at 12 months (systolic blood pressure: −6.83 ± 17.75 vs −1.04 ± 15.16 mm Hg, respectively, *P* = 0.004; diastolic blood pressure: −3.88 ± 9.81 vs −2.19 ± 11.98 mm Hg, respectively, *P* = 0.02), but not 24 months (Table 2).

At 12 months, total cholesterol concentration was reduced significantly more in the in DJBL compared to the control group (−0.49 ± 0.80 vs −0.01 ± 0.98 mmol/L; *P* = 0.009, Table 3, Fig. 2E), but there were no significant differences between groups at 24 months. High-density lipoprotein (HDL) cholesterol concentration was reduced in the DJBL group but increased in the control group at 12 months (−0.04 ± 0.16 vs + 0.12 ± 0.24 mmol/L; *P* < 0.001, Table 3). There were no significant differences between groups in low-density lipoprotein (LDL) cholesterol (−0.24 ± 0.62 vs −0.02 ± 0.84 mmol/L) or triglycerides (−0.33 ± 1.26 vs −0.13 ± 1.11 mmol/L) at 12 months (Table 3). There were no differences between the groups in LDL or HDL cholesterol and triglyceride concentrations at 12 or 24 months.

**TABLE 3:** Fasting Plasma Lipid and Liver Function Concentrations at 12 and 24 Months^∗^

	DJBL	Control	*P* ^†^
Lipids			
Total cholesterol			
12-mo cholesterol, mmol/L	4.07 (0.95)	4.36 (0.98)	—
24-mo cholesterol, mmol/L	4.41 (0.86)	4.19 (0.89)	—
12-mo change from baseline in cholesterol, mmol/L	−0.49 (0.80)	0.01 (0.98)	0.009
24-mo change from baseline in cholesterol, mmol/L	−0.17 (0.89)	−0.17 (1.03)	0.31
HDL cholesterol			
12-mo HDL, mmol/L	1.14 (0.30)	1.29 (0.32)	—
24-mo HDL, mmol/L	1.26 (0.26)	1.21 (0.30)	—
12-mo Change from baseline in HDL, mmol/L	−0.04 (0.16)	0.12 (0.24)	<0.001
24-mo Change from baseline in HDL, mmol/L	0.10 (0.15)	0.04 (0.22)	0.04
LDL cholesterol			
12-mo LDL, mmol/L	2.23 (0.76)	2.33 (0.88)	—
24-mo LDL, mmol/L	2.40 (0.70)	2.15 (0.74)	—
12-mo Change from baseline in LDL, mmol/L	−0.32 (0.62)	−0.02 (0.84)	0.09
24-mo Change from baseline in LDL, mmol/L	−0.15 (0.74)	−0.13 (0.82)	0.30
Triglycerides			
12-mo Triglycerides, mmol/L	1.72 (0.80)	1.84 (1.53)	—
24-mo Triglycerides, mmol/L	1.62 (0.74)	1.85 (1.04)	—
12-mo Change from baseline in triglycerides, mmol/L	0.33 (1.26)	−0.13 (1.11)	0.63
24-mo Change from baseline in triglycerides, mmol/L	−0.32 (1.03)	0.20 (0.72)	0.32
Liver function tests			
ALP			
12-mo ALP, IU/L	89.5 (25.20)	77.8 (22.77)	—
24-mo ALP, IU/L	82.2 (25.35)	82.1 (24.31)	—
12-mo Change from baseline in ALP, IU/L	2.2 (18.95)	−11.7 (16.97)	<0.001
24-mo Change from baseline in ALP, IU/L	−4.5 (13.63)	−8.6 (15.53)	0.21
ALT			
12-mo ALT, IU/L	21.6 (12.49)	28.5 (11.95)	—
24-mo ALT, IU/L	30.9 (21.34)	34.7 (23.66)	—
12-mo Change from baseline in ALT, IU/L	−20.0 (22.03)	−11.8 (15.72)	<0.001
24-mo Change from baseline in ALT, IU/L	−9.4 (20.28)	−6.5 (18.94)	0.32
AST			
12-mo AST, IU/L	20.7 (6.85)	23.1 (7.74)	—
24-mo AST, IU/L	24.1 (10.78)	25.7 (9.27)	—
12-mo Change from baseline in AST, IU/L	−9.3 (14.65)	−6.6 (9.23)	0.003
24-mo Change from baseline in AST, IU/L	−6.0 (12.68)	−3.4 (10.80)	0.07

ALP indicates alkaline phosphatase.

∗ Above figures are derived from the intention-to-treat population. Unless units are stated values are presented as mean (SD).

† *P* value is derived from testing the fixed effect for treatment group in a mixed-model analysing absolute value at timepoint adjusted for fixed effect covariates; baseline, age, BMI group, site, and a random effect for intercept.

At 12 months, both serum alanine aminotransferase (ALT) and aspartate aminotransferase (AST) concentrations were reduced significantly more in the DJBL compared to the control group at 12 months (ALT: −20.0 ± 22.0 vs −11.8 ± 15.7 IU/L, *P* < 0.001; AST: −9.3 ± 14.7 vs –6.6 ± 9.2 IU/L, *P* = 0.003, Table 3, Fig. 2F). There were no significant differences in ALT or AST concentrations between the groups at 24 months (ALT: −9.4 ± 20.1 vs −6.5 ± 18.9 IU/L, *P* = 0.32; AST: −6.0 ± 12.7 vs −3.4 ± 10.4 IU/L, *P* = 0.07).

A total of 857 adverse events were reported among 151 (89%) of randomized subjects. Fifty of these were serious adverse events (SAEs), which occurred among 39 (23%) subjects [Table 4, eTable 1 (Supplemental Digital Content 3)]. Of the 50 SAEs, 45 (90%) were reported in the DJBL and 5 in the control group. Of the 45 SAEs in the DJBL group, 26 (58%) were attributed to the intervention. Of the 5 SAEs in the control group, none were attributed to the intervention. There were 19 early explantations in the DJBL group. The majority of these were due to migration of the device (7), abdominal pain (5), upper gastrointestinal bleeding (2), cholecystitis (2), liver abscess (1), anticoagulation (1), and withdrawal of consent (1). A total of 8 torn devices were noted on explantation. The clinical outcomes of these 8 patients were similar to the entire DJBL group suggesting that the tears probably took place late after implantation and were not extensive enough to impact on clinical outcomes. There was 1 case of a liver abscess requiring explantation of the device and CT-guided drainage with the patient subsequently making a full recovery. In 1 patient, the device could not be removed endoscopically due to technical difficulties and required laparoscopic removal with no permanent sequelae.

**TABLE 4:** Serious Adverse Events^∗^

Event	DJBL (N=85)	Control (N = 85)
Gastrointestinal disorders		
Abdominal pain	9 (11%)	—
Vomiting	4 (5%)	—
Nausea	2 (2%)	—
Device issues		
Device malfunction	8 (9%)	—
Device migration	7 (8%)	—
Surgical and medical procedures		
Cardioversion	1 (1%)	—
Dental operation	.	1 (1%)
Renal stone removal	1 (1%)	—
Spinal decompression	1 (1%)	—
Thyroidectomy	.	1 (1%)
Vaginal hysterectomy	1 (1%)	—
Renal and urinary disorders		
Nephrolithiasis	1 (1%)	—
Pyelonephritis	1 (1%)	—
Renal colic	1 (1%)	—
Ureterolithiasis	1 (1%)	—
Cardiovascular disorders		
Acute coronary syndrome	1 (1%)	—
Stroke	.	1 (1%)
Ventricular fibrillation^†^	1 (1%)	—
Investigations		
Laparoscopy	1 (1%)	—
Liver function test abnormal	1 (1%)	—
Nuclear magnetic resonance imaging abnormal	1 (1%)	—
Hepatobiliary disorders		
Acute cholecystitis	1 (1%)	—
Liver abscess	1 (1%)	—
Metabolism and nutrition disorders		
Dehydration	2 (2%)	—
Vascular disorders		
Upper gastrointestinal hemorrhage	2 (2%)	—
Respiratory, thoracic, and mediastinal disorders		
Pneumonia	1 (1%)	—
General disorders and administration site conditions		
Surgical failure^‡^	1 (1%)	—
Infections and infestations		
Sepsis	1 (1%)	—
Musculoskeletal and connective tissue disorders		
Clavicle fracture	1 (1%)	—
Nervous system disorders		
High-grade glioma	.	1 (1%)
Skin and subcutaneous tissue disorders		
Shingles	.	1 (1%)

∗ Figures are per patient with percentage in parentheses. In the event where a reported SAE contains>1 affliction both classes/terms have been counted.

† Patient had no coronary disease but severely dilated and impaired LV function. He responded well to direct current cardioversion and Amiodarone.

‡ Failed DJBL removal at planned 12-month visit. At the time of gastroscopy, food debris was present in the stomach and first part of the intestine. The device sleeve was visible in D2/D3 but the crown was completely obscured by food debris despite multiple washings and probing. It was deemed unsafe to proceed safely and so the procedure was rebooked.

## DISCUSSION

In this trial, the addition of the DJBL to an intensive medical intervention was not associated with significantly higher rates of participants achieving a ≥20% reduction in HbA1c. However, participants in the DJBL group lost significantly more weight than patient in the control group at 12 months. The percentage of participants achieving a clinically meaningful reduction in weight of 15%, was 6 times higher in the DJBL compared to the control group at 12 months. Participants in the DJBL group also experienced superior reductions in blood pressure, serum total cholesterol, ALT and AST at 12 months. The beneficial effects of the DJBL on weight and cardiometabolic markers dissipated following explantation, with only marginal differences between the groups at 24 months. Nevertheless, both groups sustained part of their achievements in terms of HbA1c and weight loss reductions at 24 months, thus demonstrating the effectiveness of the intensive behavioural modification programme. There were significantly more adverse events in the DJBL group.

We were surprised to not detect a significant difference in glycemic control between the 2 groups. This finding is in line with the first meta-analysis on the DJBL, but contradicts the findings of the most recent meta-analysis, in which the DJBL was superior to behavioral modification both in terms of glycemia and weight loss.^3,4^ Indeed, the DJBL was originally conceived as a metabolic rather than an obesity intervention. An explanation of our findings could be the rapid improvements in the modern management of T2DM which has been revolutionized in the last few years. The combination of the intensive lifestyle modification with pharmacotherapy might have achieved a glucose-lowering “floor effect,” thus limiting our ability to detect any additional beneficial effects of the DJBL. This combination of impactful interventions was not available when previous studies were conducted.

Participants in the DJBL group experienced statistically superior and clinically relevant improvements in cardiometabolic risk factors including blood pressure, plasma lipid concentrations, and also markers of nonalcoholic fatty liver disease. These took place while the device was in situ and then gradually dissipated after explantation.

There were more adverse events in the DJBL group with rates similar what has been reported previously.^3,4^ The majority of AEs associated with the DJBL were classified as mild to moderate and occurred within the first few weeks of receiving the implant. The most common were abdominal pain and nausea. All participants made a full recovery, including those who experienced SAEs. The early explantation rate is in keeping with previous studies.^11,12^ There was 1 case of a liver abscess in the 75 successful implantations performed (1.3%). This complication rate is similar to post-marketing surveillance data and lower than the 3.5% rate of liver abscesses that led to the discontinuation of the ENDO trial (NCT01728116) in the United States in 2015. The liver abscesses in that trial also settled without the need for surgical intervention and with no long-term sequelae. After review of the relevant safety data, the FDA and Institutional Review Board approved the new STEP-1 pivotal trial (NCT04101669) of the Endobarrier in the United States in February 2019. The trial is actively enrolling participants. The manufacturers have also made technical modifications and reacquisition of CE mark status in Europe and the Middle East is expected in the first half of 2020.

The strengths of this trial include its randomized design, sample size, 12- and 24-month follow-up, multidisciplinary care and delivery of a truly intensive medical intervention, and conduct across 2 trial sites. The main limitation is the open-label design which could be a source of bias. High-dose Liraglutide (Saxenda) and weekly GLP-1 receptor agonists were not used during the trial, the former because it not reimbursable by the National Health Service and the latter because they were not available at the time. Likewise, although the withdrawal rate was higher than forecast, the primary finding from the trial held robustly when testing for missing data effects.

Endoscopic interventions offer the opportunity to fill the treatment gap between medical and surgical interventions for T2DM and obesity for people who do not have access or do not wish to undergo metabolic/bariatric surgery.^13^ Medical devices can also be used for people who urgently need weight loss and metabolic optimization before a life-changing procedure like an organ transplant or joint replacement surgery. Although the safety profile of the DJBL was similar to what has been previously reported, the rates of serious adverse events or adverse events leading to early explantation remain high in absolute terms. These will need to be reduced through manufacturing modifications for the device to become more acceptable to patients and competitive in the current T2DM treatment landscape.

In conclusion, this trial has demonstrated that the addition of the DJBL to an intensive medical intervention for people with T2DM and obesity results in superior weight loss, improvements in cardiometabolic risk factors and markers of fatty liver disease, but not glycemia, compared to the intensive medical intervention alone. These differences were observed only for the 12 months the device was in situ. The benefits of the device need to be balanced against the rate of adverse events when making clinical decisions.

## Supplementary Material

Supplemental Digital Content

## Supplementary Material

Supplemental Digital Content

## Supplementary Material

Supplemental Digital Content
