# Supplementary material for: Duodenal-Jejunal Bypass Liner for the management of Type 2 Diabetes Mellitus and Obesity: A Multicenter Randomized Controlled Trial
Source: Ann Surg. 2021 Jun 15;275(3):440–7. doi: 10.1097/SLA.0000000000004980 (PMC8820769; doi:10.1097/SLA.0000000000004980)
Supplement: Supplemental Digital Content [file ansu-275-0440-s001.pdf]

CLINICAL STUDY PROTOCOL

(ICTU Adopted)

Study Title: A randomized controlled trial of a duodenal sleeve bypass device (EndoBarrier) compared with standard medical therapy for the management of obese subjects with type 2 diabetes

Protocol Number: 14SM2015

Product: EndoBarrier TM Gastrointestinal Liner

Sponsor: Imperial College London

Protocol Date: 5th March 2018

Property of Imperial College London:

May not be used, divulged or published without the consent of Dr Julian Teare:

St. Mary's Hospital, Praed Street, London, W2 1NY

CONTACT LIST

Chief Investigator

Dr Julian Teare
Imperial College London
St Mary's Campus
St Mary's Hospital
CL3 026, Clarence Wing
Praed Street
London
W2 1NY
Tel: +44 (0) 20 3312 1072
Fax: +44 (0) 20 7724 1641

Contact Person:
Dr Michelle Sleeth/Dr Christina Prechtl
Tel: +44 (0) 78 7285 0052 or +44 (0)207 594 5946

Sponsor

Imperial College London
Faculty of Medicine Centre
Joint Research Office
Room 221
Medical School Building
St Marys Campus
Norfolk Place
London W2 1PG
Tel: +44(0)203 311 0204
Fax: +44(0)203 311 0203

Contact person:
Mrs Ruth Nicholson
Tel: +44 (0)203 311 0212
Fax: +44(0)203 311 0203

Funder

National Institute for Health Research
Evaluation, Trials and Studies Coordinating Centre
University of Southampton
Alpha House, Enterprise Road
Southampton SO16 7NS

ICTU Study Manager

Dr Christina Prechtl
Imperial College London
Imperial Clinical Trials Unit
Stadium House
68 Wood Lane
London
W12 7RH
Tel: +44 (0)207 594 5946

Device Manufacturing Facilities

GI Dynamics, Inc.
1 Maguire Road
Lexington, MA 02421 - USA
Tel: +1 (781) 357-3300
Fax: +1 (781) 357-3301

Contact person:
Mr Sean Holmes

ABBREVIATIONS

AE	Adverse Event
CI	Chief Investigator
CRF	Case Report Form
DMC	Data Monitoring and Ethical Committee
EB	EndoBarrier
GMP	Good Manufacturing Practice
ICTU	Imperial Clinical Trials Unit
IEC	Independent Ethics Committee
IMP	Investigational Medicinal Product
IDF	International Diabetes Federation
IRB	Institutional Review Board
PPI	Proton Pump Inhibitor
QA	Quality Assurance
SAE	Serious Adverse Event
SAP	Statistical Analysis Plan
SOP	Standard Operating Procedure
SSAR	Suspected Serious Adverse Reaction
SUSAR	Suspected Unexpected Serious Adverse Reaction
TSC	Trial Steering Committee
T2DM	Type 2 Diabetes Mellitus

TABLE OF CONTENTS

Content	Page
1 INTRODUCTION AND RATIONALE	8
1.1 Background	8
1.2 EndoBarrier Device	8
1.3 Potential mechanism of the EndoBarrier device	9
1.4 Hypothesis	11
1.5 Risks and benefits of the EndoBarrier	11
1.6 Rationale for current study	12
2 OBJECTIVES AND ENDPOINTS	13
2.1 Primary objectives	13
2.2 Secondary objectives	13
2.3 Safety objective	13
2.4 Primary Endpoints	13
2.5 Secondary Endpoints	14
3 STUDY DESCRIPTION	14
3.1 Design	14
3.2 Treatment regimens	15
3.3 Study flow chart	16
4 STUDY POPULATION	18
4.1 Number of subjects to be studied	18
4.2 Inclusion criteria	18
4.3 Exclusion criteria	18
4.4 Withdrawal criteria	19
5 VISIT PROCEDURES, MEASUREMENTS AND ASSESSMENTS	19
5.1 Recruitment and Screening	19
5.2 Randomisation	22
5.3 Visit Schedule	22
5.4 Visit procedures	26
5.5 Description of procedures and measurements	31
5.5.1 Diabetologist/Endocrinologist Diabetes Review	31
5.5.2 EndoBarrier	33
5.5.3 Dietetic Input	35
5.5.4 Medical history and physical examination	37
5.5.5 ECG	37
5.5.6 Urine pregnancy test	37
5.5.7 Vital signs	38
5.5.8 Body weight and height	38
5.5.9 Waist circumference	38
5.5.10 Routine biochemistry and haematology	38
5.5.11 Urine albumin:creatinine ratio	39
5.5.12 Health Economics Questionnaires	39
5.5.13 DNA and RNA Sampling	41
5.5.14 Metabolomics	41

Content	Page
5.5.15 Bio-electrical impedance analysis	41
5.5.16 PPI and H. Pylori test	42
**5.6 Sub-group specific procedures and measurements**	**42**
5.6.1 Eating and behaviour questionnaires	42
5.6.2 Food diaries	43
5.6.3 Visual analogue scales	43
5.6.4 Gut hormones and metabolites	43
5.6.5 Eating behaviour computerised tasks	44
5.6.6 Disgust sensitivity questionnaire	44
5.6.7 Pregnancy test	44
5.6.8 Functional MRI	44
5.6.9 Cognitive assessment tasks	46
5.6.10 Insulin clamp	46
5.6.11 Taste and food preference assessment	47
**6 ADVERSE EVENTS (AE)**	**49**
**6.1 Adverse Event description**	**49**
**6.2 Severity of Adverse Events**	**49**
**6.3 Causality of Adverse Events**	**49**
**6.4 Adverse Event Relationship Definition in the Control Group and Sub-group 1-3**	**49**
**6.5 Abnormal Laboratory Test Results**	**50**
**6.6 Anticipated adverse events**	**50**
**6.7 MRI safety and protection of patients**	**52**
**6.8 Definition of Device Related Adverse Events**	**53**
**6.9 Adverse Event Relationship Definition in EndoBarrier Group**	**53**
**6.10 Adverse Device Effect**	**54**
**6.11 Serious Adverse Events**	**55**
**6.12 Serious Adverse Device Effect (SADE)**	**56**
**6.12.1 Anticipated Serious Adverse Device Effect (SADE)**	**56**
**6.12.2 Unanticipated Serious Adverse Device Effect (SADE)**	**57**
**6.13 Reporting of SAEs**	**57**
**7 EARLY DISCONTINUATION OF THE STUDY OR INDIVIDUAL SUBJECTS**	**58**
**7.1 Early Discontinuation of the Study**	**58**
**7.2 Early Discontinuation of Individual Subjects**	**58**
**8 STATISTICAL ANALYSES**	**59**
**8.1 Sample Size and power considerations**	**59**
**8.2 Data Analysis**	**61**
**9 DATA MANAGEMENT**	**64**
**10 TREATMENT**	**64**
**10.1 Device Product Details**	**64**
**11 REGULATORY, ETHICAL AND LEGAL ISSUES**	**65**
**11.1 Declaration of Helsinki**	**65**
**11.2 Good Clinical Practice**	**65**
**11.3 Independent Ethics Committee/Institutional Review Board**	**65**

Approval	

Content	Page
11.3.1 Initial Approval	65
11.3.2 Approval of Amendments	65
11.3.3 Annual Safety Reports and End of Trial Notification	66
**11.4 Insurance**	**66**
**11.5 Informed Consent**	**66**
**11.6 Contact with General Practitioner**	**66**
**11.7 Subject Confidentiality**	**67**
**11.8 End of Trial**	**67**
**11.9 Study Documentation and Data Storage**	**67**
**12 ADMINISTRATIVE MATTERS**	**67**
**12.1 Source Data (CRF)**	**67**
**12.2 Language**	**68**
**12.3 Data Collection**	**68**
**12.4 Electronic Recording of data**	**68**
**12.5 Study Management Structure**	**68**
12.5.1 Trial Steering Committee (TSC)	68
12.5.2 Trial Management Group	69
12.5.3 Data Monitoring & Ethical Committee (DMEC)	69
**12.6 Monitoring**	**69**
**12.7 Quality Control and Quality Assurance**	**70**
**12.8 Disclosure of data and publication**	**71**
**13 REFERENCES**	**72**
**SIGNATURE PAGE 1 (CHIEF INVESTIGATOR)**	**79**
**SIGNATURE PAGE 2 (SPONSOR)**	**80**
**SIGNATURE PAGE 3 (STATISTICIAN)**	**81**
**SIGNATURE PAGE 4 (INVESTIGATOR)**	**82**

1 INTRODUCTION AND RATIONALE

1.1 Background

Obesity is a modern pandemic. One of the major complications of obesity is the development of diabetes. In 2011 an estimated 366 million people worldwide suffered from diabetes and this number is predicted to rise to 522 million by 2030. The US Center for Disease Control found that in newly diagnosed type 2 diabetes mellitus (T2DM) 85% were overweight or obese and 54% obese[1]. Medical therapy to control diabetes can be limited. In comparison to surgery, only half of diabetic patients achieve a satisfactory control as measured by the glycosylated haemoglobin levels (HbA1c) on medical treatment[2].

There is an urgent need for effective and safe non-surgical treatments for the epidemic of obesity and T2DM. The only successful long term treatment for obesity is bariatric surgery, especially Roux-en-Y gastric bypass (RYGB) surgery. RYGB produces ~25% weight loss which is maintained for at least 20 years and improves glycaemic control in 90% of patients[3-6]. The mechanism by which RYGB achieves these dramatic benefits is unclear. The surgery combines several manipulations: (i) stomach resection producing a small pouch, (ii) bypass of duodenum and proximal jejunum, (iii) earlier contact of food with the distal ileum and (iv) disrupted bile flow. This complex procedure results in exaggerated levels of plasma anorexigenic gut hormones and bile acids[7-11]. Patients after RYGB also have lower brain responses during evaluation of high-calorie foods compared to after gastric banding surgery in which manipulations (ii) to (iv) are absent, and are less prepared to expend effort to receive high-calorie foods[11,12]. The early improvement in glycaemia after RYGB is independent of weight loss and may include changes in hepatic glucose output or insulin resistance[13,14]. It remains unclear which of these myriad components produce the profound behavioural and metabolic benefits of RYGB.

A new procedure, the EndoBarrier device offers the unique opportunity to interrogate the contribution of bypassing the proximal bowel, and the associated alteration in bile acid metabolism, in the regulation of food intake, food reward and glucose homeostasis.

1.2 EndoBarrier Device

In an attempt to avoid surgery a new device and concept has been developed called a duodenal-jejunal sleeve bypass. The duodenal-jejunal bypass sleeve device (EndoBarrier GI Dynamics Inc, Lexington MA) consists of a single use endoscopic implant. It has a removable nitinol stent anchor to affix to the wall of the duodenum to which is attached an impermeable fluoropolymer sleeve extending 60cm into the small bowel. As a result pancreatic and bile juices only mix with food after passing through the sleeve which bypasses the proximal intestinal tract.

As proof of concept, the device was first implanted in rodents and then porcine models [15] before in 2007 being inserted in an obese woman with BMI 45 kg/m^2 who lost 9kg over a three month period[16]. The next study involved 12 individuals with mean BMI 43 kg/m^2, four of whom had T2DM. Ten patients were able to retain the device for 12 weeks and achieved 23.6% excess weight loss[17],

and all four patients with T2DM had normal fasting glucose levels without hypoglycaemic medication[18]. In subsequent trials obese patients lost 11.9% excess weight compared to 2.7% for the sham arm treated with diet alone[19]. In a similar Dutch study the mean excess weight lost was 19% v 6.9% for controls. For the eight subjects with T2DM in that study six had improvements in their T2DM (reduced HbA1c levels) and one managed to achieve diabetes resolution[20].

In addition to the Imperial pilot study, the only similar study to that proposed to date is a small Dutch study. The device was implanted for a six month period with 34 patients in the treatment and control arms. At six months there was a fall of 1.3% in the HbA1c in the EndoBarrier arm and 0.8% in the control arm, showing the benefit of regular supervision and dietetic advice. This study has only been presented in abstract form (Koehestanie, P Diabetes 2012 Suppl P1183 A305). In this study only one patient required removal of the device before the end of the six month study period.

A further Chilean study implanted the device in 39 morbidly obese subjects (6 with T2DM) for 52 weeks. For those that completed the study, the average weight loss was 22kg[21]. To date only 19 subjects with T2DM have had the device in situ for a year[21,22].

Pilot data[23] and clinical observations from a cohort of patients at Imperial College London suggest that bypassing the proximal small bowel with the EndoBarrier results in:

Weight loss:
Patients reported a reduction in hunger and increased fullness, but also a shift away from calorically dense high fat and sugar foods to healthier choices similar to those changes encountered after RYGB[24,25]. Improvements in glycaemia are resulting through early reduction in hepatic insulin resistance independent of acute caloric restriction and before any weight loss took place. To quantify this, they used the Homeostasis Model of Assessment - Insulin Resistance (HOMA-IR) in the fasting state and found that in the same cohort of patients, HOMA-IR decreased as early as 1 week after implantation. HOMA-IR decreases more in the RYGB compared to caloric restriction 4 days after surgery, when minimal weight loss has taken place[13].

An early and sustained increase in insulin production:
This can be measured by the area the under the curve of glucose after a fixed meal.

Gradual reductions in peripheral (fat and muscle) insulin resistance alongside weight loss:
The pilot data showed that HOMA-IR remains low at 1 year after EndoBarrier implantation, whilst HbA1c decreased from 8.6 ± 0.2% to 7.5 ± 0.4% ($p<0.05$). These results are in line with the few other published pilot studies on the effects of the Endobarrier[19-22,26,27].

1.3 Potential mechanism of the EndoBarrier device

Evidence from mechanistic studies in patients and animal models after RYGB or duodenal jejunal bypass (DJB) suggests that the mechanism behind the behavioural and metabolic improvements resulting from the bypass of the proximal small bowel may include altered signalling within the Gut-Liver-Brain axis. The following are thought to be the most likely neural and metabolic mediators:

Vagus nerve

Undigested food in rat models of DJB stimulates nutrient sensors in the distal small bowel. These communicate unexpected presence of a large caloric load to the homeostatic regulators of food intake in the brain through vagal afferents resulting in an early decrease in hunger and increase in fullness, which eventually leads to weight loss. In response to the stimulation of the distal small bowel by undigested food, vagal efferents from the brain to the liver are also stimulated and act to reduce hepatic glucose output. The magnitude of these signals was more pronounced in the DJB rats compared to pair fed rats sham rats, therefore excluding acute post-operative caloric restriction as a mechanism [28].

Bile acids

These are increasingly recognized as potent metabolic regulators, especially when not mixed with food (undiluted). In animal and cell culture experiments bile acids decrease food intake, increase energy expenditure, decrease insulin resistance and stimulate the release of anorexigenic and incretin gut hormones from the distal intestine[29]. Recent results showed that drainage of endogenous bile into the terminal ileum of rats was associated with an enhanced satiety gut hormone response, reduced food intake, and lower body weight and that after RYGB, bile flow is altered, leading to increased plasma bile acids, incretin and satiety gut hormone concentrations[8]. These findings are supported from data from the human literature[9,10]. The presence of undiluted bile in the proximal small bowel after the EndoBarrier insertion may exert the above effects in humans through stimulation of bile acid receptors.

Anorexigenic and incretin gut hormones

Following RYGB and/or DJB, plasma levels of glucagon-like peptide-1 (GLP-1), peptide YY (PYY) and oxyntomodulin (OXM) are increased in the post-prandial state, and sometimes even fasting state[7,30-36]. These hormones have pleiotropic effects which include increasing fullness through their effects on the hypothalamus-brainstem (homeostatic control of food intake), the release of insulin by the pancreas (GLP-1 and OXM incretin effect) and the reduced activation of brain reward systems (i.e. orbitofrontal cortex, amygdala, insula, striatum – hedonic regulators of food intake and choices) as quantified by functional magnetic resonance imaging (fMRI)[37].

Altered gut microbiota

'Metabonomics' describes the generation of multiparametric metabolic information from the analysis of biofluids or tissues, combined with multivariate pattern recognition analysis. It provides an opportunity to investigate physiological characteristics or disease based on underlying metabolic processes, both in humans and in animal models[38-40].1H nuclear magnetic resonance (NMR) spectroscopy and mass spectrometry (MS) are the technologies most often employed to acquire such metabolic profiles; subsequent chemometric analysis of the complex spectra allows the extraction of maximal biochemical information from the data, and the identification of any differences between cohorts studied[41]. Recent studies show that the urinary metabolic profile of obese individuals differs from that of age-matched controls[42], and have documented alterations in the serum metabolic profile of those undergoing Roux-en-Y gastric bypass surgery[43,44]. The intestinal microbiota differs between obese and lean individuals[45]. Thus urinary metabolic profiling in the

study will allow the evaluation and monitoring of the biochemical consequences of potential alterations in the gut microbiota following Endobarrier insertion.

1.4 Hypothesis

In summary, this study hypothesises that the EndoBarrier:

Reduces weight by:

Reducing hunger and increasing fullness therefore reducing food intake similar to changes seen with RYGB[46,47], changing food preferences away from high-calorie sweet and fatty foods. Obese patients after RYGB find high-calorie foods less appealing, less palatable and have reduced associated fMRI activation of reward systems to high-calorie foods compared to patients with similar weight loss after gastric banding[11]. RYGB also reduces desire to work for high-calorie fat/sweet taste but not low-calorie taste[12]. It remains unclear which of these myriad components produce the profound behavioural and metabolic benefits of RYGB including the beneficial effects on food reward. The novel technique of the 'EndoBarrier', can answer this question since it mimics part of the RYGB procedure through bypass of the duodenum and disrupting bile flow. The incorporation of the functional MRI paradigm in this proposal will therefore help reveal for the first time if duodenal exclusion is responsible for the beneficial effects of RYGB on brain food reward responses, by longitudinal scanning of obese patients with T2DM before and after insertion of the EndoBarrier.

Improves glycaemic control by:

a) Decreasing hepatic insulin resistance (and therefore hepatic glucose output) independently of any caloric restriction and before weight loss has taken place
b) Increasing insulin production before and after weight loss has taken place
c) Gradually decreasing total body and tissue specific insulin resistance as a result of weight loss
d) Reducing visceral adiposity and hepatic and pancreatic fat content

1.5 Risks and benefits of the EndoBarrier

Benefits:

The health risks of obesity and T2DM are significant. Obese people have more than 10 times the risk of developing T2DM [48] and 3 times the risk of coronary heart disease [49] with a doubling of mortality with a BMI over 40 kg/m^2 [50]. A 50 year old person with T2DM dies on average 6 years younger than their counterpart without T2DM[51]. The study represents an attempt to improve these risks through a novel technique. The improvement of the metabolic profile of these patients will reduce their long term health risks particularly cardiovascular although this will take a much larger trial to demonstrate.

Risks:

The risks as defined in the Instructions for Use (IFU) and protocol of the CE marked EndoBarrier Gastrointestinal Liner associated procedures are anticipated as those used within the authorized indications for use.

The major risks associated with the use of the EndoBarrier Gastrointestinal Liner can be categorized into those associated with the procedural portion of the implant and explant and those associated with the device itself.

The risks associated with the implant/explant procedure include the same risks observed with other upper GI Endoscopic procedures (see adverse events section number 6.8).

The device will be inserted for a period of 12 months and then removed and the patients followed up for a further 12 months. The frequency of adverse events will be determined. The commonest adverse events to date with the EndoBarrier have been bleeding, stent migration and pain necessitating device removal. It is estimated that up to 30% will require early device removal although this was considerably less in the Dutch study.

Selecting Investigators qualified by training and experience to investigate the device will minimize the risks described above. Each Investigator will be trained in the implantation and removal of the EndoBarrier Gastrointestinal Liner.

Clinical monitoring will occur frequently and routinely to permit the timely collection of safety data. Any unforeseen risks arising during the course of the investigation will be evaluated if and when they occur, and reported in accordance with local government regulations. Action will be taken as appropriate to minimize risk.

1.6 Rationale for current study

Obesity is a serious medical condition which is increasing in incidence worldwide. Obesity induces metabolic abnormalities which contribute to the development of diabetes mellitus and cardiovascular disease. The treatment modalities currently available for the treatment of obesity (i.e. lifestyle interventions, pharmacotherapy and surgery) have limited long-lasting success in producing major and sustained weight loss and are often associated with undesirable side effects, risks or complications. Therefore the need for new exits and effective strategies is necessary to prevent and reduce obesity and its complications such as T2DM. This study is a randomised, placebo-controlled trial which has been designed to further investigate the potential of the EndoBarrier device as an effective alternative treatment to surgery and existing medical therapies in T2DM.

If the EndoBarrier is effective at achieving long-lasting weight loss and glycaemic control, there is an obvious potential for savings on future health and social care; through the avoidance of diabetes and related complications. However, the overall cost-effectiveness of the EndoBarrier device depends on the balance of health benefits, harms, costs and savings compared with other surgical and medical treatment options. In addition to a ‘within trial’ economic analysis, modelling will be used to estimate lifetime impacts on morbidity, mortality and expenditure, and hence to evaluate whether the EndoBarrier offers the NHS good value for money.

2 OBJECTIVES AND ENDPOINTS

2.1 Primary Objectives

- To compare the EndoBarrier with conventional medical therapy, diet and exercise for obesity related type II diabetes and their effectiveness on metabolic state as defined by the International Diabetes Federation (IDF) with an HbA1c reduced by 20%.

2.2 Secondary objectives

- To compare the EndoBarrier with conventional medical therapy, diet and exercise for obesity related type II diabetes and their effectiveness on:
  - Metabolic state as defined by the International Diabetes Federation (IDF) with an HbA1c < 6% (or < 42 mmol/mol)
  - Blood pressure < 135/85
  - Absolute weight loss

- To investigate the mechanism of the effect of the EndoBarrier via changes in:
  - Gut hormones
  - Microbiome
  - Appetite, food hedonics and brain reward systems
  - Body fat content
  - Food preference
  - Hepatic or peripheral insulin sensitivity
  - Bile acids
  - Biomarkers such as genetic markers

- To estimate the cost-effectiveness of the EndoBarrier device compared with conventional treatment over the trial period (within trial analysis).

- To estimate the long-term cost-effectiveness (over 24 months) of the EndoBarrier device compared with conventional treatment and alternative surgical interventions.

2.3 Safety objective

- To evaluate the safety of the EndoBarrier and frequency of adverse events

2.4 Primary Endpoints

- Reduction in HbA1c by 20%

2.5 Secondary Endpoints

- HbA1c of less than 6% (or 42 mmol/mol)
- Blood pressure below 135/85 mmHg
- Absolute weight loss greater than 15%
- Reduction in dose/or number of medications
- Frequency of Adverse Events
- Cost of interventions and related health and social care
- QALYs accrued (calculated from area under the EQ-5D curve)
- Incremental cost per QALY within the trial period and over the long-term

3 STUDY DESCRIPTION

3.1 Design

This study is a randomised controlled trial of the EndoBarrier device compared with standard medical therapy for the management of obese subjects with T2DM. Subjects will be randomised to one of the two treatment arms (see Table 1) of the study via the InForm system (the eCRF database for the study). Over 24 months, the study will be performed at two investigational sites, Imperial College Healthcare NHS Trust in London and University Hospital Southampton NHS Foundation Trust.

Individuals in both study arms will be invited regular medical check-ups including measurement of weight, blood pressure, and blood parameters (HbA1c, cholesterol, triglycerides, fasting blood glucose, insulin), quality of life (EQ-5D) and use of health services, as well as to record any adverse events and medications. Diabetes medication titrations will be conducted by the study diabetologists/endocrinologists in accordance with the guidelines of the American Diabetes Association[52] (see section 5.5.1 for full details). They will also receive routine dietary and exercise counselling as well as telephone counselling from a specialist dietitian. The control arm will be invited for routine follow-up visits to review their standard medical care. Both arms will also be invited to undergo a diversity of tests investigating the mechanism of the EndoBarrier. After 12 months, the EndoBarrier will be removed and both arms will be followed up for a remaining 12 months.

In order to investigate the mechanism of the effect of the EndoBarrier Device, the trial is divided into three sub-groups in <u>each arm</u> who will have the following additional assessments at visits 3, 5, 8 and 10, 14 (see section 5.4 and 5.6 for more detail):

- Sub-group 1 (minimum n=24): functional MRI, eating behaviour, cognitive assessment, and post-meal gut hormones
- Sub-group 2 (minimum n=18): insulin clamps

- Sub-group 3 (minimum n=18): assessment of taste and food preference, eating behaviour assessment, and post-meal gut hormones

Participation in the sub-groups is optional.

3.2 Treatment regimens

Table 1. Summary of treatment groups

Treatment Sequence	Number of subjects *	Treatment Period 1	Follow-up Period 2
1 EndoBarrier Device	80	12 months	12 months
2 Standard Medical therapy	80	12 months	12 months
Total number of subjects	160		

* Additional subjects may be randomised to allow for any randomised subject that has withdrawn prior to commencement of treatment at Visit 4.

3.3 Study flow chart

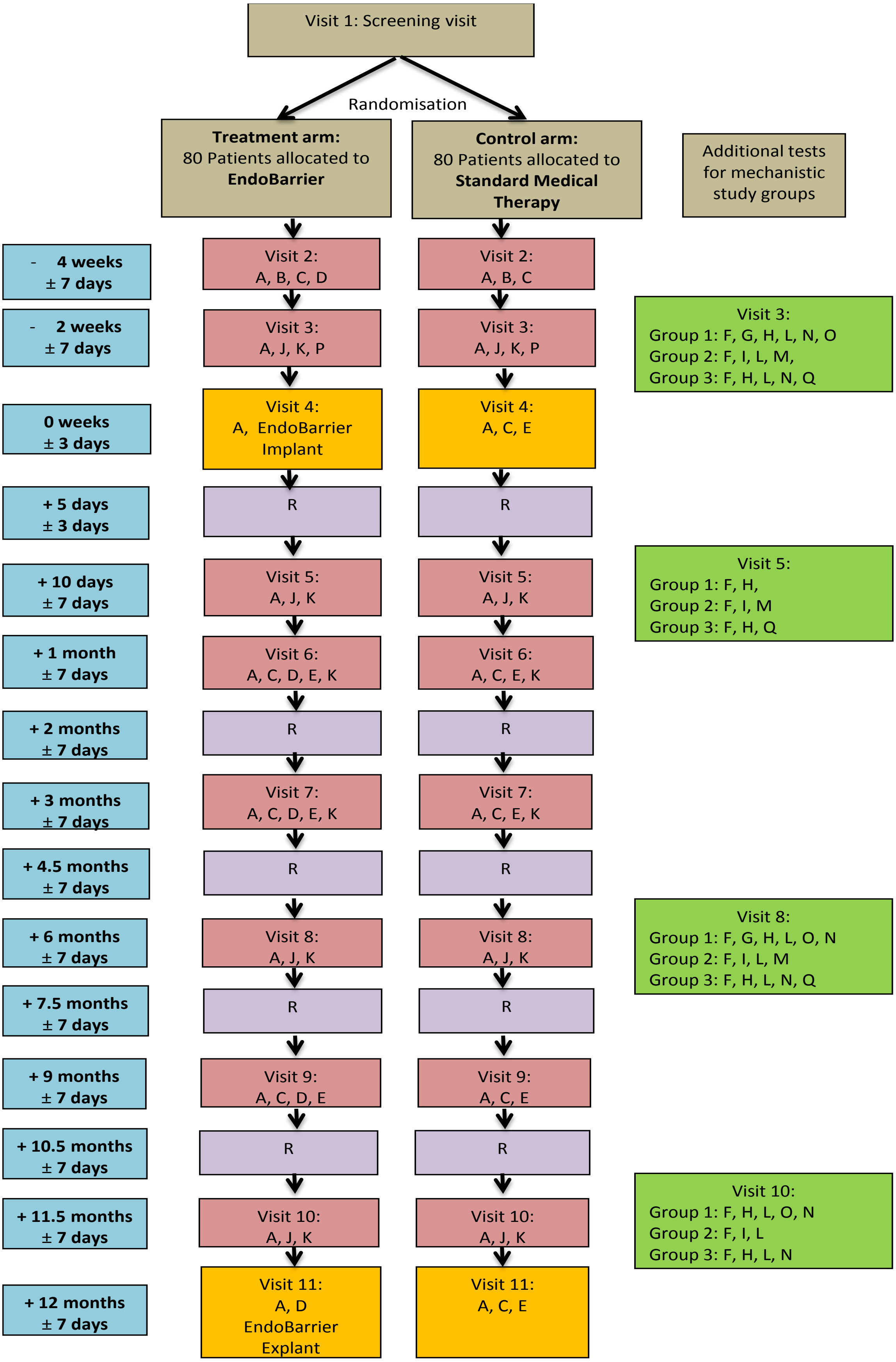

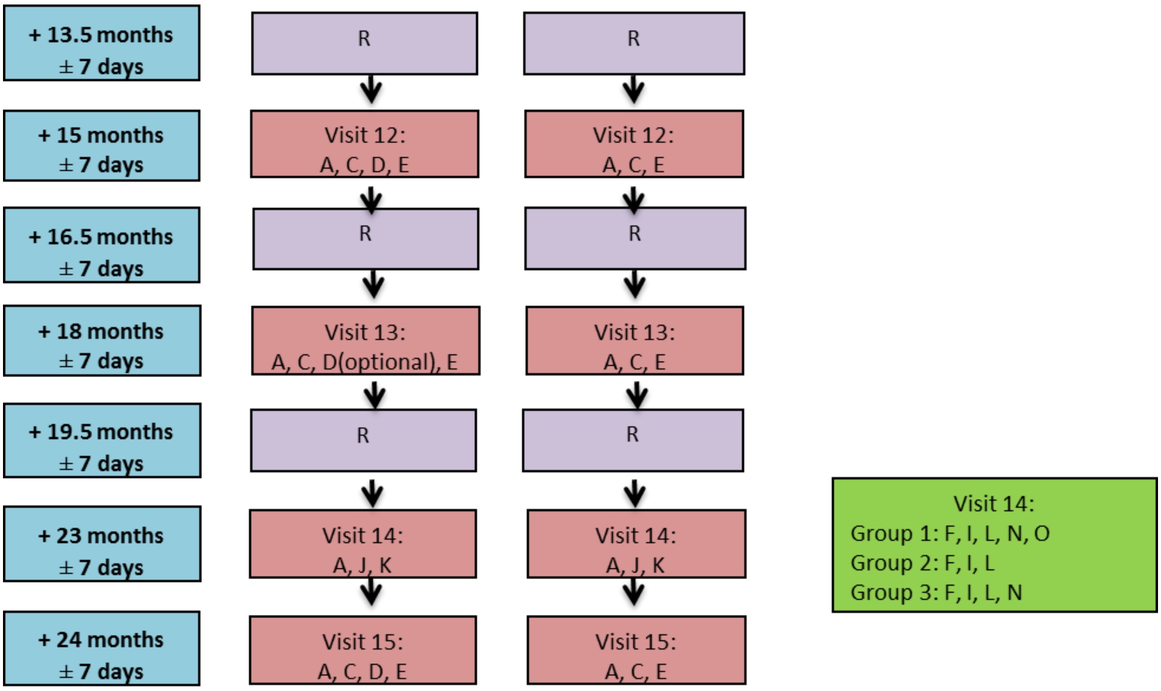

Legend:
A = Weight, waist, blood pressure, routine bloods, adverse events, changes in medication/medical history
B = Dietary Counselling
C = Medical Therapy (Diabetologist/Endocrinologist)
D = Gastroenterologist
E = Dietitian follow up
F= Bioelectrical impedance
G= fMRI
H = Gut hormones(fasting and post-meal profile)
I= Gut hormones(fasting only)
J = Metabolomics
K = Health Economics questionnaires
L = Eating and behaviour questionnaires
M = Insulin clamps
N= Eating behaviour computerised tasks
O= Cognitive assessment tasks
P= DNA Sample
Q= Food preference and taste assessment
R= Telephone counselling

4 STUDY POPULATION

4.1 Number of subjects to be studied

80 subjects are required per treatment group allowing for drop-outs: a total of 160 patients need to be randomised and start treatment (EndoBarrier device or diet and exercise) at visit 4.

4.2 Inclusion criteria

1. Age 18-65 years (male or female)
2. T2DM for at least 1 year (HbA1c 7.5-11.0% = 58-97 mmol/mol)
3. On oral T2DM medications (metformin is allowed, but not required)
4. BMI 30-50 kg/m^2

4.3 Exclusion criteria

1. Language barrier, mental incapacity, unwillingness or inability to understand and be able to complete questionnaires
2. Non-compliance with eligibility criteria
3. Females of childbearing potential who are pregnant, breast-feeding or intend to become pregnant or are not using adequate or reliable contraceptive methods
4. Evidence of absolute insulin deficiency as indicated by clinical assessment, a long duration of T2DM and a fasting plasma C-peptide of <333pmol/L
5. Current use of insulin
6. Previous diagnosis with Type 1 DM or a history of ketoacidosis
7. Requirement of NSAIDs (non-steroidal anti-inflammatory drugs) or prescription of anticoagulation therapy during the implant period
8. Current iron deficiency and/or iron deficiency anaemia
9. Symptomatic gallstones or kidney stones at the time of screening
10. History of coagulopathy, upper gastro-intestinal bleeding conditions such as oesophageal or gastric varices, congenital or acquired intestinal telangiectasia
11. Previous GI surgery that could affect the ability to place the device or the function of the implant
12. History or presence of active H. pylori (if subjects are randomised into the EndoBarrier arm and have a history or presence of active H. pylori – tested during study visit 2 - they can receive appropriate treatment and then subsequently enrol into the study)
13. Family history of a known diagnosis or pre-existing symptoms of systemic lupus erythematosus, scleroderma or other autoimmune connective tissue disorder
14. Severe liver impairment i.e. AST, ALT or gGT >4 times upper limit of the reference range or kidney impairment i.e. estimated Glomerular Filtration Rate (GFR) < 45 ml/min/1.73m2
15. Severe depression, unstable emotional or psychological characteristics (indicated by Beck Depression Inventory II score >28)
16. Poor dentition and inability to adequately chew food
17. Planned holidays up to three months following the EndoBarrier Implant
18. Previous EndoBarrier implantation

19. Metal implant unsuitable for MRI scanning and claustrophobia as contraindications for MRI scans (sub-group 1 - fMRI study only)
20. Vegetarian, vegan, gluten or lactose intolerance as unsuitable for fMRI food picture paradigm (sub-group 1 – fMRI only)

4.4 Withdrawal criteria

Subjects may be withdrawn from the study at the discretion of the Investigator or Sponsor due to a safety concern or if judged non-compliant with trial procedures.

A subject must be withdrawn from treatment if the following applies:

1. Subject chooses to withdraw from the study at any time
2. Adverse Event requiring device explant
3. Pregnancy or intention of becoming pregnant

Reasons for withdrawals and discontinuation of any subject from the protocol have to be recorded.

5 VISIT PROCEDURES, MEASUREMENTS AND ASSESSMENTS

5.1 Recruitment and Screening

Participants will be identified from a number of areas which include:

- **Registers of patients with diabetes who have consented to be contacted about future research including DARE (Diabetes Alliance for Research in England REC 2002/7/118)**

The team responsible for a register will search the system to identify suitable participants and send out an invitation letter and participant information sheet. Interested patients will be asked to contact the research team directly to find out more about the study. No patient details will be passed on to the study team.

- **Hospital or GP patient databases/notes review (Research Site or Participant Identification Centre)**

This activity will be completed by the routine care team. The medical records may be reviewed by members of the routine care team to pre-screen whether the patient may potentially be eligible for the study before the patient is approached about the study by a member of the routine care team (hospital or GP).

Potential participants will be sent an invitation letter, participant information sheet and study summary and asked to complete a reply slip, telephone or send an email to the research team. If the subject is eligible they can contact the research team to arrange an appointment for a screening visit at Imperial College London (St. Mary's Hospital) or University of Southampton (NIHR Wellcome Trust Clinical Research Facility (WTCRF)).

- **Face to face approach during routine clinic visits (Research Site or Participant Identification Centre)**

Potential participants will be identified from the clinic lists. Consultants/GPs will review the upcoming clinic list and flag any participants that should not be approached. The nurse or doctor routinely involved in the patients care will send an invitation letter ahead of the clinic or approach participants face to face during the clinic to provide the Participant Information Sheet. The nurse or doctor routinely involved in the patients care will inform patients they can contact the research team if they are interested in partaking in the study.

- **Other research studies within Imperial College, Imperial NHS Trust and the Local Clinical Research Network (LCRN)**

Other research studies run at Imperial College, Imperial NHS Trust and the LCRN can speak to and provide further information about our trial to participants who were ineligible, withdrawn or excluded from their study but consented to be contacted for possible participation in future research studies.

- **Adverts, news stories, posters, information leaflets and business cards in public areas, newspapers, websites, GP surgeries and hospital clinics areas and support groups**

- **Diabetes, Obesity and other Support groups**

- **Study websites**

- **Social media websites**

Advertisements / news stories / trial websites etc. will only contain essential documentation approved by the ethics committee (patient information sheet, trial contact details etc.). We may set up social media pages such as Twitter / Facebook which will contain basic trial contact details and referral to the website.

Members of the research team may attend support group meetings by invitation to discuss the study. Leaflets will be made available to members.

Informed consent and screening

Patients identified by PICs that would like further information on taking part in the trial will be asked by their routine care provider to contact a member of the research team in order to register their interest in the EndoBarrier trial. A copy of the study summary will then be forwarded to patients. Participants contacting the research team as a result of advertisements/websites will be forwarded a copy of the summary PIS. If the patient would like to enter the trial, with the patient's verbal consent, a preliminary telephone screening will take place in order to check for basic inclusion and exclusion criteria. This telephone screening will follow a prepared transcript and will include only basic questions including: age, self-reported height and weight (to determine BMI), date of type 2 diabetes diagnoses and current drug therapies (to exclude patients with insulin controlled type 2 diabetes or NSAID requirements) and history of GI surgery or bleeding. Written consent will be obtained from the patient to contact their GP in order to gain further details on the patient's medical history, current medical therapies and to identify any medical reasons why the patient should not take part. At Southampton, patient's written consent will also be obtained to access the Hampshire Health Record (HHR) which contains patient information extracted from GPs and Hospital records. This information will be available to all clinical research staff at Southampton. A copy of the full PIS will be sent to the patient before the screening visit.

Patients who appear to meet eligibility criteria following their telephone screening and on review of their GP feedback, will be invited to attend a screening visit.

Patients will be informed of the nature of the study and given relevant information about the objectives of the research, benefits and possible adverse events, verbally and in writing. They will also be asked to participate in one of the three sub-groups of the mechanistic research part of the study. The methods and potential risks to which participants will be exposed to will be explained to them. Participants will have the opportunity to ask any questions and will be informed of their right to withdraw from the study at any time, without incurring any repercussions. Participants will be given as much time as is required to consider their participation in the study. Study procedures will be performed only once the participant has given full written informed consent and satisfies all the inclusion/exclusion criteria. It must be stated in the medical record that the subject is participating in the current trial.

After obtaining informed consent at the screening visit, subject's eligibility will be further assessed and documented by using a questionnaire with a list of inclusion and exclusion criteria, medical history (including all medications past and current) will be acquired and the following measurements will be performed: body weight, height, waist circumference, blood pressure, ECG, urine dipstick and pregnancy test, blood parameters (see 5.5.10) and female patients will be asked to report the last day of their menstrual period, the length of their cycle and the length of their menstruation (bleeding). This information will be used to ensure they are not pregnant and it will also help to monitor any changes in their menstrual cycle during the course of the study. Patient demographics will also be collected. At the screening visit, patients will be given the opportunity to consent for participation in one of the mechanistic sub-groups (1-3) of this study. Following informed consent, patients interested in consenting for participation in Sub-group 1 of the study will also be screened for suitability by checking the patient does not self-report to be vegetarian,

vegan, gluten or lactose intolerant (to be recorded in the CRF). They will also be checked for their fMRI suitability using these questionnaires:

(i) Metal check form - to ensure safety for MRI scanning, as may preclude entry into fMRI study
(ii) Handedness Inventory[53] - inability to use a right-handed button pad will preclude entry into fMRI study using Handedness.

5.2 Randomisation

After the screening visit, all eligible patients for the trial will be randomised into one of the two arms of the study via the InForm system (the eCRF database for the study) which will be programmed with a randomisation schedule provided by an independent statistician. This will protect against bias in the randomisation process as patients are allocated automatically. The randomisation will be stratified by site and two BMI groups, 30-40 and 40-50 kg/m^2. The subjects will be informed about their allocated treatment arm on visit 2.

Only the subject number and subject initials will be recorded in the case report form, and if the subjects name appears on any other document (e.g. pathologist report) it will be completely anonymised. The investigator will maintain a personal subject identification list (subject numbers with the corresponding subject names) to enable records to be identified.

5.3 Visit Schedule

A summary of all visits and procedures has been outlined below. A more detailed description of each visit and accompanying procedures can be found later in the protocol (5.4-5.6).

Table 2. Summary of study visit schedule

	Screening	Baseline		Treatment														Follow-up					
**Activities**	**V1**	**V2**	**V3**	**V4**	**T1**	**V5**	**V6**	**T2**	**V7**	**T3**	**V8**	**T4**	**V9**	**T5**	**V10**	**V11**	**T6**	**V12**	**T7**	**V13**	**T8**	**V14**	**V15**
Week/ Month/Day		- 4w ±7d	- 2w ±7d	- 0w ±3d	+ 5d± 3d	+ 10d ±3	+ 1m ±7d	+ 2m ±7d	+ 3m ±7d	+ 4.5m ±7d	+ 6m ±7d	+ 7.5m ±7d	+ 9m ±7d	+ 10.5m ±7d	+ 11.5m ±7d	+ 12m ±7d	+ 13.5m ±7d	+ 15m ±7d	+ 16.5m ±7d	+ 18m ±7d	+ 19.5m ±7d	+ 23m ±7d	+ 24m ±7d
Informed consent (5.1)	X																						
Inclusion & exclusion criteria (4.2, 4.3)	X																						
Demographics (5.1)	X																						
Medical history (including meds) (5.1)	X																						
Physical examination (5.5.4)	X																						
ECG (5.5.5)	X																						
Vital signs (5.5.7)	X	X	X	X		X	X		X		X		X		X	X		X		X		X	X
Body weight (5.5.8)	X	X	X	X		X	X		X		X		X		X	X		X		X		X	X
Height (5.5.8)	X																						
Waist circumference (5.5.9)	X	X	X	X		X	X		X		X		X		X	X		X		X		X	X
Routine blood tests (5.5.10)	X		X			X	X		X		X		X		X	X		X		X		X	X
Urine dipstick and female pregnancy test (5.5.6)	X																						
Changes in medical history/medication (5.5.4)		X	X	X		X	X		X		X		X		X	X		X		X		X	X
Randomisation (5.2)		X																					
Health Economic Questionnaires (5.5.12)			X			X	X		X		X				X							X	
Dietary counselling (5.5.3)		X		C																			
Dietitian follow up (5.5.3)							X		X				X			X		X		X			X
Urine albumin:creatinine ratio (5.5.11)			X			X					X				X							X	
Reporting of AEs (6)		X	X	X		X	X		X		X		X		X	X		X		X		X	X
DNA & RNA sampling (5.5.13)			X			x					x					x							x
Telephone counselling (5.5.3)					X			X		X		X		X			X		X		X		
Diabetologist/ Endocrinologist Review (5.5.1 and 5.5.2)		X		C			X		X				X			C		X		X			X

	Screening	Baseline		Treatment														Follow-up					
**Activities**	**V1**	**V2**	**V3**	**V4**	**T1**	**V5**	**V6**	**T2**	**V7**	**T3**	**V8**	**T4**	**V9**	**T5**	**V10**	**V11**	**T6**	**V12**	**T7**	**V13**	**T8**	**V14**	**V15**
Week/ Month/Day		- 4w ±7d	- 2w ±7d	- 0w ±3d	+ 5d± 3d	+ 10d ±3	+ 1m ±7d	+ 2m ±7d	+ 3m ±7d	+ 4.5m ±7d	+ 6m ±7d	+ 7.5m ±7d	+ 9m ±7d	+ 10.5m ±7d	+ 11.5m ±7d	+ 12m ±7d	+ 13.5m ±7d	+ 15m ±7d	+ 16.5m ±7d	+ 18m ±7d	+ 19.5m ±7d	+ 23m ±7d	+ 24m ±7d
Metabolomics (5.5.14)			X			X					X				X							X	
Bioelectrical Impedance (5.5.15)			X			X					X				X							X	
EndoBarrier Group Only																							
PPI and H. Pylori test (5.5.16)		X																					
Distribution of Proton Pump Inhibitors (5.5.2)		T																					
EndoBarrier Implant (5.5.2)				T																			
Preparation for Endobarrier removal (5.5.2)													T										
EndoBarrier removal (5.5.2)																T							
Biopsies during Implant and Explant (5.5.2)				T												T							
Gastroenterologist appointment (5.5.2)		T					T		T				T			T		T		T*			**T**
Sub-groups																							
Fixed/test meal and post-meal gut hormones and metabolites (Group 1 and 3) (5.6.4)			X			X					X				X								
Gut hormones and metabolites (Fasting only)(Group 1-3) (5.6.4)			X			X					X				X							X	
Food diaries (Groups 1-3) (5.6.2)			X			X					X				X							X	
Eating & Behaviour Questionnaires (Groups 1-3) (5.6.1)			X								X				X							X	
Appetite Visual Analogues Scales (Group 1-3) (5.6.3)			X			X					X				X							X	
Eating behaviour computerised tasks (Group 1 and 3) (5.6.5)			X								X				X							X	
Metal Check Form (Group 1) (5.1)	X																						
Handedness Questionnaire (Group 1) (5.1)	X																						
Additional pregnancy tests			F								F												

	Screening	Baseline		Treatment														Follow-up					
Activities	V1	V2	V3	V4	T1	V5	V6	T2	V7	T3	V8	T4	V9	T5	V10	V11	T6	V12	T7	V13	T8	V14	V15
Week/ Month/Day		- 4w ±7d	- 2w ±7d	- 0w ±3d	+ 5d± 3d	+ 10d ±3	+ 1m ±7d	+ 2m ±7d	+ 3m ±7d	+ 4.5m ±7d	+ 6m ±7d	+ 7.5m ±7d	+ 9m ±7d	+ 10.5m ±7d	+ 11.5m ±7d	+ 12m ±7d	+ 13.5m ±7d	+ 15m ±7d	+ 16.5m ±7d	+ 18m ±7d	+ 19.5m ±7d	+ 23m ±7d	+ 24m ±7d
DS-R disgust questionnaire (Group 1) (5.6.6)			X																				
Functional MRI (Group 1) (5.6.9)			X								X												
Insulin Clamps (Groups 2) (5.6.10)			X			X					X												
Cognitive assessment tasks (Group 1) (5.6.9)			X								X				X								
Food Preference / Taste Assessment(Group 3) (5.6.11)			X			X					X												
24hr Dietary Recall (Group 3) (5.6.11)			X			X					X				X							X	

X=performed in all patients unless otherwise stated

F=performed in Females only

C= performed in Control arm (Standard medical therapy) only

T= performed in Treatment arm (Endobarrier) only

**= optional (at request of the patient)*

5.4 Visit procedures

Visit 1 – Screening visit

The following will be performed:

- Informed consent (5.1)
- Inclusion and exclusion criteria (4.2, 4.3)
- Demographics (5.1)
- Medical history including diseases, disorders and medication (5.1)
- Physical examination (5.5.4)
- Body measurements including height, weight and waist circumference (5.5.8, 5.5.9)
- Vital signs (5.5.7)
- ECG (5.5.5)
- Routine haematology and biochemistry (5.5.10)
- Metal check form (sub-group 1 only) (5.1)
- Handedness questionnaire (sub-group 1 only) (5.1)
- Urine dipstick and female pregnancy test (females) (5.5.6)
- Registration on screening log (5.1)

Once all data including blood test results related to the screening have been obtained, the Investigator will review the subject's eligibility to continue in the trial. If the subject is eligible after this review, subject will be randomised into one of the two study arms (using InForm eCRF).

Visit 2

The following will be performed:

- Body measurements including weight and waist circumference (5.5.8, 5.5.9)
- Vital signs (5.5.7)
- Adverse events (6)
- Medical history/medication changes (5.5.4)
- Advised of randomisation into either the EndoBarrier (treatment) group or standard care (control) group (5.1)
- H. Pylori test (5.5.16)
- Dietary counselling with dietitian (see 5.5.3)
- Preparation with gastroenterologist for EndoBarrier Implant (Treatment arm only) (5.5.2) – including distribution of proton pump inhibitors.
- Diabetologist/Endocrinologist (5.5.1)

Visit 3, 5, 8, 10, and 14

The following will be performed:

- Body measurements including weight and waist circumference (5.5.8, 5.5.9)
- Vital signs (5.5.7)
- Routine chemistry and haematology (5.5.10)
- Urine albumin-creatinine ratio (5.5.11)
- Adverse events (6)
- Medical history/medication changes (5.5.4)
- Health economics questionnaires (EQ-5D-5L and service use) (5.5.4)
- Collection of blood for DNA & RNA (5.5.13)
- Metabolomics protocol (5.5.14)
- Bioelectrical impedance analysis (5.5.15)
- Functional MRI protocol except visit 5, 10 and 14 (sub-group 1) (5.6.8)
- Fasting gut hormones and metabolites (sub-group 1-3) (5.6.4)
- Post meal gut hormones and metabolites (sub-group 1 and 3) (5.6.4)
- Eating and behaviour questionnaires except visit 5 (sub-group 1-3) (5.6.1)
- Insulin clamp protocol except visit 10 and 14 (sub-group 2) (5.6.10)
- Taste and food preference protocol except visit 10 and 14 (sub-group 3) (5.6.11)
- Eating behaviour computerised tasks except visit 5 (sub-group 1 and 3) (5.6.5)
- Cognitive assessment tasks except visit 5 and 14 (sub-group 1) (5.6.9)
- Food diaries (sub-groups 1-3) (5.6.2)
- Appetite visual analogue scales (sub-groups 1-3) (5.6.3)
- DS-R Disgust questionnaire visit 3 only (sub-group 1) (5.6.6)
- 24hr Dietary Recall sub-group 3 only (5.6.11)

Visit 4

The following will be performed:

- Body measurements including weight and waist circumference (5.5.8, 5.5.9)
- Vital signs (5.5.7)
- Adverse events (6)
- Medical history/medication changes (5.5.4)
- Dietary counselling (control arm only) with dietitian (5.5.3)
- Diabetologist/Endocrinologist diabetes review (control arm only) with (5.5.1)
- EndoBarrier Implant (Treatment arm only) (5.5.2)
- Gastric and small bowel biopsies during EndoBarrier Implant (Treatment arm only) (5.5.2)

Visit 6, 7, 9, 11

The following will be performed:

- Body measurements including weight and waist circumference (5.5.8, 5.5.9)
- Vital signs (5.5.7)
- Routine chemistry and haematology (5.5.10)
- Adverse events (6)
- Medical history/medication changes (5.5.4)
- Dietitian follow up (5.5.3)
- Diabetologist/Endocrinologist diabetes review (5.5.1)
- Health Economics questionnaires (Visit 6 and 7 only) (5.5.4)
- Follow up appointment with gastroenterologist (Treatment arm only) (5.5.2)
- Preparation for EndoBarrier Removal (Visit 9 Treatment arm only) (5.5.2)
- EndoBarrier Removal (Visit 11 Treatment arm only) (5.5.2)
- Gastric and small bowel biopsies during EndoBarrier Explant (Treatment arm only) (5.5.2)

Visit 12, 13

The following will be performed:

- Body measurements including weight and waist circumference (5.5.8, 5.5.9)
- Vital signs (5.5.7)
- Routine chemistry and haematology (5.5.10)
- Adverse events (6)
- Medical history/medication changes (5.5.4)
- Dietitian follow up (5.5.3)
- Follow-up gastroenterologist (visit 13 on request only) (Treatment arm only) (5.5.2)
- Diabetologist/Endocrinologist diabetes review (5.5.1)

Visit 15 – End of Trial

The following will be performed:

- Body measurements including weight and waist circumference (5.5.8, 5.5.9)
- Vital signs (5.5.7)
- Routine chemistry and haematology (5.5.10)
- Adverse events (6)
- Medical history/medication changes (5.5.4)
- Dietitian follow up (focussing on relapse prevention) (5.5.3)
- Diabetologist/Endocrinologist diabetes review (5.5.1)

- Follow up gastroenterologist (treatment arm only)
- Discussion between patient and clinician to determine follow-up care plan and return to routine clinical care pathways.

Telephone counselling

The following will be performed during the telephone consultation at 5 days, 2, 4.5, 7.5, 10.5, 13.5, 16.5 and 19.5 months (T1-8):

- Well-being (changes in concomitant medication and illness)
- Motivation (compliance)
- Reminder of next study visit

Unscheduled visits due to adverse events (AE)

- If a subject is seen by an Investigator for an adverse event or if an Investigator is notified of an adverse event by a primary care giver or referring physician, an Adverse Event Case Report Form must be completed.

Differences between Mechanistic Study Groups

For subjects opting to participate in one of the 3 sub-groups of the study (visit 3, 5, 8, 10, and 14) they will have the following assessments, in addition to measurements of weight, waist, blood pressure, routine biochemistry and haematology, bio-electrical impedance analysis, adverse events and eating behaviour questionnaires:

Table 3. Summary of mechanistic study visit schedule

Group	Visit 3<br>-2 weeks	Visit 5<br>+10 days	Visit 8<br>+6 months	Visit 10<br>+11.5 months	Visit 14<br>+23 months
**1**<br>**MRI scans**	fMRI<br>Eating behaviour<br>Cognitive assess<br>Fasting & Post-meal hormones	Fasting & Post-meal hormones	fMRI<br>Eating behaviour<br>Cognitive assess<br>Fasting &Post-meal hormones	Eating behaviour<br>Cognitive assess<br>Fasting &Post-meal hormones	Eating behaviour<br><br>Fasting hormones
**2**<br>**Insulin clamp**	Insulin clamp<br>Fasting hormones	Insulin clamp<br>Fasting hormones	Insulin clamp<br>Fasting hormones	Fasting hormones	Fasting hormones
**3**<br>**Food Preference**	Taste and food preference assess<br>Eating behaviour<br>Fasting &Post-meal hormones	Taste and food preference assess<br>Fasting &Post-meal hormones	Taste and food preference assess<br>Eating behaviour<br>Fasting &Post-meal hormones	Eating behaviour<br>Fasting &Post-meal hormones	Eating behaviour<br><br>Fasting hormones

Summary of study visits for sub-group 1

1. fMRI brain scan – visits 3, 8
2. Eating behaviour computerised tests – visits 3, 8, 10, 14
3. Cognitive assessment tasks – visits 3, 8, 10
4. Eating and behaviour questionnaires – visits 3, 8, 10, 14
5. Appetite visual analogues scales (VAS) - visits 3, 5, 8, 10, 14
6. Test meal with measurement of pre and post-meal hormones and metabolites - visits 3, 5, 8, 10
7. Fasting gut hormones and metabolites – visit 14
8. Food diaries– visits 3, 5, 8, 10, 14

Summary of study visits for sub-group 2

1. Euglycaemic hyperinsulinaemic clamp – visits 3, 5, 8
2. During clamp: Eating and behaviour questionnaires – visits 3, 8, 10, 14
3. Appetite visual analogues scales (VAS) - visits 3, 5, 8, 10, 14
4. Measurement of fasting hormones and metabolites - visits 3, 5, 8, 10, 14
5. Food diaries - visit 3, 5, 8, 10, 14

Summary of study visits for sub-group 3

1. Sweet taste detection testing – visits 3, 5, 8
2. Consummatory taste reward testing- visits 3, 5, 8
3. Mixed meal tolerance test with measurement of post-meal hormones and metabolites - visits 3, 5, 8, 10
4. Eating behaviour computerised tasks – visits 3, 8, 10, 14
5. Eating and behaviour questionnaires – visits 3, 8, 10, 14

6. Appetite visual analogues scales (VAS) - visits 3, 5, 8, 10, 14
7. Measurement of fasting hormones and metabolites - visits 3, 5, 8, 10, 14
8. Food diaries visit 3, 5, 8, 10, 14
9. 24h dietary recall – visits 3, 5, 8, 10, 14

5.5 Description of procedures and measurements

5.5.1 Diabetologist/Endocrinologist Diabetes Review

The standard medical therapy arm will be carried out in accordance with the guidelines of the American Diabetes Association[52] (see figure 2 for summary algorithm). These guidelines have been chosen as they are applicable to an International audience and thus would adhere to the current best worldwide practice that would still be likely to be relevant when the results are published following study completion.

Diabetes reviews appointments with a Diabetologist/Endocrinologist will be performed with the control arm patients at visits 2, 4, 6, 7, 9, 11, 12, 13 and 15. Diabetes reviews appointments with a Diabetologist/Endocrinologist will be performed with the EndoBarrier arm patients at visits 2, 6, 7, 9, 11, 12, 13 and 15. Blood glucose concentrations obtained as part of study-scheduled routine blood tests will be used alongside self-measurements and self-reporting of blood glucose concentrations by patients or their GP's to adjust medication regimens. Home blood glucose meter readings will be indicated, as in routine clinical practice, when patients are clinically suspected or at risk of hypoglycaemic episodes (e.g. when commenced on sulphonylurea) or when glucose readings are required for further titration of therapy.

A summary of general recommendations for anti-hyperglycaemic therapy in type 2 diabetes patients have been outlined below (Figure 2) according to the American Diabetes Association (ADA) and the European Association for the Study of Diabetes (EASD)[54].

All female patients will also be asked to report the last date of their menstrual period, the length of their cycle and the length of their menstruation (bleeding) at screening visit, visit 2, 4, 6, 7, 9, 11, 12, 13 and 15. This information will be used to ensure they are not pregnant and it will also help to monitor any changes in their menstrual cycle during the course of the study.

Figure 2. Summary of general recommendations for anti-hyperglycaemic therapy in type 2 diabetes

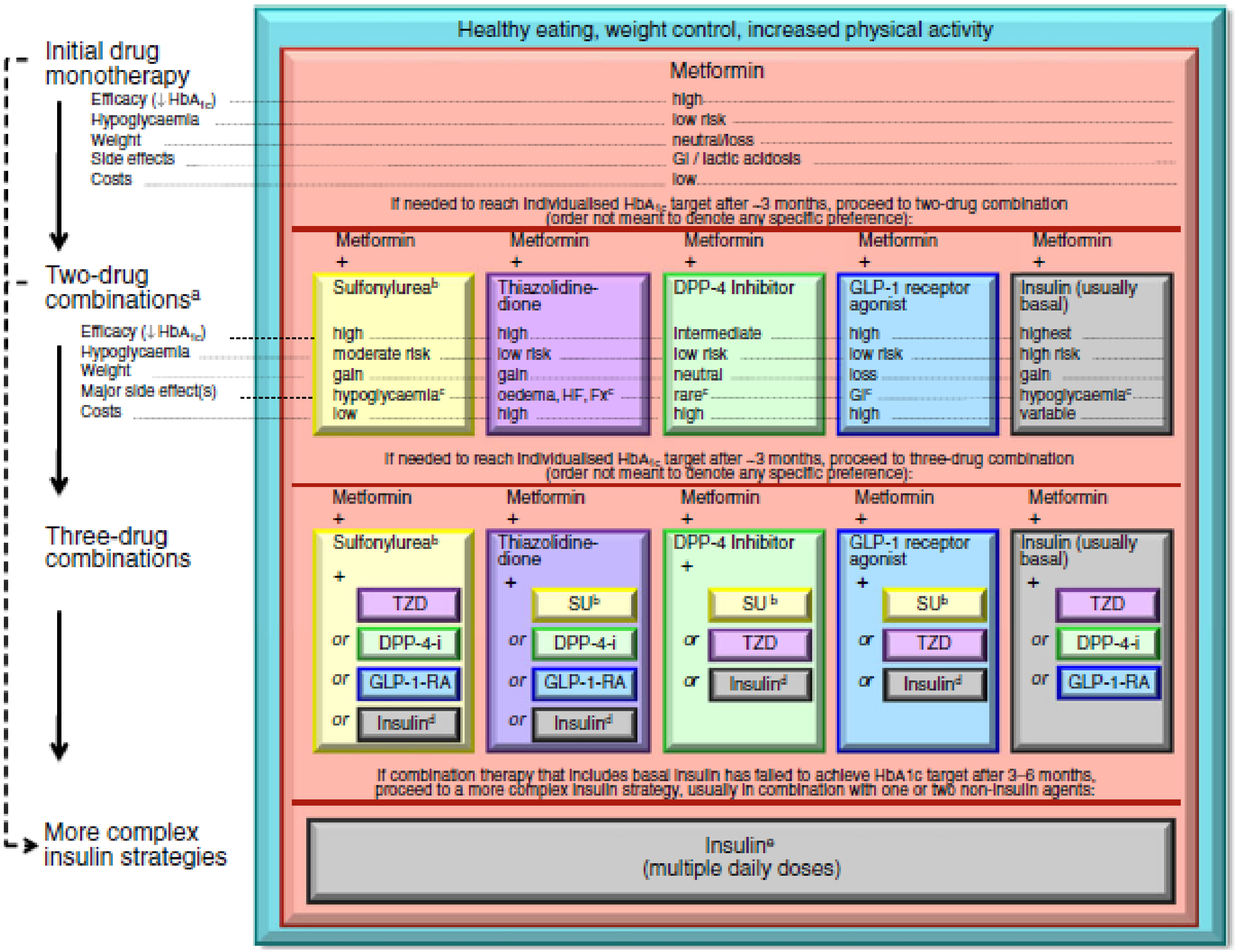

Antihyperglycemic therapy in type 2 diabetes: general recommendations. Moving from the top to the bottom of the figure, potential sequences of antihyperglycemic therapy. In most patients, begin with lifestyle changes; metformin monotherapy is added at, or soon after, diagnosis (unless there are explicit contraindications). If the HbA_{1c} target is not achieved after ~3 months, consider one of the five treatment options combined with metformin: a sulfonylurea, TZD, DPP-4 inhibitor, GLP-1 receptor agonist, or basal insulin. (The order in the chart is determined by historical introduction and route of administration and is not meant to denote any specific preference.) Choice is based on patient and drug characteristics, with the over-riding goal of improving glycemic control while minimizing side effects. Shared decision making with the patient may help in the selection of therapeutic options. The figure displays drugs commonly used both in the U.S. and/or Europe. Rapid-acting secretagogues (meglitinides) may be used in place of sulfonylureas. Other drugs not shown (α-glucosidase inhibitors, colesevelam, dopamine agonists, pramlintide) may be used where available in selected patients but have modest efficacy and/or limiting side effects. In patients intolerant of, or with contraindications for, metformin, select initial drug from other classes depicted and proceed accordingly. In this circumstance, while published trials are generally lacking, it is reasonable to consider three-drug combinations other than metformin. Insulin is likely to be more effective than most other agents as a third-line therapy, especially when HbA_{1c} is very high (e.g., ≥9.0%). The therapeutic regimen should include some basal insulin before moving to more complex insulin strategies. Dashed arrow line on the left-hand side of the figure denotes the option of a more rapid progression from a two-drug combination directly to multiple daily insulin doses, in those patients with severe hyperglycemia (e.g., HbA_{1c} ≥10.0–12.0%). DPP-4-i, DPP-4 inhibitor; Fx's, bone fractures; GI, gastrointestinal; GLP-1-RA, GLP-1 receptor agonist; HF, heart failure; SU, sulfonylurea. [a]Consider beginning at this stage in patients with very high HbA_{1c} (e.g., ≥9%). [b]Consider rapid-acting, non-sulfonylurea secretagogues (meglitinides) in patients with irregular meal schedules or who develop late postprandial hypoglycemia on sulfonylureas. [c]Additional potential adverse effects and risks, under "Disadvantages." [d]Usually a basal insulin (NPH, glargine, detemir) in combination with noninsulin agents. [e]Certain noninsulin agents may be continued with insulin. Consider beginning at this stage if patient presents with severe hyperglycemia (≥16.7–19.4 mmol/L [≥300–350 mg/dL]; HbA_{1c} ≥10.0–12.0%) with or without catabolic features (weight loss, ketosis, etc.).

5.5.2 EndoBarrier

The EndoBarrier Gastrointestinal Liner device received CE Mark for 12 months implant duration on 11 December 2009 and is a single use, minimally invasive device, used to achieve weight loss and improve Type 2 Diabetes status in subjects who are obese. The intent of the EndoBarrier Gastrointestinal Liner is to mimic portions of the standard Roux-en-Y bypass procedure. The device consists of 3 components: the implant, the delivery system, and the removal system. Each component will be provided sterile to the end user. A brief description of each component is presented below. At study visit 4, after eight hours fasting, subjects will arrive to the pre-assessment unit as part of the theatres at St. Mary's Hospital or Southampton Hospital. Subjects will be instructed to take an proton pump inhibitor (PPIs) (omeprazole 40 mg BID) 3 days prior to their device implant procedure and will continue with the medication throughout the study and for 2 weeks after the explant. PPIs (supplied by the research team) will be distributed at study visit 2. A subject implant card will be provided to the subjects prior to discharge, which describes the implant, identifies who to call in the case of an emergency, and what symptoms to look for post-implant.

Implant

The implant component of the system is comprised of a nitinol anchor, which is used to reversibly affix the device to the wall of the duodenum and an impermeable fluoropolymer EndoBarrier Gastrointestinal Liner extending approximately 2 feet into the small bowel. The anchor materials are commonly used in implants and have a long history of biocompatibility.

The implant is open at both ends to allow for passage of chyme (a semi-fluid mixture of partially digested food) from the stomach to the lower jejunum. The anchor portion of the device is located in the duodenal bulb, proximal to the bile duct. The EndoBarrier Gastrointestinal Liner portion resides in the duodenum and a portion of the jejunum. While the chyme passes through the inside of the EndoBarrier Gastrointestinal Liner, the bile and pancreatic enzymes pass outside the Liner. The bile and pancreatic enzymes will mix with the chyme at the end of the EndoBarrier Gastrointestinal Liner.

During implant of the EndoBarrier device eight gastric and small bowel biopsies will be taken using a standard biopsy forceps prior to the device being inserted. Four biopsies will be used for histology - placed in a specimen pot containing formalin and sent to St Mary's pathology lab for analysis. Four biopsies will be used for RNA extraction to perform genome-wide expression analysis – stored in Allprotect Tissue Reagent for future analysis.

Delivery

The implant is delivered on a custom catheter fabricated from materials with a long history of biocompatibility (medical grade PEBAX and high density polyethylene tubing and PTFE-coated stainless steel wire). The sterile catheter is approximately 3 meters long to facilitate delivery of the implant through the mouth into the jejunum. The catheter is designed to be sufficiently flexible to track through the intestine without kinking. Implantation and removal of the EndoBarrier device requires fluoroscopic x-ray guidance to determine the position of the device. Videos and photos of

the fluoroscopy images are recorded to help the investigators make treatment decisions and may be passed on to the study sponsor. The images will not contain any patient identifiable data but will be labelled with initials and study participation number only. Mean fluoroscopic x-ray time for insertion is 7 minutes (range 1-20 minutes). The constraint (maximum effective dose) for the whole procedure (implant and explant) is 9 mSv which is equivalent to 3.3 years natural background radiation in the UK (2.7mSv/year background) and results in a cancer risk of 1 in 2200 for a healthy 40 year old (5% per Sv risk factor).

Removal

The EndoBarrier Gastrointestinal Liner will be removed at study visit 11 with a custom grasper that passes through the working channel of a standard gastroscope. The gastroscope which is fitted with a foreign body retrieval hood is used to locate the implant. The grasper is passed through the working channel of the gastroscope and used to grab the polypropylene tether located on the proximal portion of the anchor. The proximal end of the anchor is collapsed by pulling on the tether and the anchor is then pulled into the foreign body hood. Once the collapsed anchor is in the hood, the implant is removed by withdrawing the gastroscope through subject's mouth.

During explant of the EndoBarrier device eight gastric and small bowel biopsies will be taken using a standard biopsy forceps after the device has been removed. Four biopsies will be used for histology - placed in a specimen pot containing formalin and sent to St Mary's pathology lab for analysis. Four biopsies will be used for RNA extraction to perform genome-wide expression analysis – stored in Allprotect Tissue Reagent for future analysis.
All subjects will be seeing a gastroenterologist or equivalent supervised healthcare specialist at study visit 2, 6, 7, 9, 11, 12and 15 (visit 13 on request) to discuss their well-being and any questions regarding their EndoBarrier implant.

Diabetic Medications Titration Schema

Patients in the EndoBarrier treatment arm will also be seen by the diabetologist/endocrinologist for diabetes review at visits (2, 6, 7, 9, 11, 12, 13 and 15).

Titration for Sulfonylureas: Subjects must have their dose of sulfonylureas reduced by 50% at the time of implant to avoid potential hypoglycaemic episodes. If a hypoglycaemic episode is experienced their sulfonylureas must be reduced by 50% again or discontinued if the subject is on the lowest dose.

Titration for Metformin: Dosages of metformin will remain unchanged throughout the trial unless the sulfonylureas have already been discontinued and a subject's fasting blood glucose is documented under 5.6 mmol/L on 3 consecutive days. If the fasting blood glucose is under 5.6 mmol/l, metformin can be discontinued. If the fasting glucose is below 3.5 mmol/L then metformin should be discontinued. See section 5.1 regarding blood glucose measurements.

If hypoglycaemic episodes are experienced, it is at the Investigator's discretion to modify or discontinue metformin.

Rescue Therapy: The decision whether a subject has inadequate glycaemic control during the course of the study, sulfonylurea will be increased or insulin will be started should be made at the Investigator's discretion. This will be performed by giving a glucose meter to patients. These subjects are to receive appropriate intervention at the Investigator's discretion. After removal, subjects are to continue their diabetic medication regimen. It is recommended that subjects have their dose of sulfonylureas increased or re-introduced if previously withdrawn during the course of the study if HbA1c > 7.0%. It is also recommended if, on three successive self-tests, fasting blood sugar is > 8 mmol/l and/or post prandial glucose is > 12 mmol/l. If titrating medications back to study baseline levels is deemed to be ineffective, subsequent interventions are at the Investigator's discretion. Any changes in medication will be discussed with the Imperial College Diabetologist coordinator.

5.5.3 Dietetic Input

Dietary counselling on diet and physical activity

At visit 2, all patients history and current eating behaviour will be assessed using the following information: anthropometry, biochemistry, co-morbidities, medication, activity level, eating habits including eating disorders and emotional eating, previous diets, lifestyle including smoking, drug and alcohol misuse, weight history, psychiatric history, family history of obesity, diabetes, mental illness or eating disorders, family history and available support network, work status, readiness and motivation for change, and understanding of both study arms.

At visit 2 and 4 (visit 4 control arm only) patients will receive diet and physical activity counselling by a qualified dietitian according to local standards. The dietary counselling programme intends to provide each subject with lifestyle and behavioural modification information, and good eating practices. On visit 2, all subjects in the Treatment arm will receive written information on how their diet will change after EndoBarrier insertion and written information will be provided for the liquid diet (see below) pre- and post- EndoBarrier insertion. They will also receive specialised advice for eating with their EndoBarrier after the liquid diet phase. Subjects in the control arm will be required to follow the same liquid diet as the EndoBarrier arm in order to standardise the groups. At visit 2, the control arm will also receive written information on how to follow the liquid diet. On visit 4 the control arm will receive information on how to follow a low calorie diet after the liquid diet ends (see below).

Liquid diet

In order to treat both study arms (control and treatment arm) equally all subjects will follow a liquid diet (based on milk, flavoured milk or milk replacements, water, low-sugar squashes, smooth/ clear soups, vegetable juices, tea or coffee without sugar, or unsweetened puree fruit juice) during the 7 days before and 13 days (± 3 days) after the intervention visit (visit 4).

Subjects in the EndoBarrier arm will also receive information on food exclusions, tips for eating and drinking, use of vitamin and mineral supplements, and long-term dietary considerations.

After the liquid diet, subjects in both study arms are advised to follow a low-calorie diet which is based on the guidelines below.

Low-calorie diet

Subjects are recommended to consume 600 calories less every day, depending on their age, gender, activity levels and body weight. Guidelines for daily amounts are between 1200 and 1500 calories for women and between 1500 and 1800 calories for men.

According to local standards (Diabetes UK), subjects are advised to eat regularly every day (5 times/ day), to control their portion sizes, and intake of carbohydrates/ starchy foods, to increase their intake of low glycaemic index (GI) and high protein foods, as well as vegetables, and to reduce their intake of foods high in fat, sugar, and alcohol.

Exercise advice

Patients in both the EndoBarrier and control arms will be given the same advice to include more physical activity in their daily routine for example walk more every day, climb the stairs instead of taking the lift or escalators, walk to the next bus stop, walk to the shops, ride an exercise bike while watching TV. They will also be encouraged to do more activity in their leisure time such as going for a walk, cycle ride or swim with friends or family. They will be asked to start with short periods of low-intensity exercise and increase the intensity and duration slowly. Their goal will be to include 150 minutes (2 ½ hours) a week of moderate intensity and 75 minutes a week of vigorous intensity aerobic activity and muscle strengthening activities more than 2 days a week. Changes in physical activity level will be monitored using the International Physical Activity Questionnaire (IPAQ)[55] at Dietetic follow up appointments. Tools such as pedometers will be used as a motivator to help individuals increasing their exercise. Any exercise will be adjusted to individual needs and activity levels.

Dietitian Follow up

Patients in both the EndoBarrier and control arm will be seen for review by the study dietitian at visits 6, 7, 9, 11, 12, 13, and 15. Motivation, dietary compliance and the average daily level of physical activity will be recorded.

Telephone counselling

In between visits at 5 days, 2, 4.5, 7.5, 10.5, 13.5, 16.5 and 19.5 months, (T1-8) subjects will be contacted by the dietitian or another researcher to assess patient's well-being and motivation on the trial. They will be also reminded to attend their next study visit.

5.5.4 Medical history and physical examination

A medical history will be performed at the screening visit to record illnesses, disorders and medications. This information needs to be updated on all follow-up visits.

Physical examination will be performed at the Screening visit (study visit 1) according to local procedure. During this visit the doctor will perform a cardiology, respiratory and gastrointestinal examination on the patient. They may go on to examine other appropriate systems if clinically indicated.

Any abnormal, clinical significant findings must be recorded in the eCRF.

Any changes in subsequent visits as compared to the screening visit which fulfils the criteria of an AE must be recorded as an AE. Any changes in concomitant illness will be recorded as changes in medical history. Any changes in medications will be recorded in the eCRF.

5.5.5 ECG

An ECG will be performed at the screening visit. The ECG will be interpreted, signed and dated by the investigator before study visit 2.

The interpretation must follow the categories:
- Normal
- Abnormal, not clinically significant
- Abnormal, clinically significant

If the ECG is abnormal and clinically significant, then the subject will be sent for further investigations and if necessary treated prior to study entry. If the abnormality cannot be treated then they will not be able to enter the study.

5.5.6 Urine dipstick and pregnancy test

Urine dipstick tests will be performed at the screening visit to determine pathological changes (e.g. protein in urine and any evidence of infection) in patient's urine. Any abnormal, clinical significant findings must be recorded in the eCRF.

Female urine pregnancy tests will be performed for females of childbearing potential at screening and at any time during the trial if a menstrual period is missed.

Female patients of child bearing potential who take part in the EndoBarrier trial will have already been consented to confirm they have no intentions to get pregnant during the duration of the study period. The use of adequate contraceptive methods will also be reinforced to each individual at screening and during each study visit. In the unlikely event that a patient's pregnancy test comes

back positive the patient will be adequately advised regarding the pros and cons of removing the device at that time. Endoscopy is safe during pregnancy but we have no data regarding the safety profile of the EndoBarrier device or indeed its metabolic effects during pregnancy. We would therefore advise that in general, if a patient falls pregnant during the study, that the device is removed when it is safe to do so.

Pregnancy tests will not be required for females who have undergone a hysterectomy or bilateral tubal ligation, or for females above the age of 50, who have been without menstrual period for at least one year. Pregnancy tests will also be performed for all females in sub-group 1 prior to fMRI (visits 3 and 8).

5.5.7 Vital Signs

Pulse should be recorded at all visits after resting for five minutes in a sitting position. Systolic and diastolic blood pressure will be measured preferably in sitting position at all visits.

5.5.8 Body weight and height

Weight should be measured at all visits. The same pair of scales should preferably be used throughout the trial.

Height without shoes will be recorded at the screening visit.
BMI will be calculated as follows: BMI = weight (kg)/height^2 (m^2).

5.5.9 Waist circumference

Waist circumference will be determined at all visits. Three consecutive measurements will be performed at each visit and recorded in the eCRF. The waist circumference will be measured in the horizontal plane to the nearest 0.5 cm using non-stretchable measuring tape.

5.5.10 Routine biochemistry and haematology

Blood samples will be obtained at all visits (except visit 2 and 4) after an 8 hour fast and processed by the local laboratory at Imperial College London NHS Trust or at University Hospital Southampton NHS Foundation Trust using standard methods' for routine tests. Patients can take their regular morning medications but are asked that they do not take any of their diabetes medications/oral hypoglycaemics on the morning of their study visit. Patients should bring their regular medication along to their study visits to be further advised by the doctor. See table below for a detailed description of blood collection at each visit:

Table 4. Summary of blood tests at each study visit

Blood test	V1	V3	V5	V6	V7	V8	V9	V10	V11	V12	V13	V14	V15
Haematology (Full blood count)	x	x	x	x	x	x	x	x	x	x	x	x	x
Routine Biochemistry (including Urea and Electrolytes)	x	x	x	x	x	x	x	x	x	x	x	x	x
Liver Function test	x	x	x	x	x	x	x	x	x	x	x	x	x
Fasting glucose	x	x	x	x	x	x	x	x	x	x	x	x	x
Creatinine	x	x	x	x	x	x	x	x	x	x	x	x	x
HbA1C	x		x		x	x	x	x		x	x		x
Lipids (cholesterol, HDL, LDL, triglycerides)	x	x	x	x	x	x	x	x		x	x	x	x
C-Peptide	x												
Insulin	x	x	x			x		x				x	
Vitamin D	x					x		x					
Iron studies	x					x		x					
Vitamin B12	x					x		x					
Serum Folate	x					x		x					
Free Thyroxine	x							x					x
TSH	x							x					x
Cortisol (Sub-group 1 only)		x				x							
Oestradiol (Sub-group 1 only)		x				x							
Progesterone (Sub-group 1 only)		x				x							
LH (Sub-group 1 only)		x				x							
FSH (Sub-group 1 only)		x				x							

5.5.11 Urine albumin:creatinine ratio

Early morning urine samples will be collected at visit 3, 5, 8, 10 and 14 to quantity the urinary albumin:creatinine ratio of each patient which is a useful measure of renal function used in diabetic renal disease.

5.5.12 Health Economics Questionnaires

Patients in both treatment arms will be asked to complete the health economics questionnaires at visits 3, 5, 7, 8, 10 and 14. These comprise the EQ-5D-5L questionnaire (http://www.euroqol.org/eq-5d-products/how-to-obtain-eq-5d.html) to assess health-related quality of life, and a bespoke questionnaire designed to collect information about patients' use of health and social care resources (for costing purposes).

The resource use questionnaire will be adapted from existing instruments including the CONSTRUCT (Williams J G, Russell I, Cohen D, et al. Comparison of infliximab and ciclosporin in steroid resistant ulcerative colitis: a trial CONSTRUCT, NIHR Health Technology Assessment

programme,http://www.hta.ac.uk/project/1737.asp; http://www.dirum.org/instruments/details/27) and Chernak (Chernyak et al. BMC Health Services Research 2012, 12:303, http://www.biomedcentral.com/1472-6963/12/303) questionnaires and will ask patients to specify what services they have used since the previous assessment. The resource use questionnaire will include:

- Medications for diabetes, weight loss, blood pressure, lipid control and cardiovascular disease;
- Primary care consultations (with GP, nurse or other healthcare professional);
- Hospital outpatient clinic visits (by specialty);
- A&E attendances (admitted/not-admitted);
- Inpatient stays and procedures;
- Investigations
- And use of other related community health and social services (e.g. chiropody).

Only services funded by the NHS or local authority personal social service departments will be included, as the health economic analysis will follow an 'NHS and PSS' perspective as recommended by NICE (Guide to the methods of technology appraisal 2013, http://publications.nice.org.uk/pmg9). Costs for private health and social care, out-of-pocket expenditure by patients, and 'indirect costs' per patient time will not be included.

In addition to the patient questionnaire, information will be collected in the CRF in order to cost the EndoBarrier intervention and the diet and exercise intervention. Information will be collected from hospital information systems and case notes, including:

- Routine assessments required before implantation and removal of the EndoBarrier;
- Disposables and staff time for the insertion and removal procedures, including day case and overnight stay if required; This information will adapted from other instruments (Resource Time Sheet page 194 of the HTA report for the MINuET trial: http://www.journalslibrary.nihr.ac.uk/hta/volume-10/issue-40);
- Treatment of any adverse events related to the procedures (additional inpatient stays, clinic visits and re-admissions);
- Dietitian time to deliver the diet and physical activity counselling and for telephone follow-up;
- Routine hospital follow-up and diabetes care;
- Hospital treatment for cardiovascular events or other complications of diabetes.

Costs related only to conduct of the trial (the outcome and mechanism assessments) will not be included, as these would not be required for routine implementation of the intervention. The cost of lifestyle coaching for both groups will be included because although this input should be similar for both groups, it is possible that attendance may differ.

5.5.13 DNA and RNA sampling

Blood (15 ml) will be taken at visit 3, 5, 8, 10 and 14 with specific consent to extract DNA and RNA for examination of genetic markers which predict weight loss, genetic abnormalities causing or contributing to obesity and insulin resistance (including array comparative genomic hybridization for copy number variations, epigenetic analysis, whole genome or exome sequencing, DNA sequencing of candidate genes (such as MC4R, POMC, leptin receptor, SIM1); and polymorphisms or mutations associated with obesity, diabetes mellitus, PCOS, fat distribution and body composition (using PCR based SNP analysis)[56-58]. RNA will be extracted from blood to perform genome-wide expression analysis.

5.5.14 Metabolomics

Plasma, urine and faecal samples for metabolomics will be collected at visit 3, 5, 8 and 10 and 14.

Pre-labelled urine and faeces collection kits described below will be given to patients on all visits prior to collection.

Urine collection

At hospital: Following an overnight fast, patient will be asked to give a urine sample by using a specific urine container. Samples will aliquoted immediately and stored at -80 °C until analysis.

Faeces collection

Subjects are instructed to void in a disposable bedpan, to collect into the given sterile faecal container, to fill to 2/3 and to double bag the sample. If the sample is collected at home and subsequently brought to the hospital, it needs to be kept in the fridge until transporting to the hospital (should be less than 24 hours). Samples will be stored at - 80 °C until analysis including DNA extraction. Extraction will be performed as per manufacture instruction.

Blood sample collection and plasma extraction

Whole blood samples (1-1.2ml plasma in total (400-450ul for NMR and 600-750ul for MS). Blood will be collected via venepuncture into 6ml sodium heparinized vacutainers and centrifuged within 30 min of collection. Plasma should be transferred into eppendorf tubes and stored at -80 °C until analysis.

All samples will be analysed using Mass spectroscopy and NMR spectroscopy.

5.5.15 Bio-electrical impedance analysis

As well as baseline anthropometric measurements of height, weight, waist and hip circumference, patients will also have their percentage body fat determined by bio-electrical impedance analysis (at visit 3, 5, 8, 10 and 14). This is a painless, safe procedure to measure total body fat involves lying

on a bed, having two sticky pads placed on a hand and foot, and lying still for 1 minute, or standing on a metal platform for 1 minute so that the body's electrical resistance can be measured.

5.5.16 PPI and H. Pylori test

During study visit 2, patients will be prescribed PPIs (omeprazole 40mg BID) and will be instructed to commence this drug 3 days prior to their device implant procedure and will continue with the medication throughout the study and for 2 weeks after the explant. The patient will be provided with a clinical trial medication form for the PPI prescription.

All patients randomised into the EndoBarrier arm must be tested for the presence of H. Pylori during study visit 2. It is important that patients are not taking any PPI's, antibiotics or bismuth preparations when this test is performed. Therefore all patients attending study visit 2 will be reminded over telephone to stop their PPI's, antibiotics or bismuth preparations at least two weeks prior to study visit 2 to avoid false negative results when having their test that day. Histamine blockers (H2 antagonists) do not appear to affect the accuracy of the urea breath test and can be offered as an alternative.

Those patients testing positive for H. Pylori will be offered eradication therapy for 1 week following the standard triple therapy guidelines published in the British national formulary (BNF). They will then be retested 4 weeks later (study visit 2 will be repeated) with a repeat test to confirm eradication before continuing with the trial. Once the patient is confirmed to be H. Pylori negative they can restart their PPI which will have been distributed at study visit 2.

Patients already using PPIs and with no history of H. Pylori will remain on their PPI medication and more will be prescribed upon request on visit 2.

5.6 Sub-group specific procedures and measurements

5.6.1 Eating and Behaviour Questionnaires (sub-groups 1-3)

At Visits 3, 8, 10, 14 patients will complete eating and behavioural questionnaires (duration 60 minutes) to assess eating behaviour and attitudes and personality measures related to reward sensitivity and mood. If they do not manage to complete all the questionnaires in the time available they will be able to complete them at home using pen and paper or over the internet.

1. EPIC food frequency Questionnaire[59]
2. Dutch Eating Behaviour Questionnaire (DEBQ)[60] – to measure restraint, emotional and external influences on eating behaviour
3. Eating Disorder Examination Questionnaire (EDE–Q)[61] – to screen for existing eating disorder and binge eating
4. Behavioural Inhibition and Activation System (BIS / BAS) scales[62] – to measure punishment and reward sensitivity
5. Beck Depression Inventory (BDI–II)[63] – to measure to measure levels of depression and anxiety
6. Barratt Impulsivity Scale and UPPS-P[64,65] – to measure impulsivity

7. Fagerström Nicotine Dependence Scale [66]- to assess smoking behaviour
8. Three Factor Eating Questionnaire (TFEQ)[67] - to measure restraint, disinhibition and hunger 9. Positive and Negative Affect Schedule (PANAS)[68,69] – to measure tendency to experience positive and negative affect
10. International Physical Activity Questionnaire (IPAQ)[55] - to measure physical activity
11. Yale Food Addiction Scale[70] – to measure food addiction
12. Alcohol Use Disorders Identification Test (AUDIT) [71] - to assess alcohol use and alcohol-related consequences.
13. Power of Food[72] - to assess the psychological influence of the food environment
14. Pittsburgh Sleep Quality Index (PSQI)[73] - to measure quality and duration of sleep
15. Dumping Syndrome - Arts and Sigstad questionnaires[11,74,75] - to assess dumping symptoms after eating e.g. nausea, abdominal discomfort
16. Binge Eating Scale[76]- to assess binge eating severity
17. Hospital Anxiety and Depression Scale (HADS)[77] - to assess symptoms of anxiety and depression
18. Short-Form 36 Health Survey Questionnaire (SF36)[78] - to assess quality of life

5.6.2 Food diaries (sub-groups 1-3)

Around each visit participants will be asked to keep a food diary for up to three days in order to determine their dietary intake.

5.6.3 Visual analogue Scales (sub-groups 1-3)

Visual analogue scales will be used to assess subjective feelings including hunger, nausea, fullness, sleepiness, stress, anxiety, volume of food that can be eaten and food palatability at intervals throughout the study visits (3, 5, 8, 10 and 14). These will be given during fixed/test meal sampling periods.

5.6.4 Gut hormones and metabolites (sub-groups 1-3)

In addition to the routine biochemistry and haematology, venous blood samples will be taken at intervals by venepuncture through a cannula placed in the antecubital fossa. No more than 110 mls will be taken for these on each visit. Serial plasma levels of glucose and other metabolites, bile acids, glucose, insulin, leptin, gut hormones (including ghrelin, GLP-1, PYY), adipocytokines and markers of insulin resistance, and inflammation will be measured. Assays will be performed by the Dept. of Chemical Pathology at Imperial College Healthcare NHS Trust and by in-house assays, outside contracts and commercial kits for radio-immunoassay and ELISA. For sub-groups 1 – 3 these will be measured when fasted during study visits 3, 5, 8, 10 and 14. For sub-groups 1 and 3 these will also be measured after a fixed/test meal at visit 3, 5, 8 and 10 (except for visit 14). A fixed meal will be a fixed amount of food e.g. (Fortisip compact®, Nutricia, Wiltshire, UK; 250 mL, 600 kcal, 24.0 g protein, 74.2 g carbohydrates, and 23.2 g fat). A test meal will be a commercially available food

product served to excess that the patient will be instructed to eat until they feel comfortably full. For sub-group 2, only fasted measurements will be taken.

5.6.5 Eating behaviour computerised tasks (sub-groups 1 and 3 only)

At visits 3, 8, 10 and 14, subjects in Groups 1 and 3 will perform several neuropsychological tasks to assess traits related to eating behaviour (duration 1.5 hours):

1. Delay discounting task: to assess impulsivity in which subjects choose between different sizes of hypothetical meal or money from a computer screen at different times in the future or probability[79-83]

2. Food financial valuation and/or progressive ratio tasks: to assess the financial and reward value placed on food by seeing the amount of money subjects are prepared to spend on various food items (Dagher A., personal communication), and the number of times subjects will press a button in order to receive a small quantity of sweets[12].

3. Food choice task: to asses liking and wanting for different foods[84].

5.6.6 Disgust sensitivity Questionnaire (sub-group 1 only)

On visit 3 only, the DS-R questionnaire will be used to measure disgust sensitivity which alters brain responses to images[85,86].

5.6.7 Additional pregnancy test (sub-group 1 only)

All females with have a urinary pregnancy test at each MRI scanning visit (Visits 3 and 8). If positive they will be excluded.

5.6.8 Functional MRI (sub-group 1 only)

Twenty-four subjects in each group (Treatment and Control) will have fMRI scans to examine between brain function related to food reward and addictive behaviours at baseline and early after intervention, and these will be correlated psychological questionnaires and computerised tasks, and test meals.

The study visits will last up to 6-8 hours (Visit 3 and 8), and 3 hours (Visits 5, 10 and 14). Subjects will attend the NIHR/Wellcome Imperial Clinical Research Facility and Clinical Imaging Facility at the Hammersmith Hospital in the morning or at lunchtime.

The paradigms and psychological/cognitive tests used in the fMRI protocol have already been approved in a study looking at the longitudinal effects of dietary interventions, bariatric surgery and

drugs in obesity (10/H0707/60, Functional magnetic resonance of brain food reward in obesity and its treatment).

Subjects will be advised to avoid alcohol and strenuous exercise the day before and day of each study visit, and will be asked to fast from the evening before the visit if a morning scan/visit, or from breakfast for an afternoon scan/visit.

Subjects in sub-group 1 will have brain magnetic resonance imaging (MRI) scans duration 60-90 minutes. None of the MR techniques to be used employs ionizing radiation or intravenous contrast agents. Scanning will be performed on the 3.0 Tesla MR scanner in the Clinical Imaging Facility at the Hammersmith Hospital, Imperial College London. Subjects lie supine in the scanner with their head placed in a padded head coil for support. While in the scanner volunteers will have access to a buzzer to sound an alarm, and will be able to hear and respond to instructions from the scanning console. While in the scanner subjects view a mirror reflecting a computer screen mounted above or behind the head coil. Subjects can respond to instructions using a keypad or joystick held in their hand. Subjects can rate their hunger and mood at various times while in the scanner using this device.

The following anatomical brain scans will be collected at one or more of the visits:

(i) Anatomical T1 and T2-weighted MR scans to provide structural data on which to overlay the functional data.

(ii) Diffusion tensor imaging to examine white matter tracts.

The following resting state functional brain scans will be collected at one or more of the visits:

(iii) Arterial spin labelling to measure resting regional blood flow[87,88]. A radiofrequency (RF) pulse is applied to the neck so as to ‘magnetically-label’ blood in vessels that send blood to the brain. Subsequent MR scanning of the brain is therefore able to detect blood flow changes as the labelled blood circulates.

(iv) Resting state BOLD functional MRI to measure regional connectivity in activity in different brain regions at rest[89].

The following task-related functional MRI scans will be collected at each visit:

(v) Food pictures: subjects view a variety of different pictures (e.g. food, household objects, animals, blurred pictures as a baseline) and rate how 'appealing' the pictures are using the keypad[11,90].

(vi) Monetary incentive delay (MID) or food incentive delay task: a game in which subjects need to press a button during a specific time window when given a cue on the computer screen in order to win hypothetical monetary or food prizes of differing amounts to assess reward responsivity and ventral striatum function [91,92].

(viii) Impulsivity Task: to assess effects on response inhibitory control mediated in the prefrontal cortex-striatum e.g. Go-NoGo task[81,93,94]. The task contrasts brain activation during responses to infrequent no-go signals (e.g. 'do not press' button when viewing one image) compared to an implicit go baseline (e.g. 'do press' button when viewing a different image).

(ix) Control cognitive tasks: subjects undertake simple tests (e.g. viewing a 4Hz flashing checkerboard, pressing a button, reading, listening, speaking, recalling, thinking about words or numbers, looking at emotional pictures on a screen)

Some of these tasks (e.g. MID and Go/NoGo tasks) will be practiced outside of the MRI scanner to habituate the participants to the experimental procedures. Subjects will have continuous heart or breathing rate and pulse oximetry monitoring while in the MR scanner.

5.6.9 Cognitive assessment tasks (sub-group 1 only)

At visits 3, 8 and 10, subjects in sub-group 1 will perform several cognitive tasks to assess traits related to addictive behaviours and which are known to be altered in obesity (duration 1 hours):

1. WTAR word reading list: to document intellectual and cognitive level[95]- visit 3 only.
2. Tests of cognitive function, e.g. memory, attention from the Cantab® battery (www.cantab.com/cantab-tests.asp).

5.6.10 Insulin clamp (sub-group 2 only)

On visit 3, 5, and 8, patients will undergo a euglycaemic hyperinsulinaemic clamp with stable isotope infusion to determine overall insulin and compartment-specific insulin sensitivity (liver, muscle and adipose depot)

On the days prior to the clamp procedure patients' glucose-lowering medications will be adjusted by a research nurse or a clinician in order to avoid their interference with the clamp measurements. Patients will attend the research facility either the day prior to the study visit and stay overnight for an insulin sliding scale or they will attend the research facility in the morning after an overnight fast depending on their capillary glucose readings on the days before the clamp. Patients will be instructed to consume a standardised meal or meal replacement the evening prior to their study visit. A venous catheter will be inserted into a vein of each arm on the study morning. The first cannula will be used for infusions and the other for blood sampling. If blood glucose levels are higher than 6 mmol/l on arrival a variable rate insulin infusion will be started to attain a stable glucose level (4.0 -6.0 mmol/l) prior to commencement of the hyperinsulinaemic euglycaemic clamp. A primed continuous infusion of [6, 6-$^{2}H_2$] glucose, a stable isotope tracer, will be started and maintained for 7 hours. Two hours later a two-stage hyperinsulinaemic-euglycaemic clamp procedure will be started and continued for 5 hours. During stage 1 of the clamp procedure, in which hepatic insulin resistance is assessed, insulin will be infused at a low dose (depending on patient's weight/body surface area) for 2 hours. During stage 2 of the clamp procedure, in which peripheral insulin

resistance is assessed, insulin will be increased to a higher dose (depending on patient's weight/body surface area) for 3 hours. Euglycaemia will be maintained by infusing 20% dextrose at a variable rate. Blood samples will be taken every 5 minutes to measure blood glucose concentration and the dextrose infusion will be adjusted accordingly. The exogenous glucose infusion will be enriched with 6,6 $^2H_2$ glucose to prevent a fall in plasma tracer enrichment and underestimation of endogenous glucose production rate.

Blood samples will be obtained before the start of the tracer infusions, every 10 min during the final 30 min of the basal period and stages 1 and 2 of the clamp procedure and every 30 minutes between these periods to determine glucose enrichment and concentration, free fatty acid, insulin, c-peptide, glucagon, gut hormones, bile acids and metabolite concentrations. At the same time points participants will be asked to complete appetite visual analogue scales.

At the end of the study, participants will be fed and glucose infusion continued for a further 20 minutes to prevent hypoglycaemia. Depending on the patient's blood glucose control on arrival, the maximum duration of the whole visit will be up to 9 hours and the maximum amount of venesected blood will be 250 mls.

Blood samples will be centrifuged and the separated plasma kept in a - 20°C or - 80°C freezer. The isotopic enrichment of plasma glucose will be determined by gas chromatography mass spectrometry (GCMS) at the Wolfson Centre for Translational Research, Postgraduate Medical School, University of Surrey.

The stable labelled isotope tracers [6,6 $^2H_2$] is not a drug, but a naturally occurring metabolite which has been labelled with a stable, i.e. non-radioactive label. Stable isotope tracers are widely used in metabolic research by groups throughout the UK and worldwide. All labelled isotope tracers are ordered from Cambridge Isotopes Ltd through their UK suppliers CK gases Ltd. They are prepared as sterile solutions suitable for intravenous use by the Pharmacy Production Unit at Guys & St. Thomas' NHS Trust to ensure they are safe for the participants. The products are supplied with the appropriate certificate of analysis and MSDS. We have used the same manufacturer to be sure of the quality of the products and the supporting documentation.

5.6.11 Taste and food preference assessment (sub-group 3 only)

The total duration of these visits will be up to 7 hours (Visit 3, 5, 8, 10) and 5 hours (14). Patients will attend the research facility after an overnight fast. The participants' food diaries will be collected and they will be asked to complete VAS rating their appetite. A trained dietitian/nutritionist will perform a detailed 24 hour dietary recall assessment and the participants will be asked to complete the EPIC study food frequency questionnaire[59]. The food diaries, 24 hour recall and the EPIC questionnaire will be used to quantify total caloric intake and macronutrient composition.

Sweet taste detection testing

This will be performed at visits 3, 5, 8. Seven ascending sucrose concentrations in solution will used to determine sweet detection thresholds. These will made up by drawing a specific volume of sucrose and adding it to distilled water to produce 7 sucrose concentrations (0.0021M, 0.00526M, 0.0125M, 0.05M, 0.10M, 0.3M). All solutions and rinses will be prepared fresh on the day and presented at room temperature. Concentrations will be tested in 8 blocks with each block consisting of 7 sucrose and seven water stimuli. Sucrose and water stimuli will be presented in random order without replacement. Thus, each of the 7 sucrose concentrations will be presented once within a block. Using this method of constant stimuli[96] stimuli are presented randomly, eliminating expectation or habituation. The subjects will be given a period of 5 seconds to sample the stimulus in the mouth. Subjects will then spit the sample in a container and will be given another 5 seconds to indicate whether the stimulus was water or not. Each stimulus will be followed by a thorough 10 second water rinse which will be expelled before the next stimulus is offered. After 4 blocks, the assessment will be interrupted with a 10 minutes rest period.

Consummatory taste reward

This will be performed at visits 3, 5, 8. Five ascending fat and sucrose solutions/ice-cream will be used to test responses in intensity ratings and hedonic reward. These will be made up fresh on the day of testing and 20mls of each will be presented randomly to the participants in 3 trials (total 15 stimuli). Participants will be asked to put the solutions into their mouths, swirl it around and then to spit it out into a bucket – the "sip and spit" technique. While the solution is being swirled around in the participants' mouths they will be asked to rate its intensity and pleasantness using visual analogue scales (generalised labelled magnitude intensity and hedonic scales and the "just about right" scale). After each cup of sucrose solution, a water cup will also be given for the participant to swirl and spit in order to cleanse their mouths for the next tastant.

6 ADVERSE EVENTS (AE)

An AE is any untoward medical occurrence in a patient or clinical trial subject administered a medicinal product and which does not necessarily have a causal relationship with this treatment. An AE can therefore be any unfavourable and unintended sign (including an abnormal laboratory finding), symptom, or disease temporally associated with the use of the trial device/procedure, whether or not considered related to the treatment.

All adverse events that occur during this study will be recorded on the adverse event case report forms.

6.1 Adverse Event description

For the purposes of the study, AEs will be followed up according to local practice until the event has stabilised or resolved, or the Follow-up Visit, whichever is the sooner. SAEs will be recorded throughout the study.

6.2 Severity of Adverse Events

Mild: Awareness of event but easily tolerated
Moderate: Discomfort enough to cause some interference with usual activity
Severe: Inability to carry out usual activity

6.3 Causality of Adverse Events

Unrelated: No evidence of any causal relationship

Unlikely: There is little evidence to suggest there is a causal relationship (e.g. the event did not occur within a reasonable time after device implant). There is another reasonable explanation for the event (e.g. the patient’s clinical condition, other concomitant treatment).

Possible: There is some evidence to suggest a causal relationship (e.g. because the event occurs within a reasonable time after device implant). However, the influence of other factors may have contributed to the event (e.g. the patient’s clinical condition, other concomitant treatments).

Probable: There is evidence to suggest a causal relationship and the influence of other factors is unlikely.

Definite: There is clear evidence to suggest a causal relationship and other possible contributing factors can be ruled out.

6.4 Adverse Event Relationship Definition in the Control Group and Sub-Group 1-3

Each Investigator will categorize the types of events as follows:

1 = Definitely related to treatment: Any event that is associated with the treatment by timing and physiology, and was caused or contributed to by the treatment.

2 = Possibly related to treatment: Any event that is possibly associated with the treatment timing and physiology, and may have been caused or contributed to by the treatment.

3 = Not related to treatment: Any event that, although temporally associated, was not attributable to the treatment itself.

4 = Pre-existing or independent condition: Any event, although temporally associated, that was attributable to a pre-existing or independent condition.

5 = Unknown: The relationship of the event to the device, or procedure is unknown.

6 = Other: Any event that cannot be classified in any of the above categories.

6.5 Abnormal Laboratory Test Results

All clinically important abnormal laboratory test results occurring during the study will be recorded as adverse events. The clinically important abnormal laboratory tests will be repeated at appropriate intervals until they return either to baseline or to a level deemed acceptable by the investigator and the clinical monitor, or until a diagnosis that explains them is made.

6.6 Anticipated Adverse Events

<u>Control Group</u>

Anticipated adverse events in the control group will depend on the particular approved medication introduced for improved glycaemic control and weight loss, under diabetes management guidelines (Figure 2). Known common side effects (taken from the British National Formulary) may include the following:

1. Sulphonylureas

Hypoglycaemia
Nausea
Vomiting
Constipation
Diarrhoea

2. Thiazolidinediones

Weight gain
Nausea
Vomiting
Oedema
Anaemia

Headache
Visual disturbance
Dizziness
Arthralgia
Hypoaesthesia (reduced touch sensation)
Haematuria
Impotence

3. DPP-4 inhibitors

Nausea
Vomiting
Oedema
Upper respiratory tract infection
Nasopharyngitis
Pain
Osteoarthritis
Headache
Tremor
Asthenia (weakness)
Dizziness

4. GLP-1 receptor agonists

Nausea
Vomiting
Abdominal pain and distension
Diarrhoea or loose stool
Discomfort or reaction at injection site
Anorexia
Hypoglycaemia
Gastro-oesophageal reflux disease
Headache
Dizziness
Asthenia (weakness)
Increased sweating

5. Insulin

Hypoglycaemia
Discomfort or reaction at injection site
Weight gain
Lipodystrophy at injection site

6. Metformin

Nausea
Abdominal pain
Diarrhoea (usually transient)
Anorexia
Taste disturbance

Other anticipated adverse events in the control group can include hypoglycaemia, peptic ulcer or gallstones due to a change in food intake and weight loss.

Other anticipated adverse events

If the patient participates in any of the 3 sub-groups for the mechanistic tests, adverse effects may include discomfort during cannula insertion, bruising at the cannula insertion sites, but this should disappear within a few days after the study visit. Infection around the cannula site is very rare but readily treatable with oral antibiotics in the unlikely event of occurrence. During the insulin clamp tests, hypoglycaemia is very rare and the personnel performing the clamp procedure have been trained to avoid it and treat it appropriately should it occur.

Maximal venesection blood volumes for each sub-group are outlined below. The blood volumes taken over the 24 month time frame should not be associated with impaired iron status but routine haematology blood samples taken at visits 1-15 will be monitored for signs of compromised iron status and treated with iron if deemed appropriate.

EndoBarrier only (no sub-group) = 490ml (355ml year 1, 135ml year 2)
Sub-group 1 fMRI = 695ml (540ml year 1, 155ml year 2)
Sub-group 2 clamps = 1130ml (975ml year 1, 155ml year 2)
Sub-group 3 taste = 695mls (540ml year 1, 155ml year 2)

6.7 MRI safety and protection of patients

The MRI scanner contains a very strong magnet. Therefore, individuals may not be able to have the MRI if they have any type of metal implanted in their body, for example, any pacing device (such as a heart pacer), any metal in their eyes, or certain types of heart valves or brain aneurysm clips. Magnetic resonance imaging metal check list will be completed at each visit to ensure compatibility with MRI scanning.

There is not much room inside the MRI scanner. Some people may be uncomfortable if they do not like to be in close spaces (“claustrophobia”). This is therefore an exclusion criterion for recruitment to the MRI study. If subjects experience discomfort within the scanner despite the measures taken to ensure patient comfort, the patient may request immediate cessation of the procedure with withdrawal from the scanner by ringing the patient alarm bell.

The MRI produces a “hammering noise” but subjects wear earplugs and headphones to prevent discomfort or damage to hearing.
At fMRI study visits, anatomical brain scans will be collected. It should be noted that these scans cannot be viewed as a comprehensive health screening procedure. However, vary rarely, unexpected information can be detected which may warrant further investigation. In this event, a report will be sent to the patient's GP, who will arrange further tests and coordinate further care. Significant structural anatomical abnormalities will also preclude the subject from further

participation in the study. The anatomical brain MR scans will be reported by a Consultant Neuroradiologist at Imperial College Healthcare NHS Trust.

6.8 Definition of Device-Related Adverse Events

Device-related: any adverse event for which a causal relationship between the device and the event is a reasonable possibility (section 6.9).

The likelihood that the event is device related will be categorized as listed, the device-related "cause" will be categorised as follows:

- **Device Failure or Malfunction:** a device has failed if it is used in accordance with the Instructions for Use, but does not perform or function according to Instructions for Use and negatively impacts the treatment by preventing treatment as intended. Such a failure does not necessarily result in significant adverse outcome.

- **Unanticipated Adverse Device Effect (UADE):** Any serious adverse effect on health or safety or any life-threatening problem or death caused by, or associated with a device, if that effect, problem, or death was not previously identified in nature, severity, or degree of incidence in the investigational plan or application (including a supplementary plan or application) or prior medical literature, or any other unanticipated serious problem associated with a device that relates to the rights, safety, or welfare of subjects. This event needs to be entered into the eCRF SAE pages within 24 hours of the site's knowledge.

6.9 Adverse Event Relationship Definition in EndoBarrier Group

Each Investigator will categorize the types of events as follows:
1 = Definitely related to device: Any event that is associated with the device by timing and physiology, and was caused or contributed to by the device.

2 = Possibly related to device: Any event that is possibly associated with the device timing and physiology, and may have been caused or contributed to by the device.

3 = Definitely related to implant/explant endoscopic procedure: Any event that is associated with the implant/explant by timing or physiology, and was caused or contributed to by the implant/explant endoscopic procedure.

4 = Possibly related to implant/explant endoscopic procedure: Any event that is associated with the implant by timing or physiology, and may have been caused or contributed to by the implant/explant endoscopic procedure.

5 = Not related to device, implant or explant procedure: Any event that, although temporally associated (e.g., occurs during or at some time after implant/explant), was not attributable to the device itself, or to general endoscopic procedures.

6 = Pre-existing or independent condition: Any event, although temporally associated, that was attributable to a pre-existing or independent condition.

7 = Unknown: The relationship of the event to the device, or procedure is unknown.

8 = Other: Any event that cannot be classified in any of the above categories.

6.10 Adverse Device Effect (ADE)

An Adverse Device Effect (ADE) is any untoward and unintended response to a medical device. This definition includes any event resulting from insufficiencies or inadequacies in the instructions for use or the deployment of the device. This definition includes any event that is a result of a user error.

<u>Endobarrier group</u>

1. During the implant procedure, the anticipated adverse events may include:

Dizziness
Fever
Gastroenteritis
Headache
Hyper/hypotension
Liver chemistry changes
Pancreatic chemistry changes

2. During the implant period, the anticipated adverse events may include:

Vitamin and Mineral Deficiency
Dehydration
Constipation
Bloating
Diarrhea
Infection
Flatulence
GERD
Esophagitis
Pseudopolyps
Nausea/vomiting
GI pain/cramping
Peptic ulcer disease
Duodenitis
Local inflammatory tissue reaction
Flank/Back pain
Alopecia
Dizziness

Fever
Gastroenteritis
Headache
Hyper/hypotension
Liver chemistry changes
Pancreatic chemistry changes

3. During and after the explant, the anticipated adverse events may include:

Alopecia
Dizziness
Fever
Gastroenteritis
Headache
Hyper/hypotension
Liver chemistry changes
Pancreatic chemistry changes

All of these potential risks are treatable and should not result in a life-threatening situation if a subject is treated promptly. To minimize the chances of a life-threatening situation, each Investigator will discuss all of the signs and symptoms that each subject should watch for. Additionally, each subject will be sent home with a card that will also describe the signs and symptoms to watch for. If they experience any of the signs and symptoms described by their Investigator, they should be instructed to call them immediately, and he will instruct them if they should come to the hospital for treatment.

Insertion and removal of the EndoBarrier device is performed under fluoroscopic x-ray guidance. Each Investigator will be trained in the implantation and removal of the EndoBarrier Gastrointestinal Liner to minimise the exposure time to the x-rays. Mean x-ray exposure time in a previous EndoBarrier research study was 7 minutes for insertion (range 1-20 minutes) and 1.5 minutes for removal (range 0-15 minutes). The constraint (maximum effective dose) for the whole procedure (implant and explant) is 9 mSv which is equivalent to 3.3 years natural background radiation in the UK (2.7mSv/year background) and results in a cancer risk of 1 in 2200 for a healthy 40 year old (5% per Sv risk factor).

6.11 Serious Adverse Events (SAE)

An SAE is defined as any event that

- Results in death;
- Is life-threatening*;
- Requires hospitalisation or prolongation of existing inpatient's hospitalisation** (not applicable to planned EndoBarrier implant and explant procedures both requiring hospitalisation);
- Results in persistent or significant disability or incapacity;
- Is a congenital abnormality or birth defect;

* "Life-threatening" in the definition of "serious" refers to an event in which the subject was at risk of death at the time of the event; it does not refer to an event which hypothetically might have caused death if it were more severe.
** "Hospitalisation" means any unexpected admission to a hospital department. It does not usually apply to scheduled admissions that were planned before study inclusion or visits to casualty (without admission).

Medical judgement should be exercised in deciding whether an adverse event/reaction is serious in other situations. Important adverse events/reactions that are not immediately life-threatening, or do not result in death or hospitalisation but may jeopardise a subject, or may require intervention to prevent one of the other outcomes listed in the definition above should also be considered serious.

6.12 Serious Adverse Device Effect (SADE)

A Serious Adverse Device Effect (SADE) is an adverse device effect that has resulted in any of the consequences characteristic of a serious adverse event or that might have led to any of these consequences if suitable action had not been taken or intervention had not been made or if circumstances had been less opportune.

6.12.1 Anticipated Serious Adverse Device Effect (ASADE)

A serious adverse device effect which by its nature, incidence, severity or outcome has been previously identified in the risk analysis report or Clinical Investigation Brochure.

1. During the implant procedure, ASADE's may include:

GI Tract Laceration
Oropharyngeal Perforation
Oesophageal Perforation
Gastric Perforation
Bowel Perforation
Bleeding
Aspiration
Infection
DVT
Cholecystitis/Cholelithiasis
Pancreatitis with or without bile duct blockage

2. During the implant period, ASADE's may include:

Small Bowel Obstruction
Implant Migration
Bezoar
Erosion
Bleeding

Hypoglycaemia
Hyperglycaemia
Nausea/vomiting
Gastric or Bowel Perforation
Cholecystitis/ Cholelithiasis
Liver abscess (1% incidence)
Pancreatitis with or without bile duct blockage

3. During and after the explant, ASADE's may include may include:

GI Tract Laceration
Oropharyngeal Perforation
Esophageal Perforation
Gastric Perforation
Bowel Perforation
Bleeding
Aspiration
Adynamic Ileus
Infection
Cholecystitis/Cholelithiasis
Pancreatitis with or without bile duct blockage

6.12.2 Unanticipated Serious Adverse Device Effect (USADE)

Serious adverse device effect which by its nature, incidence, severity or outcome has not been identified in the current version of the risk analysis report or Clinical Investigation Brochure.

6.12.3 Serious Public Health Threat

Any event type which results in imminent risk of death, serious deterioration in state of health, or serious illness that requires prompt remedial action. This would include:

a. events that are of significant and unexpected nature such that they become alarming as a potential public health hazard

b. the possibility of multiple deaths occurring at short intervals."

6.13 Reporting of SAEs

Rapid reporting of all SAEs, occurring during the study or within 30 days following the completion of the study by the subject, must be performed as detailed in SAE reporting instructions. An SAE form should be completed and emailed to the CI for all SAEs within 24 hours of the sites knowledge. The CI or delegate will inform the sponsor of all SAEs within 24 hours of receiving notice of them. Additional information should be sent to the CI and sponsor within 5 days if the reaction has not resolved at the time of reporting. All unexpected SAEs should be recorded on the annual safety reports that are sent to REC on the anniversary of the date a favourable opinion for the study was given.

The Sponsor shall inform the device manufacturer (GI Dynamics) in writing about all unanticipated SAEs and incidents in writing within 24 hours. All anticipated SAEs shall be reported to GI Dynamics (GID) in writing within 7 calendar days.

The manufacturer is responsible to report any incidents and/ or unanticipated SAEs to the regulatory authority (MHRA) in compliance to the applicable regulation. GI Dynamics will perform the determination of Vigilance reporting based upon the requirements defined in the current version of MEDDEV 2 12.1.

Reporting timelines (device manufacturer to the MHRA) include:

1. 10 days for incidents and unanticipated SAEs indicating imminent risk of death, serious injury, or illness and require prompt medical reaction

2. 30 days for other reportable events

The sponsor shall supply GID all requested information to perform a comprehensive investigation of the incident and/ or SAE. This may include but is not limited to:

- Dates of occurrence
- Address and contact information of research site
- Patient identifier e.g. patient number, gender, age
- Full description of the event including symptoms, tests performed, treatments rendered, and final patient status at release
- Preconditions or concurrent medications that may have contributed to the event.
- Results of tests including but not limited to microbial isolates, endoscopic evaluations, CT scans, etc.
- Product return (EndoBarrier sleeve), using a return kit for package, for further analysis
- Swab samples of the device to be used in further analysis including DNA sequencing
- Hospitalization period
- Patient and physician compliance with the Instruction for use
- Other information that may be pertinent to the investigation

The request for the above information may occur as a request to complete GI Dynamic's form (F-070), email communication, or verbally.

The sponsor will retain the device in an unadulterated state, to allow for further sampling until provided a product disposition by GID. GID shall make the decision regarding need for device return within 20 calendar days.

The above information is forming part of the contract (amendment 1) with GID.

7 EARLY DISCONTINUATION OF THE STUDY OR INDIVIDUAL SUBJECTS

7.1 Early Discontinuation of the Study

The sponsor and/or Ethics Committee and/or government agencies may also request to stop enrolment of additional subjects for the study or an individual site for any reason. If the study enrolment is ended before the planned completion, subjects with a device implanted will be evaluated to determine if it is acceptable to leave the device in place and continue to follow the Schedule of Events.

In case of early discontinuation of the study, the follow-up visit (Visit 12 at 15 months) assessments should be performed for each subject, as far as possible. Subjects that have the device removed before Month 3 will return for the End of Treatment/Month 12 (visit 11). Subjects that have the device removed after Month 3 will return for the End of Treatment/Month 12 (visit 11), Month 15 (visit 12), Month 18 (visit 13) and Month 24 (visit 15) post explant follow-up visits.

7.2 Early Discontinuation of Individual Subjects

Subjects may discontinue study treatment (EndoBarrier) for the following reasons:

- At the request of the subject.
- If the investigator considers that a subject's health will be compromised due to adverse events or concomitant illness that develops after entering the study.

Any subjects who withdraw from the study that have the device implanted will have the device removed as soon as it can be arranged. Subjects that have the device removed before Month 3 will return for the End of Treatment/Months 12 (visit 11). Subjects that have the device removed after Month 3 will return for the End of Treatment/Month 12 (visit 11), Month 15 (visit 12), Month 18 (visit 13) and Month 24 (visit 15) post explant follow-up visits.

All subjects (including both control and device arm) who withdrew from the study early (either prior or after their treatment started – visit 4), and where no follow-up visit has been agreed or arranged as part of the previous protocol version (V4.1), will be contacted over the phone by the study team to ask for their permission (verbal consent documented by the study doctor using a specific form filed in the medical records) to perform a short telephone consultation at Month 24 (equivalent to visit 15). Data collected during this visit will include:

- HbA1 (provided through their last routine appointment at their GP)
- Body weight in kg
- Adverse events (section 6)
- Medical history/medication changes (section 5.5.4)

8 STATISTICAL ANALYSES

8.1 Sample Size and power considerations

The primary end-point of a 20% reduction in HbA1c has been chosen as the International Diabetes Federation produced in June 2011[97] new guidelines for the conduct of studies in diabetes using bariatric surgery or devices aiming to produce standardisation allowing comparison between studies. To date there are thus no published large patient group studies using this end-point, so using this new endpoint in a well-designed and conducted study will be of scientific value in itself.

Conservatively, it was estimated that 15% of patients in the control arm will achieve the target but believe this to be an overestimate. The Steno study [98] is the best quality randomised study (80 patients in each arm) into the effect of best medical therapy published to date and demonstrated over an average 7.8 years significant improvements in HbA1c amongst those having intensive medical therapy from 8.4+/- 1.6 to 7.7+/-1.2, but no change in HbA1c amongst those continuing with standard medical therapy. This study defines the very best that could realistically be achieved in the control arm, but expect there to be very little if any change in this group. The reporting of HbA1c as an outcome measure was not in accordance with the newly defined IDF criteria, but considering the small average reduction achieved in the Steno study, it will be assumed that a target of 15% of patients reaching the endpoint is a conservative estimate. Company data on the small number of patients who have reached a year with the device in place suggest that 40% will achieve this target.

According to our own experience with the device in the commercially sponsored study, up to 30% of patients in the treatment group may have the device removed early. Nevertheless other commercially sponsored (unpublished) studies of this device have achieved lower explant rates (J Tetreault – GI Dynamics). We have therefore diluted the treatment effect from 40% vs. 15% to 35% vs. 15% achieving the target of 20% reduction in HbA1c for treatment arm vs. standard arm. 73 patients per group will give 80% power to detect a significant effect. Adding 10% loss of follow-up increases the sample size to 80 per group.

The dilution was calculated starting from the assumption that 40% of patients with the device will reach the target (this estimate is based on company data based on diabetic patients in the same range of BMI as in the present proposal). If 30% of patients in the treatment group need to remove the device early but remain available for follow-up, in the worst case scenario, the proportion reaching the target is the same as in the control group, bringing the estimate for the treatment group to 32.5%. However most of them will keep the device for some time, having some benefit, so it is plausible to assume that the estimate is higher than 32.5%.

Dividing the main effect 15% vs. 40% in three parts we assume that in the 30% of patients with removal, for 1/3 the same effect will be achieved as in the control group (15% reaching the target), for 1/3 it will be increased (23% reach the target) and for 1/3 more increased (31% reach the target). Overall, this would give an estimate of 35% for the treatment group.

For that reason, 2 arms of 80 patients will be sufficient to ensure demonstration of a significant effect (if one exists) and very conservatively allows for explant rates of up to 30%, a higher, level of benefit in the control arm than is likely to be achieved, and drop-out rates of 10%.

Furthermore the landmark Steno study which in some ways may be considered similar to this study, that had in all likelihood a less effective intervention arm was sufficiently powered with 80 patients in each arm.

More than 80 subjects can be randomised at each study site in the event of patients dropping out post randomisation but prior to treatment start (visit 4). Randomising more patients is possible as the randomisation lists are designed to allow for additional patient recruitment. Also, randomisation

blocks provide sufficient randomisation such that any potential unbalancing would be minor and that final analysis would be unaffected. Any additional randomisation need to be discussed and agreed with the sponsor and the CI. All patients withdrawn post-visit 4, so after treatment start, are part of the initial drop out calculations as per protocol and will not be replaced. At no point, will the total number of patients starting treatment exceed 160 and the final analysis will not be affected.

8.2 Data Analysis

Patient characteristics will be summarized. Summaries of continuous variables will be presented as means and standard deviations if normally distributed, and as medians and inter-quartile ranges for skewed data, whilst categorical variables will be presented as frequencies and percentages. The difference between the two study groups in the proportion of patients achieving substantial improvement in the metabolic syndrome both at 12 and 18 months will be analysed using logistic regression adjusting for the stratification variables (BMI groups and sites). Analysis of secondary outcomes will be conducted using standard statistical procedures applicable to categorical or continuous data as appropriate. For missing values we will explore the pattern and the extent of missingness and we will carry out an appropriate form of multiple imputation if required. The analysis will be performed according to the intention to treat principle. All statistical tests will be two-tailed with a 5% significance level.

Metabolomics:
Metabolic datasets will be analysed using principal component analysis (PCA) and orthogonal partial least-squares analysis (O-PLS). The metabolic and microbial data will also be analysed in relation to response measurements such as BMI, gut hormone levels and etc. using O-PLS regression analysis and Bayesian approaches. A range of statistical methods will be optimised and applied to the data to identify weight loss and T2DM-associated microbiota and metabolites.

Health Economics:
The economic health analysis will be conducted following the NICE Reference Case, which includes the use of QALYs as the measure of health outcome, and adoption of an NHS and Personal Social Services perspective for costs (Guide to the methods of technology appraisal 2013, http://publications.nice.org.uk/pmg9). Both costs and QALYs will be discounted at the recommended rate of 3.5% per year.

Within trial analysis: EQ-5D health states will be scored using the UK social tariff to give a utility values at each time point (-2 weeks, 10 days, 1, 3, 6, 11.5, and 23 months). QALYs will be estimated for each patient using an area-under-the curve approach.

The cost of the EndoBarrier intervention, the lifestyle intervention, and other related health and social care will be estimated from resource use data. Unit costs for the included services will be obtained from standard national sources (BNF for drug prices, Department of Health Reference Costs for investigations, procedures and outpatient visits, PSSRU estimates for other primary and community health and social services). Total costs will be estimated for each patient over the 24-month trial period.

Patient-level cost and QALY estimates will be combined to estimate an Incremental Cost Effectiveness Ratio (ICER) for the Endobarrier device compared with standard medical therapy over the 24 month trial period. The analysis will combine multiple imputation to account for missing EQ-5D and resource use data with bootstrap regression to estimate mean cost and QALYs for the two patient groups. Missing data is often a particular problem for economic analysis, even in studies with good follow-up of primary endpoints, as area under the curve approach requires data from multiple time points. A bootstrap regression approach will be used to account for non-normal distributions of cost data, to adjust for baseline differences in utility or other patient characteristics, and to allow for correlations between costs and QALYs.

Cost-effectiveness modelling: A decision analytic model will be developed to estimate clinical outcomes, QALYs, and costs beyond 24 months. The time horizon for the modelling will be for the remainder of the patient's predicted lifetimes, as recommended in modelling guidelines[99,100] .

Before commencing this modelling exercise, we will review existing economic decision models for weight loss interventions for people with Type 2 diabetes. There are several validated diabetes models that have been compared and validated against external data[101]. These models take baseline information about patients' risk factors (demographic, behavioural and metabolic biomarkers), predict how these risk factors change over time, and hence simulate the incidence of diabetic complications, survival, quality adjusted survival, healthcare usage and costs. Most diabetes models rely on the UKPDS Risk Engine[102] to reflect the relationships between the risk factors. This series of equations is based on a multivariate analysis of data from the UKPDS clinical trial, adapted to reflect risk in a general T2DM population. For example, the UKPDS Outcomes Model[103] is a computer simulation model the lifetime benefits of diabetes-related interventions for use in economic evaluations. However, it is not clear that UKPDS-based models could adequately capture putative benefits from the EndoBarrier related to weight-loss or the remission of diabetes. The UKPDS risk engine does not include BMI as an independent predictor of diabetic complications; it does not include outcomes related to obesity but not to diabetes; and it cannot be used to model risk in patients in remission from diabetes.

A more recent model developed for the NICE Public Health guidance on preventing type 2 diabetes (Gillett et al. Prevention of type 2 diabetes: risk identification and interventions for individuals at high risk Economic Review and Modelling, ScHARR 2011, http://www.nice.org.uk/guidance/index.jsp?action=download&o=57046) may provide a more promising example of the type of model that we could use for the EndoBarrier evaluation. This uses an individual patient dataset from the Health Survey for England to characterise baseline profiles of risk factors within a representative population, and to estimate change in risk factors over time. Predictive algorithms from the QResearch primary care database (the QRisk2 and QDSCORE equations) are then used to predict the onset of diabetes and CVD. The model also allows for onset of other obesity-related diseases, including osteoarthritis and some cancers, and independent effects of BMI and diabetes on other-cause mortality and quality of life. It is then used to estimate the impact of five dietary interventions to prevent diabetes.

The review of this and other similar models will be used to identify possible model structures and sources of input parameters. The conceptual design of the model will be discussed and agreed

amongst the research team before programming commences. We anticipate that the model will take the form of an individual patient simulation, using either a discrete time or discrete event approach to simulate the onset of diabetes/obesity related complications, and hence QALYs and costs under alternative treatment strategies.

We will use data from the trial to provide estimates of the effect of the EndoBarrier compared with conventional management. In addition, we will review published systematic reviews and meta-analyses of bariatric surgery for similar a patient population. If recent evidence of sufficient relevance and quality is available, we will extend our model to include indirect comparisons with these other interventions. Other model parameters will be sourced from targeted literature reviews and routine data sources. The choice of software for the model will be made after specification of the conceptual design, but we have a similar model programmed in STATA, so this may provide a suitable platform for implementation. Before use, the model will be validated by an experienced health economist not involved in the development of the model. This will be done using a checklist developed by the Brunel Health Economics Research Group, which includes a range of suggestions for checking that a model is free from errors (verification) and that it is consistent with internal and external data (validation).

Probabilistic sensitivity analysis (PSA) will be used to estimate the impact of uncertainty over model parameters, and we will conduct a value of information (VOI) analysis to estimate the value of conducting further research. In addition, deterministic sensitivity analysis will be used to examine the impact of uncertainties over the model structure.

<u>Gut hormones and bile acid:</u>
These will be measured in the fasted and/or postprandial state for each patient and compared within and between the groups using parametric/non-parametric repeated measures statistical testing. Regressions will be performed with clinical outcomes (i.e. BMI, glucose control) to identify predictive markers and generate mechanistic hypotheses.

<u>Appetite food hedonics and brain reward systems:</u>
Comparison of brain activation during fMRI paradigms and outcomes from behavioural measures of food hedonics and questionnaires will be compared between groups using a 2x2 ANOVA design including group (control vs. Endobarrier) between subject factor, time (baseline vs. follow-up visit) within subject factor, and group x time interaction to identify differential effects between groups. For fMRI studies analysis will use region of interest analyses (e.g. for food pictures orbitofrontal cortex (OFC), amygdala, caudate, nucleus accumbens and anterior insula; for MID task nucleus accumbens, dorsal striatum and OFC; for Go-NoGo task pre-supplementary motor area). In addition whole brain analysis will be performed to compare groups using statistical thresholds of voxel-wise correction FDR $P<0.05$ and cluster-wise correction $Z>2.3$ $P<0.05$. In addition linear regression will be performed to measure the correlation of variables at baseline or during the intervention with primary outcomes at 1 year e.g. weight loss and decreases in HbA1c within each group, to generate predictive markers and generate mechanistic hypotheses.

<u>Food preference:</u>
Dietary energy intake, macronutrient composition, sweet taste detection thresholds, visual analogue taste ratings and eating behaviour psychological scores will be quantified for each

patient and compared within and between the groups at 3 time points using parametric/non-parametric repeated measures statistical testing. Regressions will be performed with clinical outcomes (i.e. BMI, glucose control) to identify predictive markers and generate mechanistic hypotheses.

Insulin Clamps:
Overall and tissue specific insulin sensitivity will be quantified for each patient and compared within and between the groups at 3 time points using parametric/non-parametric repeated measures statistical testing. Regressions will be performed with clinical outcomes (i.e. BMI, glucose control) to identify predictive markers and generate mechanistic hypotheses.

9 DATA MANAGEMENT

Data Management is the responsibility of the Imperial Clinical Trials Unit (ICTU) and the study Monitor. The subject and the biological material obtained from the subject will be identified by subject number, trial site and trial identification number. Appropriate measures such as encryption or deletion will be enforced to protect the identity of human subjects in all presentations and publications as required by local/ regional and national requirements.

Appropriate measures such as encryption of data files will be used to assure confidentiality of subject data when it’s transmitted over open networks.

The electronic laboratory data will be considered source data. In cases where laboratory data is transferred via non-secure electronic networks, data will be encrypted during transfer.

This study will capture and process data using The InForm electronic Case Report Form (eCRF) which will be built by ICTU. InForm is a fully validated high quality electronic data capture system, which has a full audit trail and controlled level of access. Data and reports will be extracted from the database throughout the study to monitor progress and training will be provided to all study staff on use of the database.

10 TREATMENT

10.1 Device Product Details

EndoBarrier Gastrointestinal Liners will be provided to each site according to the terms of the Investigator’s Agreement. Devices will be ordered and shipped through GI Dynamics. At the termination of the study, all unused devices not traceable to implantation or implantation attempt will be returned to GI Dynamics. Each Investigator must maintain records of receipt, use and disposition of the devices.

The following records will be kept:

- Records of devices received, including dates of receipt, quantity, lot number and the signature of the person in charge of device accountability.
- Records of device use, including the Subject ID, date of return (if appropriate), and lot number, quantity, reason for wastage or return, and the signature of the person returning the device.

11 REGULATORY, ETHICAL AND LEGAL ISSUES

11.1 Declaration of Helsinki

The investigator will ensure that this study is conducted in full conformity with the 1964 Declaration of Helsinki and all subsequent revisions.

11.2 Good Clinical Practice

The study will be conducted in accordance with the guidelines laid down by the International Conference on Harmonisation for Good Clinical Practice (ICH GCP E6 guidelines).

11.3 Independent Ethics Committee/Institutional Review Board Approval

11.3.1 Initial Approval

Prior to the enrolment of subjects, the REC must provide written approval of the conduct of the study at named sites, the protocol and any amendments, the Patient Information Sheet and Consent Form, any other written information that will be provided to the subjects, any advertisements that will be used and details of any subject compensation.

11.3.2 Approval of Amendments

Proposed amendments to the protocol and aforementioned documents must be submitted to the REC for approval as instructed by the Sponsor. Amendments requiring REC approval may be implemented only after a copy of the RECs approval letter has been obtained.

Amendments that are intended to eliminate an apparent immediate hazard to subjects may be implemented prior to receiving Sponsor or REC approval. However, in this case, approval must be obtained as soon as possible after implementation.

11.3.3 Annual Safety Reports and End of Trial Notification

The REC will be sent annual safety updates in order to facilitate their continuing review of the study (reference. ICH GCP E6 Section 3.1.4) and will also be informed about the end of the trial, within the required timelines.

11.4 Insurance

Imperial College London, as the Sponsor in the UK, provides indemnity and insurance for this study.

11.5 Informed Consent

The participation of a subject in this clinical trial is voluntary.

The investigator or a member of the research team will approach the patient to obtain informed consent.

The background of the proposed study, the procedure, the follow-up schedule and all potential benefits and risks will be carefully explained to each subject. The person obtaining the informed consent shall:

- Avoid any coercion or undue influence of subjects to participate
- Not waive or appear to waive subject’s legal rights
- Use language that is non-technical and understandable to the subject
- Clarify the subject that his/her data are confidential and are encoded with a subject ID number during the investigation
- Provide ample time for the subject to consider his/her participation
- Include dated signatures of the subject and of the clinical investigator
- Ask whether the subject has any questions about the study

After a subject has received and read the patient information sheet and agrees to participate in the study, the informed consent form approved by the Ethics Committee must be signed by the subject prior to any study specific tests being performed. It will also be signed by the person responsible for collecting the informed consent. The original will be kept in the subjects study research notes (source documents), a copy will be given to the subject and a copy kept in their hospital notes. The participation to take part in one of the 3 sub-groups in this study is optional. Patients will also be asked to give permission to be contacted after 4 years in order to provide new information on their body and blood measurements. Participants will also be asked to consent to being contacted about upcoming research studies in the event that they are not eligible to take part in this study.

11.6 Contact with General Practitioner

It is the investigator’s responsibility to inform the subject’s General Practitioner by letter that the subject is taking part in the study provided the subject has consented to this, and information to

this effect is included in the Patient Information Sheet and Informed Consent. A copy of the letter should be filed in the Subjects research record (source data). The General Practitioner will also be informed about any new medication prescribed at the study centre.

11.7 Subject Confidentiality

The investigator must ensure that the subject's privacy is maintained. On the CRF or other documents submitted to the Sponsors, subjects will be identified by a subject ID number only. Documents that are not submitted to the Sponsor (e.g., signed informed consent form) should be kept in a strictly confidential file by the investigator.

The investigator shall permit direct access to subjects' records and source document for the purposes of monitoring, auditing, or inspection by the Sponsor, authorised representatives of the Sponsor, Regulatory Authorities and RECs.

11.8 End of Trial

The trial will end after the last subject has been seen for their last study visit. All patients will be reviewed by a clinician at their last study visit in order to arrange return to appropriate routine clinical care pathways.

11.9 Study Documentation and Data Storage

The investigator must retain essential documents until notified by the Sponsor, and at least for ten years after study completion, as per the Sponsor's requirements. Subject files and other source data (including copies of protocols, CRFs, original reports of test results, correspondence, records of informed consent, and other documents pertaining to the conduct of the study) must be kept for the maximum period of time permitted by the institution. Documents should be stored in such a way that they can be accessed/data retrieved at a later date. Consideration should be given to security and environmental risks.

No study document will be destroyed without prior written agreement between the Sponsor and the investigator. Should the investigator wish to assign the study records to another party or move them to another location, written agreement must be obtained from the Sponsor.

12 ADMINISTRATIVE MATTERS

12.1 Source Data (CRF)

As a minimum requirement the following data must be source data verifiable in source documentation other than the eCRF:

- Existence of subject (subject identifier, subject number and date of birth)
- Confirmation of participation in the trial (subject identification number (ID), trial ID and signed and dated informed consent forms
- Diagnosis/ indication under investigation
- Visit dates
- Data from AEs, safety information form and pregnancy forms
- Relevant medical history, concomitant illness
- Reason for exclusion or withdrawal
- Body weight

The existence of each subject must be confirmed either via their medical records or a copy of the passport or ID card could be used.

12.2 Language

CRFs will be in English. Generic names for concomitant medications should be recorded in the CRF wherever possible. All written material to be used by subjects must use vocabulary that is clearly understood, and be in the language appropriate for the study site.

12.3 Data Collection

All data collected will be documented in the source document (CRF) and eCRF (InForm).

12.4 Electronic Recording of data

This study will capture and process data using The InForm electronic Case Report Form (eCRF) which will be built by ICTU. InForm is a fully validated high quality electronic data capture system, which has a full audit trail and controlled level of access. Data and reports will be extracted from the database throughout the study to monitor progress and training will be provided to all study staff on use of the database. The Investigator must ensure that the data is recorded in the eCRFs as soon as possible after the visit preferably within 3 working days.

12.5 Study Management Structure

12.5.1 Trial Steering Committee (TSC)

A Trial Steering committee (TSC) with an independent Chair will be appointed and will be responsible for overseeing the progress of the trial. A TSC Charter will be devised to list the roles

and responsibilities of the TSC members. TSC will be convened biannually either in person or by teleconference.

Two PPI representatives will sit on the Trial Steering Committee and will provide input from a patient perspective at trial meetings.

12.5.2 Trial Management Group

The Trial Management Group will be set up by the Chief Investigator (CI). TMG will convene on a monthly basis and will discuss on the recruitment, and other practical aspects of the trial. The TMG will include the CI, Project Manager, ICTU representative and PI at the Southampton site as well as other site staff when appropriate.

The day-to-day management of the trial will be co-ordinated through the Imperial Clinical Trials Unit via the Project Manager and the Chief Investigator.

12.5.3 Data Monitoring & Ethical Committee (DMEC)

An independent Data Monitoring and Ethical Committee (DMEC) will be set up to monitor progress, patient safety and any ethical issues involved in this trial. They will review trial progress, recruitment rates, event rates and safety data. A separate charter will be drawn up defining their exact remit and criteria for reporting to the trial steering committee. There will be 6-monthly meetings of the independent DMEC.

12.6 Monitoring

A monitoring plan will be devised based on risk analysis and described in detail in the monitoring manual. During the course of the trial the Monitor will visit the trial sites to ensure that the protocol is adhered to, that all issues have been recorded and to perform source data verification. The study will be monitored periodically by the Project Manager and/or a Clinical Trial Monitor within Imperial Clinical Trials Unit.

Initiation visits will be completed at all trial centres prior to the recruitment of participants, and will consist of review of protocol and trial documents, training with respect to trial procedures (informed consent, SAE reporting, inclusion and exclusion criteria), review of recruitment strategy, review of site facilities and equipment, essential document receipt (including NHS permission), collection and filing, and archiving and inspection. Copies of the trial specific procedure manuals and related documents will be given to the investigators. The approved version of the protocol should be followed at all times, and any significant protocol deviations will be documented in a Protocol Deviation Form and any significant deviations will be recorded on a Protocol Violation Form submitted to the study coordination centre and Sponsor as soon as possible. The investigators will allow the monitors to:

- inspect the site, the facilities, device management and materials used for the trial

- meet all members of the team involved in the trial, and ensure all staff working on the trial are experienced and appropriately trained, and have access to review all of the documents relevant to the trial
- have access to the electronic case record forms and source data
- discuss with the investigator and site staff trial progress and any issues on a regular basis

The monitor will ensure that:

- A percentage of records will be inspected for confirmation of existence, eligibility based on the results of the Risk Assessment
- 100 % of consent forms will be reviewed along with all SAE's.
- there is adherence to the protocol, including consistency with inclusion/exclusion criteria
- there is GCP and regulatory compliance
- trial documentation is complete and up to date (e.g. correct versions of documents being used, source data captured) and relevant documents are collected for the Trial Master File (TMF)
- the monitored eCRFs have been completed correctly and accurately, and all entries correspond to data captured in source documents

The Monitor must be given direct access to the source documents (original documents, data and records). Direct access includes permission to examine, analyse, verify and reproduce and record reports that are important to evaluation of the clinical trial.

All information dealt with during such visits will be treated as strictly confidential. At the end of the trial, close out visits will be performed by the monitor after the final participant visit has been completed and prior to database lock. During this visit the monitor will verify that all trial close out activities are completed – all queries resolved, missing data completed, monitoring completed, archiving arrangements in place, ISF completed and TMF documents collected, and end of trial notification. Each investigator will also be notified that an audit or inspection may be carried out - by the sponsor, sponsor's representatives or the host institution, or regulatory authorities - at any time, before, during or after the end of the trial. The investigator must allow the representatives of the audit or inspection team:

- to inspect the site, facilities and material used for the trial,
- to meet all members of his/her team involved in the trial,
- to have direct access to trial data and source documents, to consult all of the documents relevant to the trial. If an Investigator is informed of an impending audit or inspection, the trial coordination centre should be notified immediately.

12.7 Quality Control and Quality Assurance

Quality Control will be performed according to ICTU internal procedures. The study may be audited by a Quality Assurance representative of the Sponsor or the Quality Assurance manager within ICTU.

All necessary data and documents will be made available for inspection. The Quality Assurance Manager within ICTU will review all documents before submission to the REC and will perform a risk assessment of the study which will form the basis of the monitoring plan.

12.8 Disclosure of data and publication

Information concerning the study, patent applications, processes, scientific data or other pertinent information is confidential and remains the property of the Sponsor. The investigator may use this information for the purposes of the study only.

Verbal or written discussion of results prior to study completion and full reporting should only be undertaken with written consent from the Sponsor.

Therefore all information obtained as a result of the study will be regarded as CONFIDENTIAL, at least until appropriate analysis and review by the investigators is completed.

Permission from the Trial Steering Committee is necessary prior to disclosing any information relative to this study outside of the Steering Committee.

13 REFERENCES

1. CDC. Prevalence of overweight and obesity among adults with diagnosed diabetes--United States, 1988-1994 and 1999-2002. in *MMWR Morbidity and mortality weekly report*, Vol. 53 1066-1068 (2004).
2. Hoerger, T.J., Segel, J.E., Gregg, E.W. & Saaddine, J.B. Is glycemic control improving in U.S. adults? *Diabetes care* **31**, 81-86 (2008).
3. Sjostrom, L.*, et al.* Bariatric surgery and long-term cardiovascular events. *JAMA : the journal of the American Medical Association* **307**, 56-65 (2012).
4. Mingrone, G.*, et al.* Bariatric surgery versus conventional medical therapy for type 2 diabetes. *The New England journal of medicine* **366**, 1577-1585 (2012).
5. Schauer, P.R.*, et al.* Bariatric surgery versus intensive medical therapy in obese patients with diabetes. *The New England journal of medicine* **366**, 1567-1576 (2012).
6. Dixon, J.B., le Roux, C.W., Rubino, F. & Zimmet, P. Bariatric surgery for type 2 diabetes. *Lancet* **379**, 2300-2311 (2012).
7. le Roux, C.W.*, et al.* Gut hormone profiles following bariatric surgery favor an anorectic state, facilitate weight loss, and improve metabolic parameters. *Annals of surgery* **243**, 108-114 (2006).
8. Pournaras, D.J.*, et al.* The role of bile after Roux-en-Y gastric bypass in promoting weight loss and improving glycaemic control. *Endocrinology* **153**, 3613-3619 (2012).
9. Simonen, M.*, et al.* Conjugated bile acids associate with altered rates of glucose and lipid oxidation after Roux-en-Y gastric bypass. *Obesity surgery* **22**, 1473-1480 (2012).
10. Patti, M.E.*, et al.* Serum bile acids are higher in humans with prior gastric bypass: potential contribution to improved glucose and lipid metabolism. *Obesity (Silver Spring, Md.)* **17**, 1671-1677 (2009).
11. Scholtz, S.*, et al.* Obese patients after gastric bypass surgery have lower brain-hedonic responses to food than after gastric banding. *Gut* (2013).
12. Miras, A.D.*, et al.* Gastric bypass surgery for obesity decreases the reward value of a sweet-fat stimulus as assessed in a progressive ratio task. *The American journal of clinical nutrition* **96**, 467-473 (2012).
13. Pournaras, D.J.*, et al.* Remission of type 2 diabetes after gastric bypass and banding: mechanisms and 2 year outcomes. *Annals of surgery* **252**, 966-971 (2010).
14. Rubino, F., Schauer, P.R., Kaplan, L.M. & Cummings, D.E. Metabolic surgery to treat type 2 diabetes: clinical outcomes and mechanisms of action. *Annual review of medicine* **61**, 393-411 (2010).
15. Tarnoff, M., Shikora, S., Lembo, A. & Gersin, K. Chronic in-vivo experience with an endoscopically delivered and retrieved duodenal-jejunal bypass sleeve in a porcine model. *Surgical endoscopy* **22**, 1023-1028 (2008).
16. Gersin, K.S.*, et al.* Duodenal- jejunal bypass sleeve: a totally endoscopic device for the treatment of morbid obesity. *Surgical innovation* **14**, 275-278 (2007).
17. Sugerman, H.J., Wolfe, L.G., Sica, D.A. & Clore, J.N. Diabetes and hypertension in severe obesity and effects of gastric bypass-induced weight loss. *Annals of surgery* **237**, 751-756; discussion 757-758 (2003).

18. Rodriguez-Grunert, L., *et al.* First human experience with endoscopically delivered and retrieved duodenal-jejunal bypass sleeve. *Surgery for obesity and related diseases : official journal of the American Society for Bariatric Surgery* **4**, 55-59 (2008).
19. Gersin, K.S., *et al.* Open-label, sham-controlled trial of an endoscopic duodenojejunal bypass liner for preoperative weight loss in bariatric surgery candidates. *Gastrointestinal endoscopy* **71**, 976-982 (2010).
20. Schouten, R., *et al.* A multicenter, randomized efficacy study of the EndoBarrier Gastrointestinal Liner for presurgical weight loss prior to bariatric surgery. *Annals of surgery* **251**, 236-243 (2010).
21. Escalona, A., *et al.* Weight loss and metabolic improvement in morbidly obese subjects implanted for 1 year with an endoscopic duodenal-jejunal bypass liner. *Annals of surgery* **255**, 1080-1085 (2012).
22. de Moura, E.G., *et al.* Metabolic improvements in obese type 2 diabetes subjects implanted for 1 year with an endoscopically deployed duodenal-jejunal bypass liner. *Diabetes technology & therapeutics* **14**, 183-189 (2012).
23. Cohen, R., *et al.* Endoscopic Duodenal-Jejunal Bypass: Effect on Insulin Sensitivity in Mildly Obese Diabetic Subjects. in *72nd Scientific Sessions American Diabetes Association* (Philadelphia, USA, 2012).
24. Mathes, C.M. & Spector, A.C. Food selection and taste changes in humans after Roux-en-Y gastric bypass surgery: a direct-measures approach. *Physiology & behavior* **107**, 476-483 (2012).
25. Ochner, C.N., *et al.* Selective reduction in neural responses to high calorie foods following gastric bypass surgery. *Annals of surgery* **253**, 502-507 (2011).
26. Rodriguez, L., *et al.* Pilot clinical study of an endoscopic, removable duodenal-jejunal bypass liner for the treatment of type 2 diabetes. *Diabetes technology & therapeutics* **11**, 725-732 (2009).
27. Escalona, A., *et al.* Initial human experience with restrictive duodenal-jejunal bypass liner for treatment of morbid obesity. *Surgery for obesity and related diseases : official journal of the American Society for Bariatric Surgery* **6**, 126-131 (2010).
28. Breen, D.M., *et al.* Jejunal nutrient sensing is required for duodenal-jejunal bypass surgery to rapidly lower glucose concentrations in uncontrolled diabetes. *Nature medicine* **18**, 950-955 (2012).
29. Thomas, C., Pellicciari, R., Pruzanski, M., Auwerx, J. & Schoonjans, K. Targeting bile-acid signalling for metabolic diseases. *Nature reviews. Drug discovery* **7**, 678-693 (2008).
30. Ashrafian, H. & le Roux, C.W. Metabolic surgery and gut hormones - a review of bariatric entero-humoral modulation. *Physiology & behavior* **97**, 620-631 (2009).
31. Laferrere, B., *et al.* Rise of oxyntomodulin in response to oral glucose after gastric bypass surgery in patients with type 2 diabetes. *The Journal of clinical endocrinology and metabolism* **95**, 4072-4076 (2010).
32. de Luis, D., *et al.* Effects of duodenal-jejunal exclusion on beta cell function and hormonal regulation in Goto-Kakizaki rats. *American journal of surgery* **204**, 242-247 (2012).

33. Speck, M., Cho, Y.M., Asadi, A., Rubino, F. & Kieffer, T.J. Duodenal-jejunal bypass protects GK rats from {beta}-cell loss and aggravation of hyperglycemia and increases enteroendocrine cells coexpressing GIP and GLP-1. *American journal of physiology. Endocrinology and metabolism* **300**, E923-932 (2011).

34. Lee, H.C., Kim, M.K., Kwon, H.S., Kim, E. & Song, K.H. Early changes in incretin secretion after laparoscopic duodenal-jejunal bypass surgery in type 2 diabetic patients. *Obesity surgery* **20**, 1530-1535 (2010).

35. Rubino, F., *et al.* The mechanism of diabetes control after gastrointestinal bypass surgery reveals a role of the proximal small intestine in the pathophysiology of type 2 diabetes. *Annals of surgery* **244**, 741-749 (2006).

36. Kindel, T.L., Yoder, S.M., Seeley, R.J., D'Alessio, D.A. & Tso, P. Duodenal-jejunal exclusion improves glucose tolerance in the diabetic, Goto-Kakizaki rat by a GLP-1 receptor-mediated mechanism. *Journal of gastrointestinal surgery : official journal of the Society for Surgery of the Alimentary Tract* **13**, 1762-1772 (2009).

37. De Silva, A., *et al.* The gut hormones PYY 3-36 and GLP-1 7-36 amide reduce food intake and modulate brain activity in appetite centers in humans. *Cell metabolism* **14**, 700-706 (2011).

38. Dumas, M.E., *et al.* Metabolic profiling reveals a contribution of gut microbiota to fatty liver phenotype in insulin-resistant mice. *Proceedings of the National Academy of Sciences of the United States of America* **103**, 12511-12516 (2006).

39. Nicholson, J.K., Holmes, E. & Wilson, I.D. Gut microorganisms, mammalian metabolism and personalized health care. *Nature reviews. Microbiology* **3**, 431-438 (2005).

40. Williams, H.R., *et al.* Characterization of inflammatory bowel disease with urinary metabolic profiling. *The American journal of gastroenterology* **104**, 1435-1444 (2009).

41. Trygg, J., Holmes, E. & Lundstedt, T. Chemometrics in metabonomics. *Journal of proteome research* **6**, 469-479 (2007).

42. Calvani, R., *et al.* Gut microbiome-derived metabolites characterize a peculiar obese urinary metabotype. *International journal of obesity (2005)* **34**, 1095-1098 (2010).

43. Mutch, D.M., *et al.* Metabolite profiling identifies candidate markers reflecting the clinical adaptations associated with Roux-en-Y gastric bypass surgery. *PloS one* **4**, e7905 (2009).

44. Zhang, H., *et al.* Human gut microbiota in obesity and after gastric bypass. *Proceedings of the National Academy of Sciences of the United States of America* **106**, 2365-2370 (2009).

45. Turnbaugh, P.J., *et al.* An obesity-associated gut microbiome with increased capacity for energy harvest. *Nature* **444**, 1027-1031 (2006).

46. Olbers, T., *et al.* Body composition, dietary intake, and energy expenditure after laparoscopic Roux-en-Y gastric bypass and laparoscopic vertical banded gastroplasty: a randomized clinical trial. *Annals of surgery* **244**, 715-722 (2006).

47. le Roux, C.W., *et al.* Gastric bypass reduces fat intake and preference. *American journal of physiology. Regulatory, integrative and comparative physiology* **301**, R1057-1066 (2011).

48. Carey, V.J., *et al.* Body fat distribution and risk of non-insulin-dependent diabetes mellitus in women. The Nurses' Health Study. *American journal of epidemiology* **145**, 614-619 (1997).

49. Manson, J.E., *et al.* A prospective study of obesity and risk of coronary heart disease in women. *The New England journal of medicine* **322**, 882-889 (1990).

50. Calle, E.E., Thun, M.J., Petrelli, J.M., Rodriguez, C. & Heath, C.W., Jr. Body-mass index and mortality in a prospective cohort of U.S. adults. *The New England journal of medicine* **341**, 1097-1105 (1999).

51. Seshasai, S.R., *et al.* Diabetes mellitus, fasting glucose, and risk of cause-specific death. *The New England journal of medicine* **364**, 829-841 (2011).

52. Association, A.D. Standards of Medical Care in Diabetes—2013. *Diabetes care* **36**, S11-S66 (2013).

53. Oldfield, R.C. The assessment and analysis of handedness: the Edinburgh inventory. *Neuropsychologia* **9**, 97-113 (1971).

54. Inzucchi, S.E., *et al.* Management of Hyperglycemia in Type 2 Diabetes: A Patient-Centered Approach: Position Statement of the American Diabetes Association (ADA) and the European Association for the Study of Diabetes (EASD). *Diabetes care* **35**, 1364-1379 (2012).

55. Craig, C.L., *et al.* International physical activity questionnaire: 12-country reliability and validity. *Med.Sci.Sports Exerc.* **35**, 1381-1395 (2003).

56. Rankinen, T. & Bouchard, C. Genetics of food intake and eating behavior phenotypes in humans. *Annual review of nutrition* **26**, 413-434 (2006).

57. Bell, C.G., Walley, A.J. & Froguel, P. The genetics of human obesity. *Nature reviews. Genetics* **6**, 221-234 (2005).

58. Miller, F.A., Ahern, C., Smith, C.A. & Harvey, E.A. Understanding the new human genetics: a review of scientific editorials. *Social science & medicine* **62**, 2373-2385 (2006).

59. Kroke A., Klipstein-Grobusch K., Voss S., Möseneder J., Thielecke F., Noack R., Boeing H. Validation of a self-administered food-frequency questionnaire administered in the European Prospective Investigation into Cancer and Nutrition (EPIC) Study: comparison of energy, protein, and macronutrient intakes estimated with the doubly labeled water, urinary nitrogen, and repeated 24-h dietary recall methods. *AmJ Clin Nutr* **70**, 439-47 (1999).

60. van Strien, T., Frijters, J.E.R., Bergers, G.P.A. & Defares, P.B. The Dutch Eating Behavior Questionnaire (DEBQ) for assessment of restrained, emotional, and external eating behavior. *Int J Eat Disorder* **5**, 295-315 (1986).

61. Fairburn, C.G. & Beglin, S.J. Assessment of eating disorders: interview or self-report questionnaire? *The International journal of eating disorders* **16**, 363-370 (1994).

62. Carver, C.S. & White, T.L. Behavioral-Inhibition, Behavioral Activation, and Affective Responses to Impending Reward and Punishment - the Bis Bas Scales. *J Pers Soc Psychol* **67**, 319-333 (1994).

63. Beck, A.T., Steer, R.A. & Brown, G.K. *BDI-II, Beck depression inventory*

manual, (Psychological Corp, San Antonio, Tex, 1996).

64. Patton, J.H., Stanford, M.S. & Barratt, E.S. Factor structure of the Barratt impulsiveness scale. *Journal of clinical psychology* **51**, 768-774 (1995).
65. Yeomans, M.R., Leitch, M. & Mobini, S. Impulsivity is associated with the disinhibition but not restraint factor from the Three Factor Eating Questionnaire. *Appetite* **50**, 469-476 (2008).
66. Heatherton, T.F., Kozlowski, L.T., Frecker, R.C. & Fagerstrom, K.O. The Fagerstrom Test for Nicotine Dependence - a Revision of the Fagerstrom Tolerance Questionnaire. *Brit J Addict* **86**, 1119-1127 (1991).
67. Stunkard, A.J. & Messick, S. The three-factor eating questionnaire to measure dietary restraint, disinhibition and hunger. *J Psychosom.Res* **29**, 71-83 (1985).
68. Watson, D., Clark, L.A. & Tellegen, A. Development and validation of brief measures of positive and negative affect: the PANAS scales. *J Pers.Soc.Psychol.* **54**, 1063-1070 (1988).
69. Killgore, W.D.S. & Yurgelun-Todd, D.A. Affect modulates appetite-related brain activity to images of food. *Int J Eat Disorder* **39**, 357-363 (2006).
70. Gearhardt, A.N., Corbin, W.R. & Brownell, K.D. Preliminary validation of the Yale Food Addiction Scale. *Appetite* **52**, 430-436 (2009).
71. Babor, T., Higgins-Biddle, J., Saunders, J. & Monteiro, M. AUDIT: The Alcohol Use Disorders Identification Test: Guidelines for Use in Primary Care. (2001).
72. Lowe, M.R., *et al.* The Power of Food Scale. A new measure of the psychological influence of the food environment. *Appetite* **53**, 114-118 (2009).
73. Buysse, D.J., Reynolds, C.F., 3rd, Monk, T.H., Berman, S.R. & Kupfer, D.J. The Pittsburgh Sleep Quality Index: a new instrument for psychiatric practice and research. *Psychiatry research* **28**, 193-213 (1989).
74. Sigstad, H. A clinical diagnostic index in the diagnosis of the dumping syndrome. Changes in plasma volume and blood sugar after a test meal. *Acta medica Scandinavica* **188**, 479-486 (1970).
75. Arts, J., *et al.* Efficacy of the long-acting repeatable formulation of the somatostatin analogue octreotide in postoperative dumping. *Clinical gastroenterology and hepatology : the official clinical practice journal of the American Gastroenterological Association* **7**, 432-437 (2009).
76. Gormally, J., Black, S., Daston, S. & Rardin, D. The assessment of binge eating severity among obese persons. *Addictive behaviors* **7**, 47-55 (1982).
77. Herrmann, C. Screening for major depression in a group of diabetic patients. *Psychosomatic medicine* **59**, 559-560 (1997).
78. Stewart, A.L., Hays, R.D. & Ware, J.E.J. The MOS Short-form General Health Survey: Reliability and Validity in a Patient Population. *Medical Care* **26**, 724-735 (1988).
79. Kirby, K.N. & Marakovic, N.N. Delay-discounting probabilistic rewards: Rates decrease as amounts increase. *Psychonomic bulletin & review* **3**, 100-104 (1996).

80. Kirby, K.N. & Petry, N.M. Heroin and cocaine abusers have higher discount rates for delayed rewards than alcoholics or non-drug-using controls. *Addiction* **99**, 461-471 (2004).

81. Perry, J.L. & Carrol, M.E. The role of impulsive behavior in drug abuse. *Psychopharmacology* **200**, 1-26 (2008).

82. Weller, R.E., Cook, E.W., 3rd, Avsar, K.B. & Cox, J.E. Obese women show greater delay discounting than healthy-weight women. *Appetite* **51**, 563-569 (2008).

83. Davis, C., Patte, K., Curtis, C. & Reid, C. Immediate pleasures and future consequences. A neuropsychological study of binge eating and obesity. *Appetite* **54**, 208-213 (2010).

84. Finlayson, G., King, N. & Blundell, J. The role of implicit wanting in relation to explicit liking and wanting for food: implications for appetite control. *Appetite* **50**, 120-127 (2008).

85. Haidt, J., Mccauley, C. & Rozin, P. Individual-Differences in Sensitivity to Disgust - a Scale Sampling 7 Domains of Disgust Elicitors. *Pers Indiv Differ* **16**, 701-713 (1994).

86. Olatunji, B.O., *et al.* The Disgust Scale: Item analysis, factor structure, and suggestions for refinement. *Psychological Assessment* **19**, 281-297 (2007).

87. Petersen, E.T., Zimine, I., Ho, Y.-C.L. & Golay, X. Non-invasive measurement of perfusion: a critical review of arterial spin labelling techniques. *The British Journal of Radiology* **79**, 688-701 (2006).

88. Paiva, F.F., Tannús, A. & Silva, A.C. Measurement of cerebral perfusion territories using arterial spin labelling. *NMR in Biomedicine* **20**, 633-642 (2007).

89. Damoiseaux, J.S., *et al.* Consistent resting-state networks across healthy subjects. *Proceedings of the National Academy of Sciences* **103**, 13848-13853 (2006).

90. Goldstone, A.P., *et al.* Fasting biases brain reward systems towards high-calorie foods. *The European journal of neuroscience* **30**, 1625-1635 (2009).

91. Knutson, B., Adams, C.M., Fong, G.W. & Hommer, D. Anticipation of increasing monetary reward selectively recruits nucleus accumbens. *The Journal of neuroscience : the official journal of the Society for Neuroscience* **21**, Rc159 (2001).

92. Knutson, B., Westdorp, A., Kaiser, E. & Hommer, D. FMRI visualization of brain activity during a monetary incentive delay task. *NeuroImage* **12**, 20-27 (2000).

93. Horn, N.R., Dolan, M., Elliott, R., Deakin, J.F. & Woodruff, P.W. Response inhibition and impulsivity: an fMRI study. *Neuropsychologia* **41**, 1959-1966 (2003).

94. Li, C.S.R., *et al.* Error-specific medial cortical and subcortical activity during the stop signal task: A functional magnetic resonance imaging study. *Neuroscience* **155**, 1142-1151 (2008).

95. Berger, S. The WAIS-R factors: usefulness and construct validity in neuropsychological assessments. *Applied neuropsychology* **5**, 37-42 (1998).

96. Bueter, M., *et al.* Alterations of sucrose preference after Roux-en-Y gastric bypass. *Physiology & behavior* **104**, 709-721 (2011).

97. Dixon, J.B., *et al.* Bariatric surgery: an IDF statement for obese Type 2 diabetes. *Diabetic medicine : a journal of the British Diabetic Association* **28**, 628-642 (2011).

98. Gaede, P., Lund-Andersen, H., Parving, H.H. & Pedersen, O. Effect of a multifactorial intervention on mortality in type 2 diabetes. *The New England journal of medicine* **358**, 580-591 (2008).

99. Philips, Z., *et al.* Review of guidelines for good practice in decision-analytic modelling in health technology assessment. *Health technology assessment* **8**, iii-iv, ix-xi, 1-158 (2004).

100. Caro, J.J., Briggs, A.H., Siebert, U., Kuntz, K.M. & Force, I.-S.M.G.R.P.T. Modeling good research practices--overview: a report of the ISPOR-SMDM Modeling Good Research Practices Task Force-1. *Medical decision making : an international journal of the Society for Medical Decision Making* **32**, 667-677 (2012).

101. Computer Modeling of Diabetes and Its Complications: A report on the Fourth Mount Hood Challenge Meeting. *Diabetes care* **30**, 1638-1646 (2007).

102. Stevens, R.J., Kothari, V., Adler, A.I., Stratton, I.M. & United Kingdom Prospective Diabetes Study, G. The UKPDS risk engine: a model for the risk of coronary heart disease in Type II diabetes (UKPDS 56). *Clinical science* **101**, 671-679 (2001).

103. Clarke, P., Gray, A. & Holman, R. Estimating utility values for health states of type 2 diabetic patients using the EQ-5D (UKPDS 62). *Medical decision making : an international journal of the Society for Medical Decision Making* **22**, 340-349 (2002).

SIGNATURE PAGE 1 (Chief Investigator)

The signature below constitutes approval of this protocol by the signatory and provides the necessary assurances that this study will be conducted according to all stipulations of the protocol including all statements regarding confidentiality.

Study Title: A randomized controlled trial of a duodenal sleeve bypass device (EndoBarrier) compared with standard medical therapy for the management of obese subjects with type 2 diabetes

Protocol Number: 5.0

Signed:

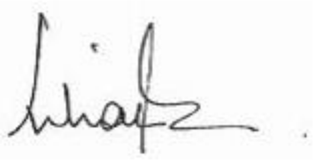

Prof Julian Teare

Date: 20/03/2018

Formatted: Superscript

SIGNATURE PAGE 2 (SPONSOR)

The signatures below constitute approval of this protocol by the signatory.

Study Title: A randomized controlled trial of a duodenal sleeve bypass device (EndoBarrier) compared with standard medical therapy for the management of obese subjects with type 2 diabetes

Protocol Number: 5.0~~4.1~~

Signed: ______________________

Mrs Ruth Nicholson
Research Governance Manager, Faculty of Medicine Centre at Imperial College London

Date: 20/03/18

SIGNATURE PAGE 3 (STATISTICIAN)

The signatures below constitute approval of this protocol by the signatory.

Study Title: A randomized controlled trial of a duodenal sleeve bypass device (EndoBarrier) compared with standard medical therapy for the management of obese subjects with type 2 diabetes

Protocol Number: 5.0

Signed: Emanuela Falaschetti ____________________

Ms Emanuela Falaschetti
Research Fellow in Clinical Trial Statistics at the Imperial Clinical Trial Unit, Imperial College London, School of Public Health

Date: 20/3/18

SIGNATURE PAGE 4 (INVESTIGATOR)

The signature of the below constitutes agreement of this protocol by the signatory and provides the necessary assurance that this study will be conducted at his/her investigational site according to all stipulations of the protocol including all statements regarding confidentiality.

Study Title: A randomized controlled trial of a duodenal sleeve bypass device (EndoBarrier) compared with standard medical therapy for the management of obese subjects with type 2 diabetes

Protocol Number: ~~4.1~~5.0

Address of Institution: Dept of Surgery
University Hospital Southampton
Southampton SO16 6YD

Signed: [signature]

Print Name and Title: Mr James Byrne

Date: April 8, 2018.
