# Supplementary material for: Duodenal-Jejunal Bypass Liner for the management of Type 2 Diabetes Mellitus and Obesity: A Multicenter Randomized Controlled Trial
Source: Ann Surg. 2021 Jun 15;275(3):440–7. doi: 10.1097/SLA.0000000000004980 (PMC8820769; doi:10.1097/SLA.0000000000004980)
Supplement: Supplemental Digital Content [file ansu-275-0440-s002.pdf]

Statistical Analysis Plan (SAP)

A randomized controlled trial of a duodenal sleeve bypass device (EndoBarrier) compared with standard medical therapy for the management of obese subjects with type 2 diabetes

EndoBarrier

14SM2015

Study Investigators:

Chief Investigator: Prof. Julian Teare

Study Management: Imperial Clinical Trials Unit (ICTU)

SAP Working Group:

Emanuela Falaschetti – Senior Statistician

Nicholas Johnson – Study Statistician

Version	Date	Author
1.0	12FEB2019	Nicholas Johnson

Approved by:

Name	Signature	Role	Date
Prof. Julian Teare	[signature]	Chief Investigator	11/2/19.
Prof. Jonathan Brown	[signature]	TSC Chair	4/3/19
Emanuela Falaschetti	[signature]	Senior Statistician	12/02/2019
Nicholas Johnson	[signature]	Study Statistician	12/02/2019

A randomized controlled trial of a duodenal sleeve bypass device (EndoBarrier) compared with standard medical therapy for the management of obese subjects with type 2 diabetes

14SM2015

Table of Contents

2. Study Objectives / Hypotheses Testing 4
2.1. Primary Objectives 4
2.2. Secondary Objectives 4
2.3. Safety Objective 4
3. Study Endpoints 5
3.1. Primary Endpoint 5
3.2. Secondary Endpoints 5
4. Background/Introduction 6
4.1. Introduction 6
4.2. Study Design 6
4.3. Treatment Groups 7
4.4. Study Population 7
4.5. Sample Size 8
4.6. Schedule of Time and Events 10
4.7. Randomisation 11
5. Analysis Set 12
6. Variables of Analysis 12
6.1. Primary Endpoint Variable 12
6.2. Secondary Endpoint Variables 12
6.2.1. Efficacy Variables 12
6.2.2. Mechanistic Sub-groups 12
6.2.3. Metabonomic Analysis 14
6.2.4. Cost Effectiveness 14
6.2.5. Safety Variables 14
6.3. Exploratory Efficacy Analysis 15
7. Statistical Methodology 15
7.1. Baseline Demographics 15
7.2. Primary Analysis 15
7.3. Secondary Analysis 15
7.3.1. Secondary Efficacy Analysis: 15
7.3.2. Mechanistic Sub-Group Analysis 16
7.3.3. Metabonomic Analysis 17

7.3.4. **Exploratory Efficacy Analysis** 17
7.3.5. **Cost Effectiveness** 18
7.4. **Safety Analysis** 18
7.5. **Interim Analysis** 18
7.6. **Missing Data and Withdrawn Subjects** 18
8. **Tables to Present** 20
8.1. **Subject Disposition** 20
8.2. **Protocol Deviations & Violations** 21
8.3. **Baseline Demographics** 21
8.4. **Primary Endpoint** 23
8.5. **Secondary Endpoints** 25
8.6. **Mechanistic Endpoints** 31
8.6.1. **All Subgroups** 31
8.6.2 **Additional Mechanistic Output for Food Preference Subgroup** 39
8.6.3 **Additional Mechanistic Output for FMRI Subgroup** 44
8.6.4 **Additional Mechanistic Output for the Insulin Clamp** 47
8.7 **Exploratory Efficacy Endpoints** 48
8.8 **Safety Endpoints** 50

2. Study Objectives / Hypotheses Testing

2.1. Primary Objectives

To compare the EndoBarrier with conventional medical therapy, diet and exercise for obesity related type II diabetes and their effectiveness on metabolic state as defined by the International Diabetes Federation (IDF) with an HbA1c reduced by 20%.

2.2. Secondary Objectives

To compare the EndoBarrier with conventional medical therapy, diet and exercise for obesity related type II diabetes and their effectiveness on:

- Metabolic state as defined by the International Diabetes Federation (IDF) with an HbA1c < 6% (or < 42 mmol/mol)
- Blood pressure < 135/85
- Absolute weight loss

To investigate the mechanism of the effect of the EndoBarrier via changes in:

- Gut hormones
- Microbiome
- Appetite, food hedonics and brain reward systems
- Body fat content
- Food preference
- Hepatic or peripheral insulin sensitivity
- Bile acids
- Biomarkers such as genetic markers

To estimate the cost-effectiveness of the EndoBarrier device compared with conventional treatment over the trial period (within trial analysis).

To estimate the long-term cost-effectiveness (over 24 months) of the EndoBarrier device compared with conventional treatment and alternative surgical interventions.

2.3. Safety Objective

To evaluate the safety of the EndoBarrier device.

3. Study Endpoints

3.1. Primary Endpoint

- Subjects showing a reduction in HbA1c by 20%, to be measured 12 months from start of treatment.

3.2. Secondary Endpoints

Secondary efficacy endpoints for the Endobarrier trial are listed as below:

- HbA1c of less than 6% (or 42 mmol/mol)
- Blood pressure below 135/85 mmHg
- Absolute weight loss greater than 15%
- Reduction in dose/or number of medications
- Frequency of Adverse Events

Additional endpoints investigating the secondary mechanistic objective effects of the EndoBarrier device are defined as below*:

- Questionnaires (DEBQ, EDE-Q, BIS/BAS, BDI-II, Barratt, Fagerstrom, TFEQ, IPAQ, Yale FAS, AUDIT, Power of Food, PSQI, Dumping Syndrome, HADS, SF36, PANAS, EPQR)
- Visual Analogue Rating Scales (Hunger, Fullness, Nausea, Pleasantness/Volume to Eat, Sleepiness, Anxiety, Stress)
- Eating and Behavioural Computerised Tasks (Delay Discounting, Leeds Food Choice, Progressive Ratio)
- Dietary Energy Intake (24h Dietary Recall, EPIC FFQ, Three-Days Food Diary)
- Food Preference Tasting Tests (Sweet Taste Detection – EC50 & Corrected Hit Rate, Consummatory Taste Reward – Just About Right, Pleasantness & Intensity)
- Bio-Electrical Impedance (%age Body Fat, Total Fat & Fat-Free Mass)
- Fasting Bloods
- Post-Prandial Bloods
- Urine & Faecal metabolomics analysis
- Biopsy analysis for RNA & DNA (Stomach & Duodenum)
- Insulin Clamp sample analysis (Glucose, Infusates, Insulin, Glucagon, NEFAs, C-Peptide)
- MRI Testing (Resting State fMRI, Food Picture Evaluation, Go No-Go Task, Emotional Reactivity Task, Diffusion Tensor Imaging, T1/T2, Monetary Incentive Task, Arterial Spin Labelling)
- Microbiome
- Biomarkers such as genetic markers

**A detailed breakdown of the above can be found in Appendix A*

Additional secondary endpoints investigating the cost benefit of the EndoBarrier device will be assessed by a health economist and defined within a separate document. This will cover the following secondary objectives:

- Cost of interventions and related health and social care
- QALYs accrued (calculated from area under the EQ-5D curve)
- Incremental cost per QALY within the trial period and over the long-term

4. Background/Introduction

4.1. Introduction

Obesity is a serious medical condition which is increasing in incidence worldwide. Obesity induces metabolic abnormalities which contribute to the development of diabetes mellitus and cardiovascular disease. The treatment modalities currently available for the treatment of obesity (i.e. lifestyle interventions, pharmacotherapy and surgery) have limited long-lasting success in producing major and sustained weight loss and are often associated with undesirable side effects, risks or complications. Therefore the need for new exits and effective strategies is necessary to prevent and reduce obesity and its complications such as T2DM. This study is a randomised, placebo-controlled trial which has been designed to further investigate the potential of the EndoBarrier device as an effective alternative treatment to surgery and existing medical therapies in T2DM.

If the EndoBarrier is effective at achieving long-lasting weight loss and glycaemic control, there is an obvious potential for savings on future health and social care; through the avoidance of diabetes and related complications. However, the overall cost-effectiveness of the EndoBarrier device depends on the balance of health benefits, harms, costs and savings compared with other surgical and medical treatment options. In addition to a 'within trial' economic analysis, modelling will be used to estimate lifetime impacts on morbidity, mortality and expenditure, and hence to evaluate whether the EndoBarrier offers the NHS good value for money.

4.2. Study Design

This study is a randomised controlled trial of the EndoBarrier device compared with standard medical therapy for the management of obese subjects with T2DM. Subjects will be randomised to one of the two treatment arms (see Table 1 within section 3.3) of the study via the InForm system (the eCRF database for the study). Over 24 months, the study will be performed at two investigational sites, Imperial College Healthcare NHS Trust in London and University Hospital Southampton NHS Foundation Trust.

Individuals in both study arms will be invited regular medical check-ups including measurement of weight, blood pressure, and blood parameters (HbA1c, cholesterol, triglycerides, fasting blood glucose, insulin), quality of life (EQ-5D) and use of health services, as well as to record any adverse events and medications. Diabetes medication titrations will be conducted by the study diabetologists/endocrinologists in accordance with the guidelines of the American Diabetes Association (see section 5.5.1 for full details). They will also receive routine dietary and exercise counselling as well as telephone counselling from a specialist dietician. The control arm will be invited for routine follow-up visits to review their standard medical care. Both arms will also be invited to undergo a diversity of tests investigating the mechanism of the EndoBarrier. After 12 months, the EndoBarrier will be removed and both arms will be followed up for a remaining 12 months.

In order to investigate the mechanism of the effect of the EndoBarrier Device, the trial is divided into three sub-groups in each arm who will have the following additional assessments at visits 3, 5, 8 and 10, 14 (see section 5.4 and 5.6 for more detail):

- Sub-group 1 (minimum n=24): functional MRI, eating behaviour, cognitive assessment, and post-meal gut hormones.
- Sub-group 2 (minimum n=18): insulin clamps
- Sub-group 3 (minimum n=18): assessment of taste and food preference, eating behaviour assessment, and post-meal gut hormones

Participation in the sub-groups is optional.

4.3. Treatment Groups

Table 1. Summary of treatment groups

Treatment Sequence	Number of subjects*	Treatment Period 1	Follow-up Period 2
1 - EndoBarrier Device	80	12 months	12 months
2 - Standard Medical therapy	80	12 months	12 months
Total number of subjects	160		

* Additional subjects may be recruited to allow for any randomised subject that has withdrawn prior to commencement of treatment at Visit 4.

4.4. Study Population

The EndoBarrier study is recruiting patients between the ages of 18 and 65 that have had T2DM for at least 1 year (HbA1c 7.5-11.0% = 58-97 mmol/mol) and are on oral T2DM medications.

In order to be eligible for the study the patient must not have any of the following:

- BMI outside the range of 30-50 kg/m^2
- Females of childbearing potential who are pregnant, breast-feeding or intend to become pregnant or are not using adequate or reliable contraceptive methods
- Evidence of absolute insulin deficiency as indicated by clinical assessment, a long duration of T2DM and a fasting plasma C-peptide of <333pmol/L
- Current use of insulin
- Previous diagnosis with Type 1 DM or a history of ketoacidosis
- Requirement of NSAIDs (non-steroidal anti-inflammatory drugs) or prescription of anticoagulation therapy during the implant period
- Current iron deficiency and/or iron deficiency anaemia
- Symptomatic gallstones or kidney stones at the time of screening
- History of coagulopathy, upper gastro-intestinal bleeding conditions such as oesophageal or gastric varices, congenital or acquired intestinal telangiectasia
- Previous GI surgery that could affect the ability to place the device or the function of the implant
- History or presence of active H. pylori (if subjects are randomised into the EndoBarrier arm and have a history or presence of active H. pylori – tested during study visit 2 - they can receive appropriate treatment and then subsequently enrol into the study)

- Family history of a known diagnosis or pre-existing symptoms of systemic lupus erythematosus, scleroderma or other autoimmune connective tissue disorder
- Severe liver impairment i.e. AST, ALT or gGT >4 times upper limit of the reference range or kidney impairment i.e. estimated Glomerular Filtration Rate (GFR) < 45 ml/min/1.73m2
- Severe depression, unstable emotional or psychological characteristics (indicated by Beck Depression Inventory II score >28)
- Poor dentition and inability to adequately chew food
- Metal implant unsuitable for MRI scanning and claustrophobia as contraindications for MRI scans (sub-group 1 - fMRI study only)
- Vegetarian, vegan, gluten or lactose intolerance as unsuitable for fMRI food picture paradigm (sub-group 1 – fMRI only)

At the screening visit, the patient will be examined to ensure that none of the exclusion criteria are met. Their general health will be assessed via a series of tests including; body weight, height, waist circumference, blood pressure, ECG, urine dipstick and pregnancy test, blood parameters. Female patients will also be asked to report the last day of their menstrual period, the length of their cycle and the length of their menstruation (bleeding) to ensure they are not pregnant and to monitor any changes in their menstrual cycle during the course of the study.

If the patient does not meet any of the exclusion criteria and no additional complications are encountered during the screening process then once written, informed consent has been received, the patient is eligible for randomisation.

Patients will be given the opportunity to consent for participation in one of the mechanistic sub-groups (1-3) of this study. Following informed consent, patients interested in consenting for participation in Sub-group 1 of the study will also be screened for suitability by checking the patient does not self-report to be vegetarian, vegan, gluten or lactose intolerant (to be recorded in the CRF). They will also be checked for their fMRI suitability using these questionnaires:

(i) Metal check form - to ensure safety for MRI scanning, as may preclude entry into fMRI study

(ii) Handedness Inventory - inability to use a right-handed button pad will preclude entry into fMRI study using Handedness.

4.5. Sample Size

The primary end-point of a 20% reduction in HbA1c has been chosen as the International Diabetes Federation produced in June 2011 new guidelines for the conduct of studies in diabetes using bariatric surgery or devices aiming to produce standardisation allowing comparison between studies. To date there are thus no published large patient group studies using this end-point, so using this new endpoint in a well-designed and conducted study will be of scientific value in itself.

Conservatively, it was estimated that 15% of patients in the control arm will achieve the target but believe this to be an overestimate. The Steno study is the best quality randomised study (80 patients in each arm) into the effect of best medical therapy published to date and demonstrated over an average 7.8 years significant improvements in HbA1c amongst those having intensive medical therapy from 8.4+/- 1.6 to 7.7+/-1.2, but no change in HbA1c amongst those continuing with standard medical therapy. This study defines the very best that could realistically be achieved in the control arm, but expect there to be very little if any change in this group. The reporting of

HbA1c as an outcome measure was not in accordance with the newly defined IDF criteria, but considering the small average reduction achieved in the Steno study, it will be assumed that a target of 15% of patients reaching the endpoint is a conservative estimate. Company data on the small number of patients who have reached a year with the device in place suggest that 40% will achieve this target.

According to our own experience with the device in the commercially sponsored study, up to 30% of patients in the treatment group may have the device removed early. Nevertheless other commercially sponsored (unpublished) studies of this device have achieved lower explant rates (J Tetreault – GI Dynamics). We have therefore diluted the treatment effect from 40% vs. 15% to 35% vs. 15% achieving the target of 20% reduction in HbA1c for treatment arm vs. standard arm. 73 patients per group will give 80% power to detect a significant effect. Adding 10% loss of follow-up increases the sample size to 80 per group.

The dilution was calculated starting from the assumption that 40% of patients with the device will reach the target (this estimate is based on company data based on diabetic patients in the same range of BMI as in the present proposal). If 30% of patients in the treatment group need to remove the device early but remain available for follow-up, in the worst case scenario, the proportion reaching the target is the same as in the control group, bringing the estimate for the treatment group to 32.5%. However most of them will keep the device for some time, having some benefit, so it is plausible to assume that the estimate is higher than 32.5%.

Dividing the main effect 15% vs. 40% in three parts we assume that in the 30% of patients with removal, for 1/3 the same effect will be achieved as in the control group (15% reaching the target), for 1/3 it will be increased (23% reach the target) and for 1/3 more increased (31% reach the target). Overall, this would give an estimate of 35% for the treatment group.

For that reason, 2 arms of 80 patients will be sufficient to ensure demonstration of a significant effect (if one exists) and very conservatively allows for explant rates of up to 30%, a higher, level of benefit in the control arm than is likely to be achieved, and drop-out rates of 10%.

Furthermore the landmark Steno study which in some ways may be considered similar to this study, that had in all likelihood a less effective intervention arm was sufficiently powered with 80 patients in each arm.

4.6. Schedule of Time and Events

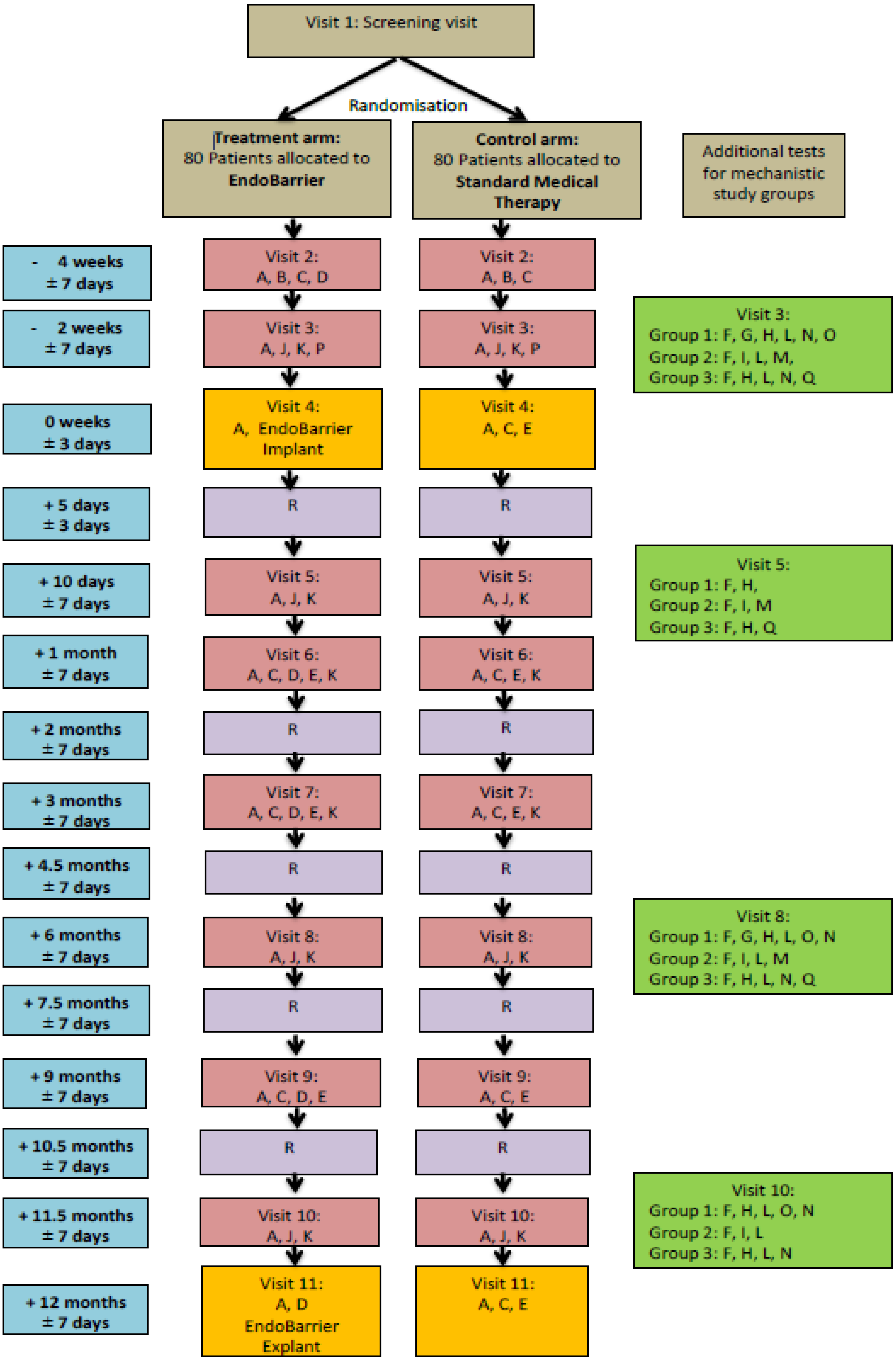

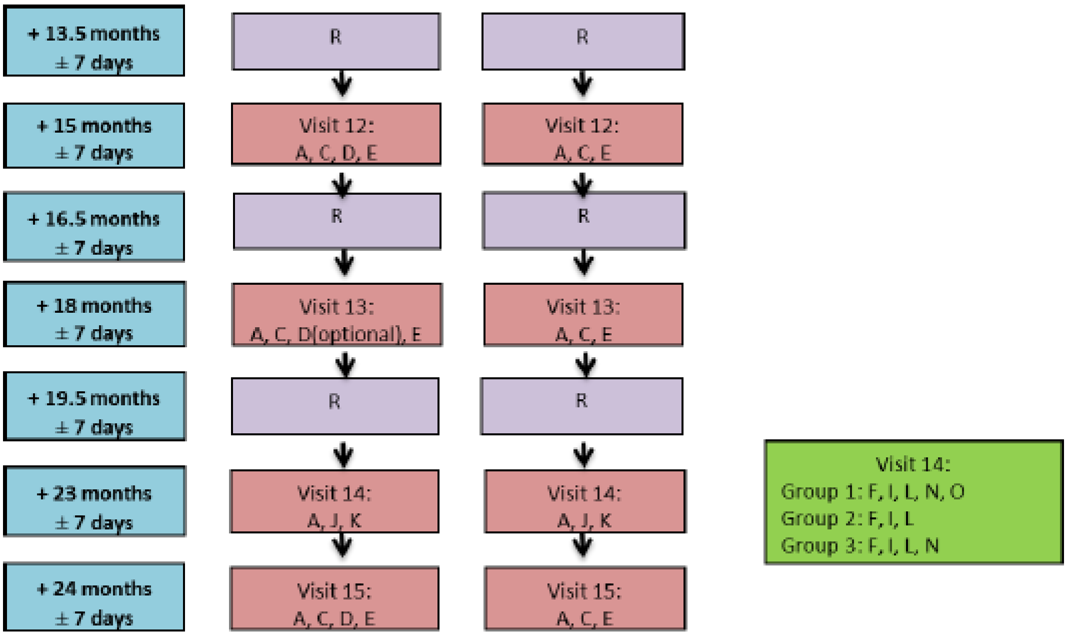

Legend:
A – Weight, waist, blood pressure, routine bloods, adverse events, changes in medication/medical history
B – Dietary Counselling
C – Medical Therapy (Diabetologist/Endocrinologist)
D – Gastroenterologist
E – Dietician Follow-up
F – Bioelectrical Impedance
G – fMRI
H – Gut hormones (fasting and post-meal profile)
I – Gut hormones (fasting only)
J – Metabolomics
K – Health Economics questionnaires
L – Eating and Behaviour questionnaires
M – Insulin clamps
N – Eating behaviour computerised tasks
O – Cognitive assessment tasks
P – DNA Sample
Q – Food preference and taste assessment
R – Telephone counselling

4.7. Randomisation

After the screening visit, all eligible patients for the trial will be randomised into one of the two arms of the study via the InForm system (the eCRF database for the study) which will be programmed with a randomisation schedule provided by an independent statistician. This will protect against bias in the randomisation process as patients are allocated automatically. The randomisation will be stratified by site and two BMI groups, 30-40 and 40-50 kg/m2. The subjects will be informed about their allocated treatment arm on visit 2.

Only the subject number and subject initials will be recorded in the case report form, and if the subjects name appears on any other document (e.g. pathologist report) it will be completely anonymised. The investigator will maintain a personal subject identification list (subject numbers with the corresponding subject names) to enable records to be identified.

5. Analysis Set

All summary tables and listings produced for the trial report will show the allocated randomisation group and will be performed according to the intention to treat principle.

All randomised subjects will be considered part of the analysis population. A sensitivity analysis will also be performed excluding patients that have withdrawn from the study during the run-in phase between randomisation (Visit 2) and start of treatment (Visit 4). Any subjects withdrawn during this period will still be required to attend the end-of-study follow-up visit.

6. Variables of Analysis

6.1. Primary Endpoint Variable

In order to satisfy our primary objective we will be looking for a reduction in glycosylated haemoglobin levels (HbA1c) at 12 months from start of treatment by 20%.

6.2. Secondary Endpoint Variables

6.2.1. Efficacy Variables

In order to compare the EndoBarrier with conventional medical therapy, diet and exercise for obesity related type II diabetes we will be studying the following at 12 months from start of treatment:

- HbA1c levels; values of less than 6% (or 42 mmol/mol)
- Blood pressure; values below 135/85 mmHg
- Weight; to investigate an absolute weight loss greater than 15%, body fat content.

6.2.2. Mechanistic Sub-groups

In order to investigate the mechanism of the effect of the EndoBarrier we will be analysing the following:

- Questionnaires: DEBQ, EDE-Q, BIS/BAS, BDI-II, Barratt Impulsivity Scale & UPPS-P, Fagerstrom Nicotine Dependence Scale, TFEQ, IPAQ, Yale Food Addiction Scale, AUDIT, Power of Food, PSQI, Dumping Syndrome, HADS, SF36, PANAS, Barratt Impulsivity Score, EPQR. Data will be analysed across Visits 3, 8, 10 & 14 for all questionnaires with the exception of Dumping Syndrome which will include for Visits 3, 6, 7, 8, 10 & 14.
- VAR Scales will be scored out of 100 for Hunger, Fullness, Nausea, Pleasantness, Volume, Sleepiness, Anxiety & Stress across Visits 3, 5, 8, 10 & 14.
- Food Diary: A three-day food diary will be taken at Visits 3, 5, 8, 10 & 14.
- Food Preference Testing: Sweet Taste Detection (assessed using Corrected Hit Rate and EC50 values) and Consummatory Taste Reward test will be taken at Visits 3, 5 & 8.
- Fasting Bloods: Glucose, Insulin, GLP-1, PYY, FGF19, Acyl & Desacyl ghrelin, Plasma, Bile Acids, DNA, RNA at Visits 3, 5, 8, 10 & 14.
- Urine and Faecal samples at Visits 3, 5, 8, 10 & 14.
- RNA & DNA analysis of Stomach and Duodenum biopsies taken at Visits 4 & 11.

- Bio-electrical Impedance: %age body fat, total fat and fat-free mass at Visits 3, 5, 8, 10 & 14.

<u>In addition to the above, the following sub-teams will be investigating additional variables:</u>

1. *fMRI:*
    - Connectivity in salience, default, auditory & sensorimotor networks
    - Cerebral blood flow form Arterial spin labelling.
    - BOLD signals in the following fMRI tasks:
        - Food Picture Evaluation
        - Monetary Incentive Delay
        - Go-NoGo
        - Negative Emotional Reactivity
        - Grey matter density, sub-cortical volumes, brain age, image registration from T1/T2 Anatomical readings
        - White matter anisotropy & mean diffusivity from Diffusion Tensor Imaging
    - Eating & Behavioural Computerised Tasks: Delay Discounting, Leeds Food Choice and Progressive Ratio tasks will take place across Visits 3, 8, 10 & 14
    - Post-Prandial Bloods: Glucose, Insulin, GLP-1, PYY, Acyl & Desacyl ghrelin, Plasma, Bile Acids at Visits 3, 5, 8, 10.

2. *Insulin Clamps:*
    - The following analytes will be measured from Clamp Samples taken at Visits 3,5 & 8:
        - Glucose for GCMS
        - Infusates (Dextrose & Spiked Dextrose)
        - Insulin Infusate
        - Insulin
        - Glucagon
        - NEFAs
        - C-Peptide
    - UAC Ratio at visits 3, 5, 8, 10 & 14

3. *Food Preference:*
    - Eating & Behavioural Computerised Tasks: Delay Discounting, Leeds Food Choice will take place across Visits 3, 8, 10 & 14 and Progressive Ratio tasks will take place across Visits 3 & 8 only.
    - 24H Dietary Recall and EPIC FFQ will be taken at Visits 3, 5 (recall only), 8, 10 & 14

- Post-Prandial Bloods: Glucose, Insulin, GLP-1, PYY, FGF19, Acyl & Desacyl ghrelin, Plasma, Bile Acids at Visits 3, 5, 8, 10.

A detailed breakdown of the above can be found in Appendix A

6.2.3. Metabonomic Analysis

Urine, Plasma and Stool samples will be taken at Visits 3,5,8,10 and analysed for metabolite levels.

6.2.4. Cost Effectiveness

To estimate the cost-effectiveness of the EndoBarrier device over the trail period and long-term we will be studying the following:

1. Cost of interventions and related health and social care
2. QALYs accrued (calculated from area under the EQ-5D curve)
3. Incremental cost per QALY within the trial period and over the long-term

Final analysis of health economics will be performed by a health economist and will be defined in a document external to this SAP.

6.2.5. Safety Variables

To evaluate the safety of the EndoBarrier we will be investigating the following:

1. Reduction in dose/or number of medications
2. Frequency of Adverse and Serious Adverse Events over the follow-up period
3. Changes in blood and urine analyte levels over the follow-up period including;
    i. Haematology
    ii. Clinical Biochemistry
    iii. Micronutrients and Vitamins
    iv. Insulin
    v. TSH and Thyroxin
    vi. Urinalysis
4. Changes in Vital Signs

6.3. Exploratory Efficacy Analysis

In addition to the secondary efficacy analysis we will also investigate the effect of the EndoBarrier device over multiple time points. Analysis will be run in comparison with conventional medical therapy, diet and exercise for the following:

- Vital Signs (Weight, Waist Circ. Blood Pressure) (V3 – V12/V15)
- Blood Glucose (Absolute) (V3 – V12/V15, excluding V4)
- Blood Glucose (Low/High)* (V3 – V12/V15, excluding V4)
- Insulin (V3, V5, V8, V10, V14)
- Fasting Lipids (Cholesterol, HDL, LDL, Triglycerides), (V3 – V12/V15, excluding V4 & V11)
- Fasting Liver Function Tests (ALT, AST, ALP, Urea), (V3 – V12/V15, excluding V4 & V11)

* Blood Glucose will be dichotomised into low/high groups based on the cut-off point of 82 mg/dl

7. Statistical Methodology

7.1. Baseline Demographics

Patient characteristics will be summarized. Summaries of continuous variables will be presented as means and standard deviations if normally distributed, and as medians and inter-quartile ranges for skewed data, whilst categorical variables will be presented as frequencies and percentages.

7.2. Primary Analysis

The difference between the two study groups in the proportion of patients achieving substantial improvement in the metabolic syndrome at 12 months will be analysed using logistic regression adjusting for the stratification variables (BMI groups and sites).

All statistical tests will be two-tailed with a 5% significance level.

A sensitivity analysis to take missing data due to subject withdrawal into account will be carried out. Details will be provided in Section 7.6.

7.3. Secondary Analysis

Analysis of secondary outcomes will be conducted using standard statistical procedures applicable to categorical or continuous data as deemed appropriate within the following sections:

7.3.1. Secondary Efficacy Analysis:

All statistical tests will be carried out as described below; two-tailed (where appropriate) and with a 5% significance level.

The difference between the two study groups in achieving the following secondary outcome variables will be analysed using logistic regression adjusting for the stratification variables (BMI groups and sites).

1. Proportion of patients achieving HbA1c levels of less than 6% (or 42 mmol/mol)
2. Proportion of patients achieving blood pressure values below 135/85 mmHg
3. Proportion of patients achieving absolute weight loss greater than 15% body fat content.

Tables detailing summary statistics of the above, including change from baseline, will also be provided.

7.3.2. Mechanistic Sub-Group Analysis

Mechanistic Analytes defined in section 6.2.2 and Appendix A will be assessed using a mixed model:

$$Y_{ijk} = \mu + \pi_j + \tau_i + CV_1 + \ldots + CV_r + (\pi_{j*} \tau_i) + b_{j(k)} + e_{ijk}$$

In this model:

- *μ is the intercept of the model;*
- *τ_i is the ith fixed treatment, i = 0 (standard therapy) or = 1 (EndoBarrier);*
- *π_j is the fixed visit effect at j months where j = 1,...,12 ;*
- *$CV_1 + \ldots + CV_r$ is the fixed effect of Covariates* 1 to r*
- *$b_{j(k)}$ is the random visit effect at the jth visit month of the kth subject;*
- *e_{ijk} is the random error associated with the kth subject receiving treatment i at visit j ;*
- *$b_{j(k)}$ and e_{ijk} are independent for i = 2,1 , j =1,...,5 , and k =1,..., n .*

**Covariates will include: Age, Gender*

Where repeated measure variables are nested (for example, where multiple blood samples are taken per visit), an additional fixed effect (and subsequent interaction terms) will be included to account for the nested variable. Due to the small size of the sub-groups, an additional random effect will not be included in order to keep the nested model as simple as possible. An unstructured covariate structure will be used. In the even where convergence has not been met, an alternative structure will be used as appropriate.

Analysis will be presented in the form of test results of fixed effects and estimates of model parameters. Post-hoc testing via least square means may also be performed on any model parameters with a p-value of $p<0.05$.

Additional testing will be performed within the following mechanistic sub-group as detailed below:

- *Food preference:*

Regressions will be performed with clinical outcomes (i.e. BMI, glucose control) to identify predictive markers and generate mechanistic hypotheses.

- *fMRI Tests:*

fMRI studies analysis will use region of interest analyses (e.g. for food pictures orbitofrontal cortex (OFC), amygdala, caudate, nucleus accumbens and anterior insula; for MID task nucleus accumbens, dorsal striatum and OFC; for Go-NoGo task pre-supplementary motor area). ANCOVA may be used in place of the mixed model, adjusting for the relevant co-variates as data is only collected at one timepoint post baseline.

- *Insulin Clamps:*

Overall and tissue specific insulin sensitivity will be quantified for each patient and compared using the mixed model defined above. Regressions may be performed with clinical outcomes (i.e. BMI, glucose control) to identify predictive markers and generate mechanistic hypotheses.

7.3.3. Metabonomic Analysis

Metabolic datasets will be analysed using principal component analysis (PCA) and orthogonal partial least-squares analysis (O-PLS). The metabolic and microbial data will also be analysed in relation to response measurements such as BMI, gut hormone levels and etc. using O-PLS regression analysis and Bayesian approaches. A range of statistical methods will be optimised and applied to the data to identify weight loss and T2DM-associated microbiota and metabolites.

7.3.4. Exploratory Efficacy Analysis

The absolute change from baseline over time for variables listed in Section 6.2.1.2 will be will be analysed using a linear mixed model based upon a repeated-measures design.

$$Y_{ijk} = \mu + \pi_j + \tau_i + CV_1 + \ldots + CV_r + (\pi_{j*} \tau_i) + b_{j(k)} + e_{ijk}$$

In this model:

- *μ is the intercept of the model;*
- *τ_i is the ith fixed treatment, i = 0 (standard therapy) or = 1 (EndoBarrier);*
- *π_j is the fixed visit effect at j months where j = 1,...,12 ;*
- *$CV_1 + \ldots + CV_r$ is the fixed effect of baseline covariates* 1 to r*
- *$b_{j(k)}$ is the random visit effect at the jth visit month of the kth subject;*
- *e_{ijk} is the random error associated with the kth subject receiving treatment i at visit j ;*
- *$b_{j(k)}$ and e_{ijk} are independent for i = 2,1 , j =1,...,5 , and k =1,..., n .*

**Baseline covariates will include: Age, Gender, BMI Group*

A plot showing mean change (± 95% CI) over time will also be produced for all continuous variables.

Analysis will be presented in the form of test results of fixed effects and estimates of model parameters. Post-hoc testing via least square means may also be performed on any model parameters with a p-value of $p<0.05$.

7.3.5. Cost Effectiveness

A health economist is to analyse selected data to determine the overall economic benefit of the EndoBarrier device. Details of this analysis will be covered in a separate document, external from the SAP.

7.4. Safety Analysis

Adverse events will be summarised by treatment group with a separate summary table showing severity and relation to study. Serious adverse events will be listed and summarised by site and category with a separate summary table showing severity and relation to study.

Blood and urine analyte levels will be summarised by absolute value by change from baseline. Any clinically significant values or changes will be reported and investigated using the mixed model and least square means testing as presented in section 7.3.4.

7.5. Interim Analysis

Interim reports detailing baseline demographics, safety endpoints and primary endpoints (if requested) will be produced for assessment and discussion at DMEC meetings, the framework of which will be provided within the DMEC charter.

7.6. Missing Data and Withdrawn Subjects

The primary analysis is likelihood based and will naturally handle missing outcome data (substantial improvement in the metabolic syndrome at 12 months) under the assumption that data are Missing-At-Random (MAR), conditional on treatment group and stratification factors (BMI groups and sites). Based on the pattern and extent of missingness, if appropriate we will explore the effect of alternative missing data assumptions on the primary analysis within sensitivity analysis using multiple imputation by chained equations (MICE). Where applicable, we will assume that the probability of missing data is not dependant on the values of the unobserved data and that the data is Missing-At-Random (MAR), conditional on treatment group and stratification factors (BMI groups and sites) as well as on HBa1c values at timepoints M3, M6, M9 and M12. A total of 20 Imputed data sets will be drawn separately for each randomised group, replace missing outcome values with simulated values from a set of imputation models containing BMI group, sites, HBa1c values at M3, M6, M9 and M12. Using MICE, missing values for the binary outcome will be imputed using a binary logistic model, including all other covariates. Missing values for any of the continuous interim HBa1c included in the imputation model will be imputed using linear regression models.

Parameter estimates across the iterations will be combined using Rubin's rules and subsequently analysed using the same methodology as the primary analysis described in Section 7.2.

Alongside MICE, we will also examine the difference in proportion of substantial improvement amongst those missing and those observed to obtain an alternative result from that concluded from the complete case analysis difference between the two arms. Four scenarios are to be considered:

1. Missing participants within the EndoBarrier arm to have an increased rate of substantial improvement. Missing participants within the standardised treatment arm to have the same rate of substantial improvement.
2. Missing participants within the standardised treatment arm to have an increased rate of substantial improvement. Missing participants within the EndoBarrier arm to have the same rate of substantial improvement.
3. Missing participants within the EndoBarrier arm to have a lower rate of substantial improvement. Missing participants within the standardised treatment arm to have the same rate of substantial improvement.
4. Missing participants within the standardised treatment arm to have a lower rate of substantial improvement. Missing participants within the EndoBarrier arm to have the same rate of substantial improvement.

The value of the proportion required within the missing data to affect the result for each scenario will be reported

All withdrawn subjects will be considered for analysis according to the intention to treat principle. Additional subjects may be recruited to allow for any randomised subjects who have withdrawn prior to commencing treatment (Visit 4). Any subjects withdrawn during this period will still be required to attend the end-of-study follow-up visit.

8. Tables to Present

8.1. Subject Disposition

Table 1.1: Subject Disposition

		Site A	Site B	Total
**Screened**				
**Randomised**				
**Treatment A**	**BMI 30-40**			
	**BMI 40-50**			
**Treatment B**	**BMI 30-40**			
	**BMI 40-50**			
**Withdrawn**				
Reason for Withdrawal				
**Completed**				

8.2. Protocol Deviations & Violations

Table 2.1: Number of protocol deviations by centre and category

Type of Deviation	Site A	Site B	Total
Patient was incorrectly included in the trial (did not meet all the inclusion and exclusion criteria			
⋮			
Patient pregnancy			
Other			
**Total**			

8.3. Baseline Demographics

Table 3.1: Summary of Baseline Demographics

Variable	Statistics	Control	Treatment
Age (y)	n		
	Mean		
	SD		
Ethnicity (n (%))	Asian		
	…		
	White		
Race (n (%))	Indian		
	…		
	White Other		
Gender (n (%))	Female		
	Male		

Variable	Statistics	Control	Treatment
Height (cm)	n		
	Mean		
	SD		
Weight (kg)	n		
	Mean		
	SD		
BMI	n		
	Mean		
	SD		
BMI Strata (n (%))	30-40 kg/m2		
	40-50 kg/m2		
Pulse	n		
	Mean		
	SD		
Systolic BP	n		
	Mean		
	SD		
Diastolic BP	n		
	Mean		
	SD		
HbA1c	n		
	Mean		
	SD		

8.4. Primary Endpoint

Table 4.1.1: **Summary of Reduction in Percentage of HbA1c Values over Time**

Laboratory Test (units)	Treatment	Visit	Timepoint	Samples Taken	n	Mean	SD	Median	Min.	Max.
HbA1c (mmol/mol)	Control	V11	M12							
		V13	M18							
		V15	M24							
	Treatment	V11	M12							
		V13	M18							
		V15	M24							

Table 4.1.2: **Proportion of Subjects Achieving a Reduction of HBA1C of 20% at 12 months***

Treatment	Samples Taken	Number Subjects Breaking 20% Threshold (n (%))
Control		
Treatment		

Table 4.2.1: Table Showing **Logistic Regression Model of Proportion of subjects with successful reduction in HBA1C levels at 12 months* – Analysis of Effects***

Effect	DF	Chi^2	Pr > Chi^2
Treatment			
Site			
BMI			
…			
Interaction Parameters (If required)			

Table 4.2.2: Table Showing **Logistic Regression Model of Proportion of subjects with successful reduction in HBA1C levels at 12 months* – Analysis of Maximum Likelihood Estimates**

Parameter	Value	DF	Estimate	Error	Chi^2	Pr > Chi^2	Exp(Est)
Treatment	Control						
	Treatment						
Site	ICL						
	UTS						
BMI	30-40						
	40-50						
…							

Table 4.2.3: Table Showing **Logistic Regression Model of Proportion of subjects with successful reduction in HBA1C levels at 12 months* – Odds Ratio Estimates**

Effect	Point Estimate	Confidence Limits	
…			

*Note – Additional tables may be produced replicating the primary analysis for additional timepoints at Month 18 and Month 24

8.5. Secondary Endpoints

Table 5.1.1: **Summary of HbA1c Values**

Laboratory Test (units)	Treatment	Visit	Samples Taken	n	Mean	SD	Median	Min.	Max.
HbA1c (mmol/mol)	Control								
	Treatment								

Note: Summary of Percentage Reduction of HBA1C can be found in Table 4.1.1

Table 5.1.2: **Proportion of Subjects with a HBA1C level of less than 6% (or 42mmol/mol)**

Treatment	Visit	Samples Taken	Number Subjects with a HBA1C level of less than 6% (or 42mmol/mol)
Control			
Treatment			

Table 5.1.3: ***Significance test for the above***

Effect	DF	SS	MS	F Value	Pr > F
Model					
Error					
Corrected Total					

Table 5.2.1: Summary of Vital Signs

Laboratory Test* (units)	Treatment	Visit	n	Mean	SD	Median	Min.	Max.
BMI (kg/m2)	Control	V2 C						
		…						
		V9						
	Treatment	V2 RX						
		…						
		V9						

***** *To include Weight, BMI, Waist Circumference, Blood Pressure, Pulse*

Table 5.2.2: Proportion of Subjects that have reached secondary endpoint for blood pressure and/or weightloss.

Treatment	Visit	Samples Taken	Number Subjects with BP < 135/85 mmHg (n(%))	Number Subjects with >15 % Abs Weight Loss (n(%))
Control				
Treatment				

Table 5.2.3: ***Significance test for the above***

Effect	DF	SS	MS	F Value	Pr > F
Model					
Error					
Corrected Total					

Table 5.3.1: Frequency of subjects change in medication

Variable	Statistics	Control	Treatment
Change in Medication	n (%)		
	n (%)		
	n (%)		
	n (%)		

Table 5.4.1: Summary of Adverse Events

Treatment	Total Subjects Treated	Total Subjects with AEs	No. Adverse Events	Total Subjects with SAEs	No. Serious AEs
Treatment					
Control					
**All**					

Table 5.4.2: Number of Adverse Events by severity and causality relationship to study treatment

			Number of Subjects with AEs					
Treatment	Total Subjects Treated	Severity	Unrelated	Unlikely	Possible	Probable	Definite	Total
Treatment		Mild						
		Moderate						
		Severe						
Control		Mild						
		Moderate						
		Severe						
All		Mild						
		Moderate						
		Severe						
			Total Number of AEs					
	Total Subjects Treated	Severity	Unrelated	Unlikely	Possible	Probable	Definite	Total
Treatment		Mild						
		Moderate						
		Severe						
Control		Mild						
		Moderate						
		Severe						
All		Mild						
		Moderate						
		Severe						

Table 5.5.1: Summary of Serious Adverse Events by Site, Category and Treatment

		Treatment			Testing
**Site**	**Category**	**Control**	**Treatment**	**All subjects**	**P-Value**
Imperial College London					
	**Site Total**				
University Trust Southampton					
	**Site Total**				
All					
	**Total**				

Table 5.5.2: Number of Serious Adverse Events by severity and causality relationship to study treatment

			Number of Subjects with SAEs					
**Treatment**	**Total Subjects Treated**	**Severity**	**Unrelated**	**Unlikely**	**Possible**	**Probable**	**Definite**	**Total**
Treatment		**Mild**						
		**Moderate**						
		**Severe**						
Control		**Mild**						
		**Moderate**						
		**Severe**						
All		**Mild**						
		**Moderate**						
		**Severe**						
			**Total Number of SAEs**					
	**Total Subjects Treated**	**Severity**	**Unrelated**	**Unlikely**	**Possible**	**Probable**	**Definite**	**Total**
Treatment		**Mild**						
		**Moderate**						
		**Severe**						
Control		**Mild**						
		**Moderate**						
		**Severe**						
All		**Mild**						
		**Moderate**						
		**Severe**						

8.6. Mechanistic Endpoints

Note that all graphical output shown below are examples based on the type of data & analysis model used and are subject to change.

8.6.1. All Subgroups

Tables 6.1.01a – 6.1.18a: ***Mixed Model summary for Questionnaire Results****

Mixed Model Output will be displayed in full as per Appendix B

**DEBQ (6.1.01), EDE-Q (6.1.02), BIS/BAS (6.1.03), BDI-II (6.1.04), Barratt Impulsivity Scale & UPPS-P (6.1.05), Fagerstrom Nicotine Dependence Scale (6.1.06), TFEQ (6.1.07), IPAQ (6.1.08), Yale Food Addiction Scale (6.1.09), AUDIT (6.1.10), Power of Food (6.1.11), PSQI (6.1.12), Dumping Syndrome (6.1.13), HADS (6.1.14), SF36 (6.1.15), PANAS (6.1.16), Barratt Impulsivity Score (6.1.17), EPQR (6.1.18).*

Tables 6.1.01b – 6.1.18b: ***Graphical output for Questionnaire Results****

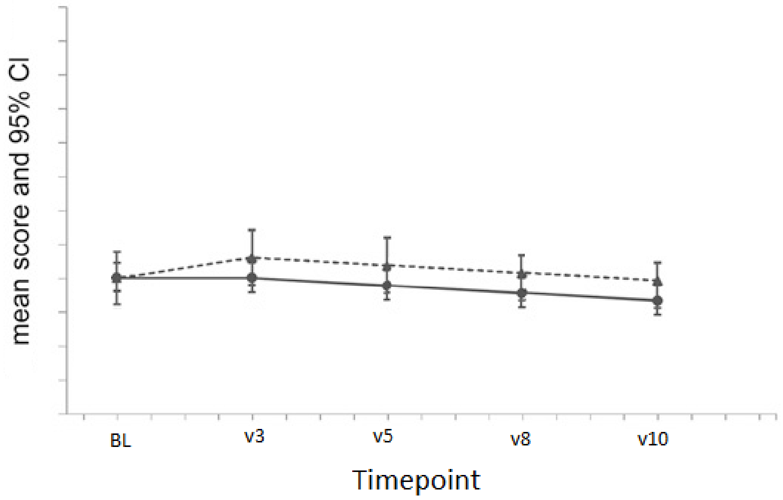

**DEBQ (6.1.01), EDE-Q (6.1.02), BIS/BAS (6.1.03), BDI-II (6.1.04), Barratt Impulsivity Scale & UPPS-P (6.1.05), Fagerstrom Nicotine Dependence Scale (6.1.06), TFEQ (6.1.07), IPAQ (6.1.08), Yale Food Addiction Scale (6.1.09), AUDIT (6.1.10), Power of Food (6.1.11), PSQI (6.1.12), Dumping Syndrome (6.1.13), HADS (6.1.14), SF36 (6.1.15), PANAS (6.1.16), Barratt Impulsivity Score (6.1.17), EPQR (6.1.18).*

Tables 6.2.01a – 6.2.07a: ***Mixed-model output for VAR Scale Results****

Mixed Model Output will be displayed in full as per Appendix B

*<u>*Hunger & Fullness (6.2.01), Nausea (6.2.02), Pleasantness-to-Eat (6.2.03), Vol. to Eat (6.2.04), Sleepiness (6.2.05), Anxiety (6.2.06), Stress (6.2.07)</u>*

Tables 6.2.01b – 6.2.07b: ***Graphical output for VAR Scale Results****

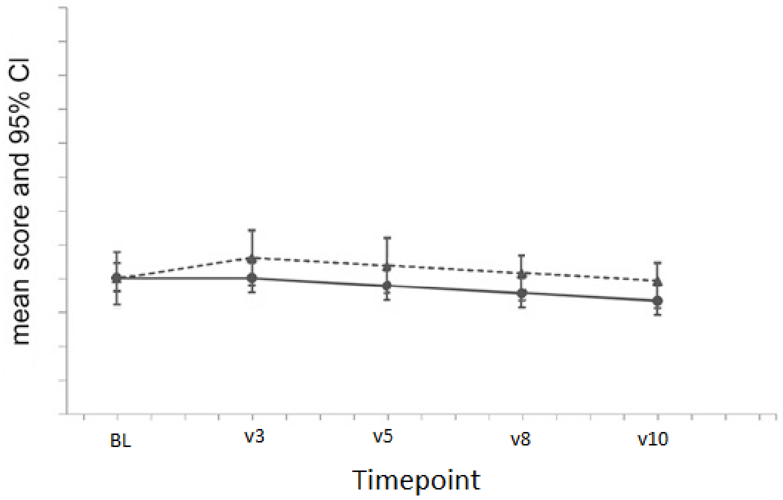

*<u>*Hunger & Fullness (6.2.01), Nausea (6.2.02), Pleasantness-to-Eat (6.2.03), Vol. to Eat (6.2.04), Sleepiness (6.2.05), Anxiety (6.2.06), Stress (6.2.07)</u>*

Tables 6.3.01a: Mixed Model Output ***for Three-Days Food Diary Test Results****

Mixed Model Output will be displayed in full as per Appendix B

*<u>*Three-Days Food Diary (6.4.03)</u>*

Tables 6.3.01b-: ***Graphical output for Three-Days Food Diary Test Results ****

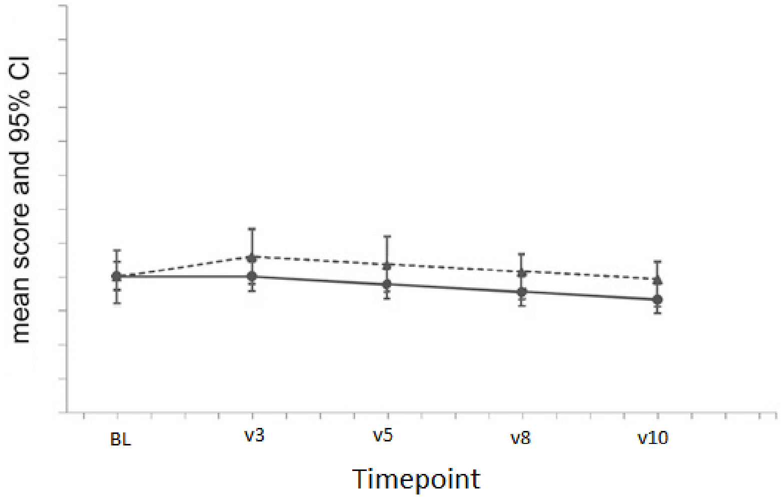

*<u>*Three-Days Food Diary (6.4.03)</u>*

Tables 6.4.01a – 6.4.02a: ***Mixed-model output for Food Preference Testing Results****

Mixed Model Output will be displayed in full as per Appendix B

<u> Sweet Taste Detection (6.4.01) and Consummatory Taste Reward test (6.5.02)</u>*

Tables 6.4.01b – 6.4.02b: ***Graphical output for Food Preference Testing Results****

<u>Sweet Taste Detection (6.4.01) – Corrected Hit Rate and EC50</u>

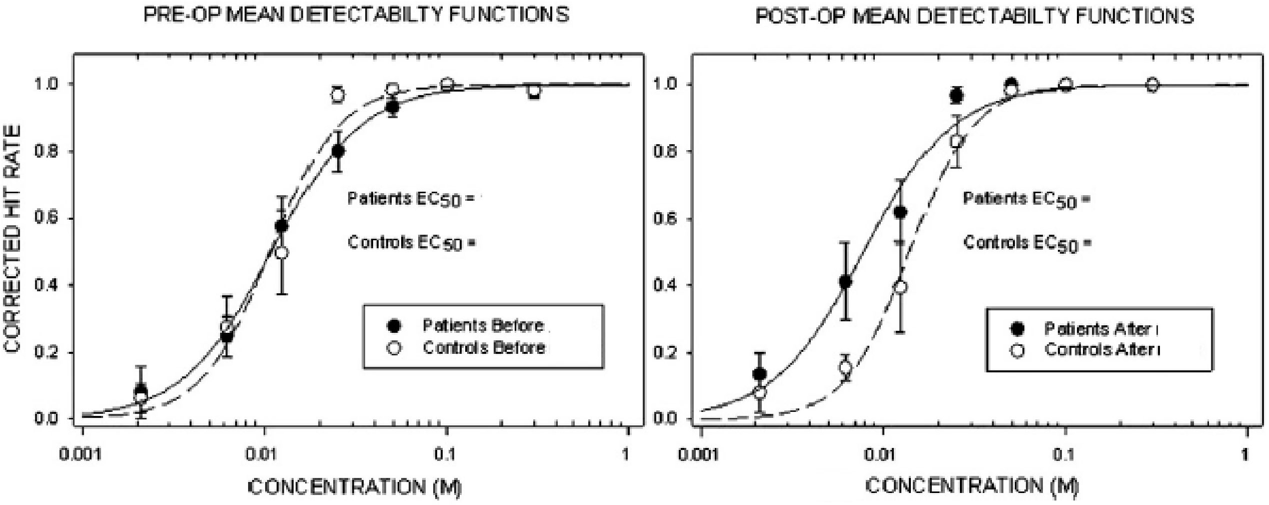

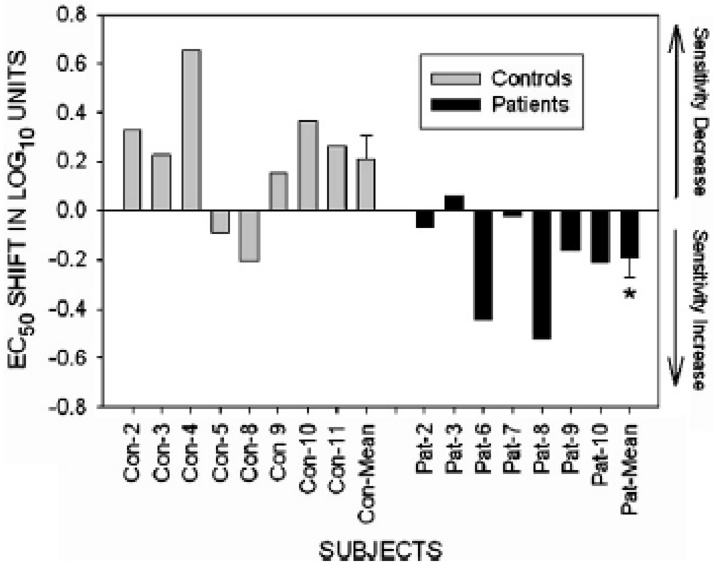

Consummatory Taste Reward test (6.4.02)

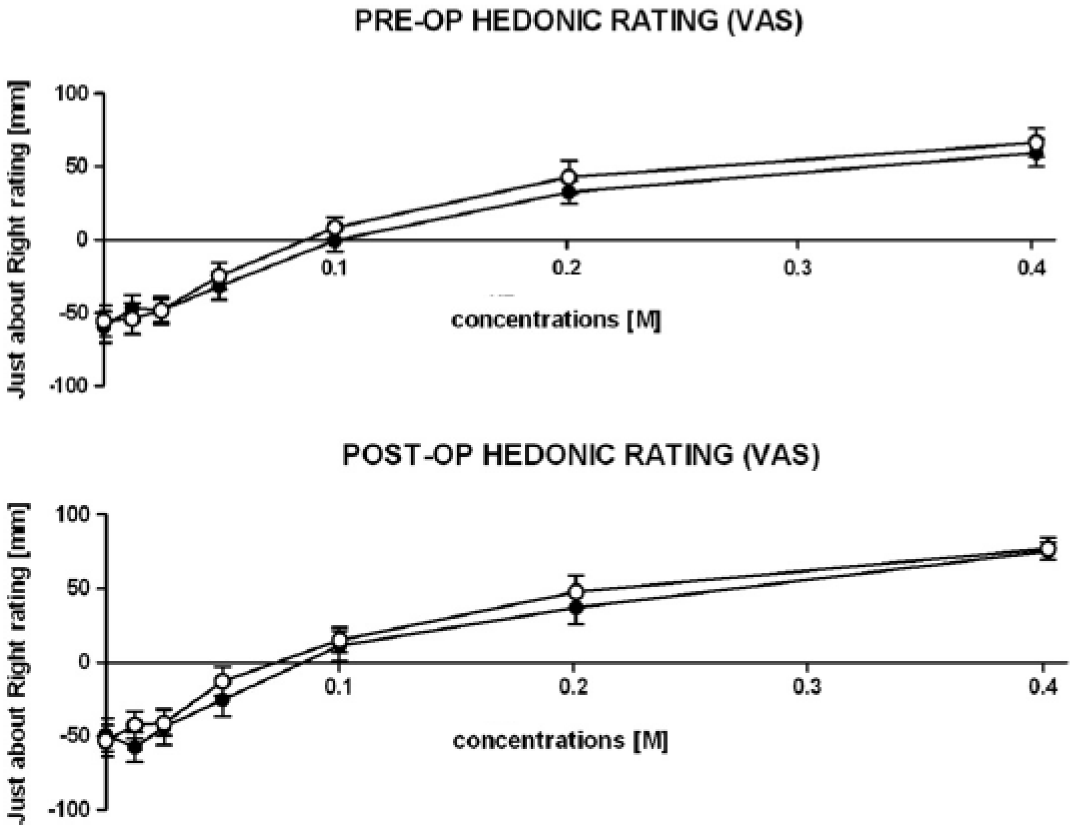

Tables 6.5.01a – 6.5.09a: ***Mixed Model Output for Fasting Blood Test Analytes****

Mixed Model Output will be displayed in full as per Appendix B

** Glucose (6.5.01), Insulin (6.5.02), GLP-1 (6.5.03), PYY (6.5.04), Acyl & Desacyl ghrelin (6.5.05), Plasma (6.5.06), Bile Acids (6.5.07), DNA (6.5.08), RNA (6.5.09)*

Tables 6.6.01b – 6.6.09b: ***Graphical output for Fasting Blood Test Analytes ****

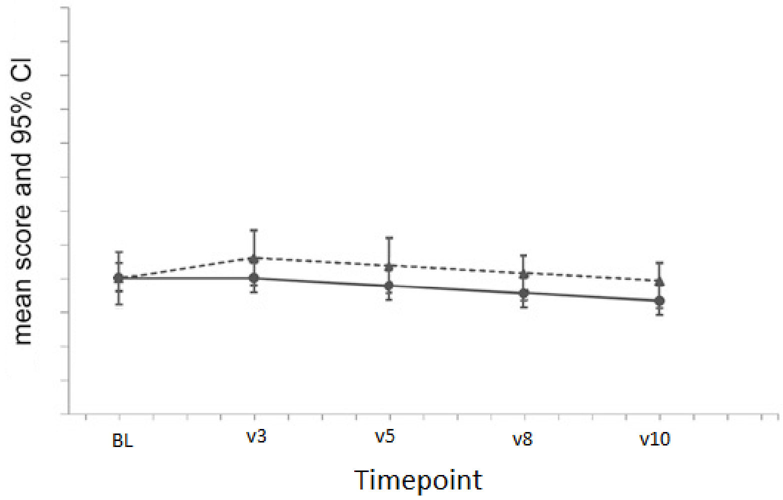

** Glucose (6.5.01), Insulin (6.5.02), GLP-1 (6.5.03), PYY (6.5.04), Acyl & Desacyl ghrelin (6.5.05), Plasma (6.5.06), Bile Acids (6.5.07), DNA (6.5.08), RNA (6.5.09)*

Tables 6.6.01a – 6.6.03a: ***Mixed Model output for Urine & Faecal Test Analytes****

Mixed Model Output will be displayed in full as per Appendix B

<u> Urine Analytes (6.6.01), Faecal Analytes (6.6.02), Gut Microbiome (6.6.03)</u>*

Tables 6.6.01b – 6.6.03b: ***Graphical output for Urine & Faecal Test Analytes ****

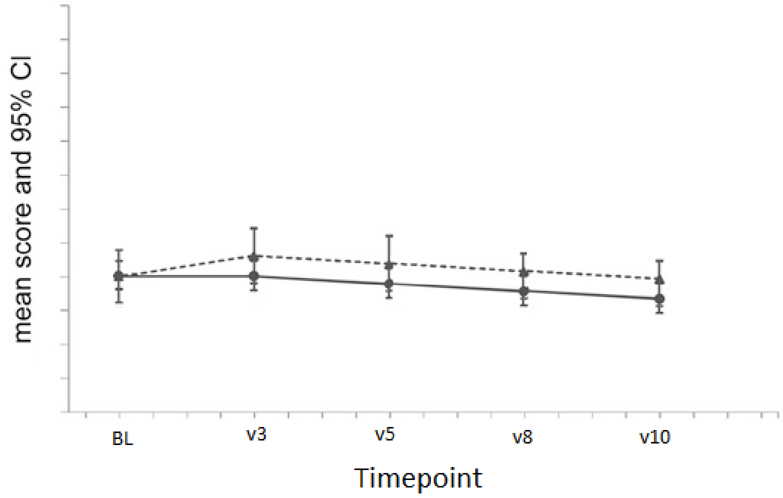

<u> Urine Analytes (6.6.01), Faecal Analytes (6.6.02), Gut Microbiome (6.6.03)</u>*

Tables 6.7.01a – 6.7.03a: ***Mixed Model output for Bio-electrical Impedance Analysis****

Mixed Model Output will be displayed in full as per Appendix B

<u> %age Body Fat (6.7.01), Fat & Fat-Free Mass (6.7.02) & (6.7.03)</u>*

Tables 6.7.01b – 6.7.03b: ***Graphical output for Bio-electrical Impedance Analysis****

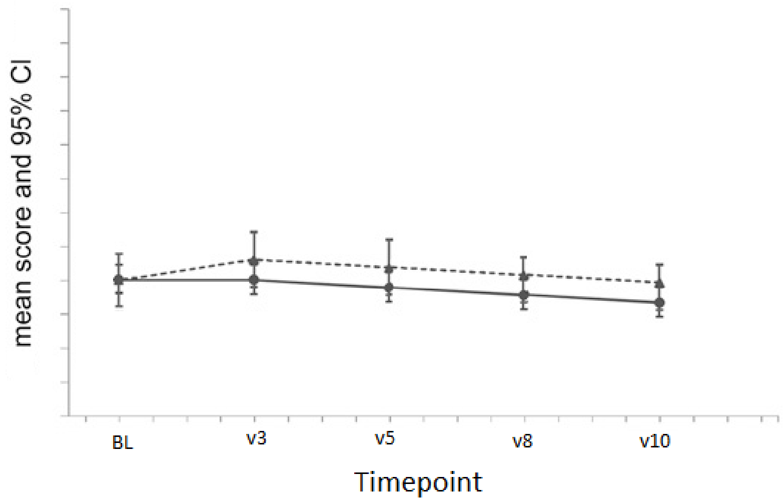

<u> %age Body Fat (6.7.01), Fat & Fat-Free Mass (6.7.02) & (6.9.03)</u>*

Tables 6.8.01: Output for ***Biopsy results for RNA & DNA analysis:***

Effect	DF	SS	MS	F Value	Pr > F
Model					
Error					
Corrected Total					

Source	DF	SS	MS	F Value	Pr > F
Treatment					
Timepoint					
Treatment*Timepoint					

8.6.2 Additional Mechanistic Output for Food Preference Subgroup

Tables 6.9.01a: ***Mixed-model output for Breakpoint Computerised Tasks***

Mixed Model Output will be displayed in full as per Appendix B

Tables 6.9.02a – 6.9.03a: ***Fisher's Chi-Squared output for Delay Discounting & Leeds Food Choice Computerised Tasks ****

Group / Treament	Timepoint			Total
xxxx - Control				
xxxx - Treatment				
…..				
**Total**				

Fisher's Exact Test	
Table Probability	
Pr <= P	

** Delay Discounting Results (6.9.02), Leeds Food Choice Results (6.9.03)*

Tables 6.9.01b : ***Graphical output for Breakpoint Computerised Tasks***

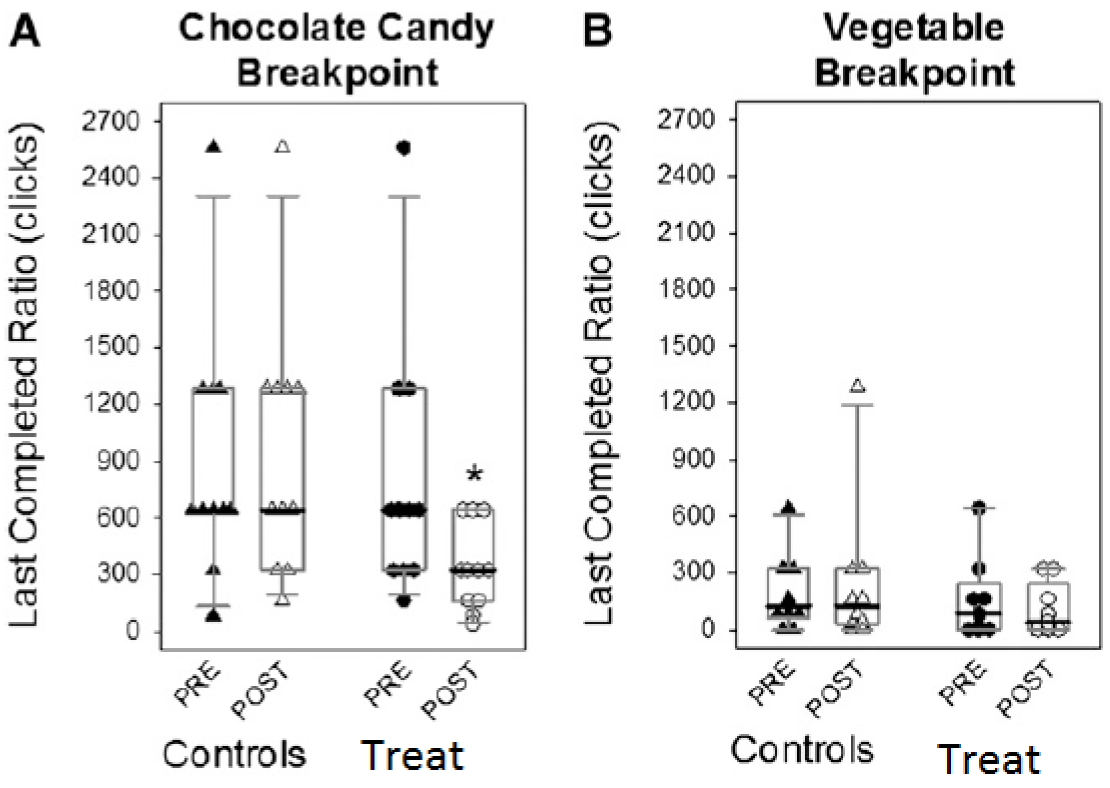

Tables 6.9.02b: ***Graphical output for Delay Discounting Computerised Tasks***

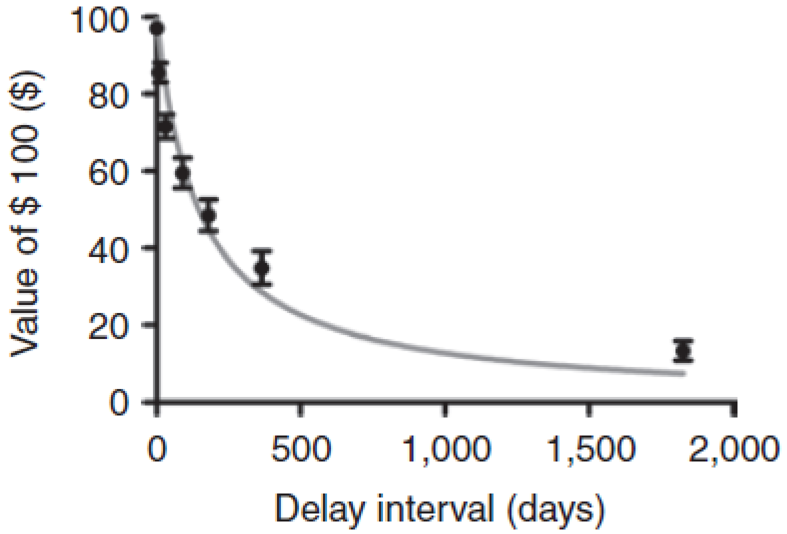

Tables 6.9.03b: ***Tabular & Graphical Output for Leeds Food Choice Results***

Item (category)	Visit A	Visit B	R	sSLT	sMWT
Spaghetti in sauce (LFSA)	11.8 (0.8)	11.3 (0.9)	.703*	10.8 (0.9)	12.3 (0.8)
Blueberry muffin (HFSW)	10.6 (1.0)	10.6 (0.9)	.610*	10.9 (0.9)	10.3 (1.0)
Pilaf rice (LFSA)	10.5 (1.1)	9.9 (1.2)	.634*	9.6 (1.2)	10.8 (1.1)
Fruit salad (LFSW)	9.9 (1.2)	10.3 (0.6)	.452	10.1 (0.6)	10.1 (0.8)

* Indicates significance at the 0.05 level.

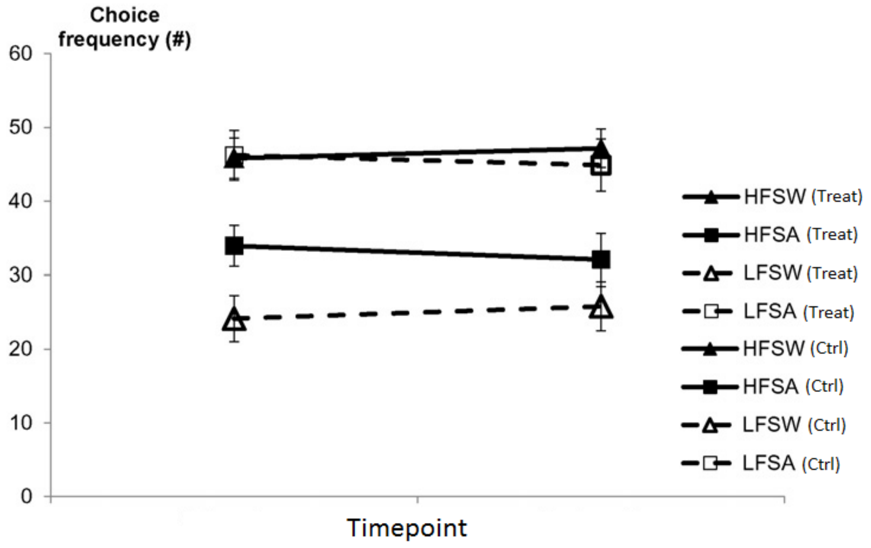

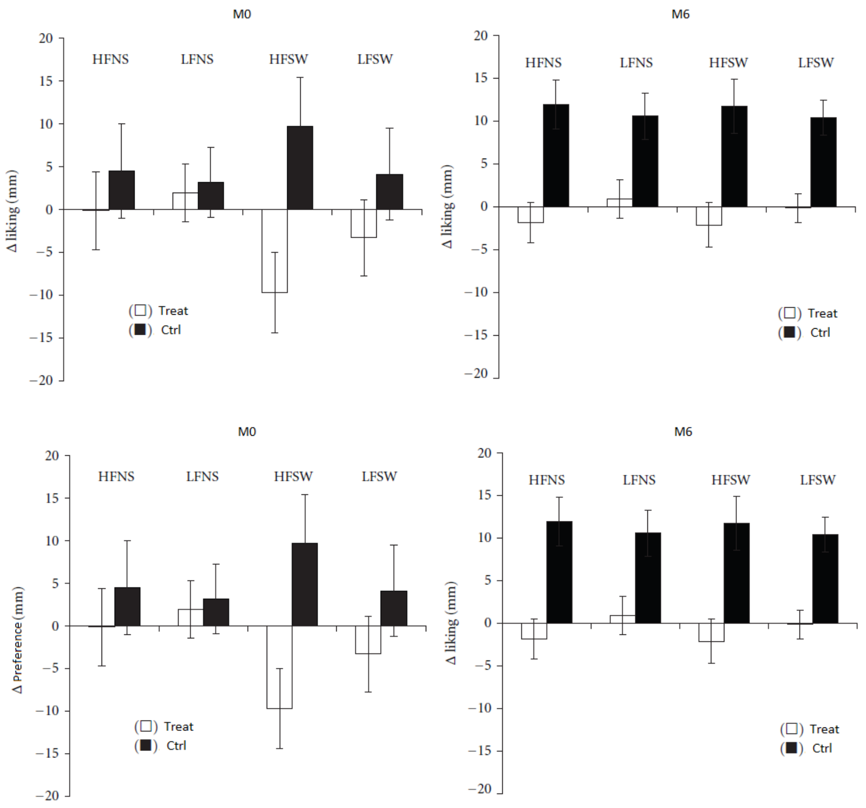

Tables 6.10.01a: Mixed Model Output ***for Food Testing Results****

Mixed Model Output will be displayed in full as per Appendix B

**EPIC FFQ (6.10.02), Three-Days Food Diary (6.10.03)*

Tables 6.10.01b-: ***Graphical output for Food Testing Results****

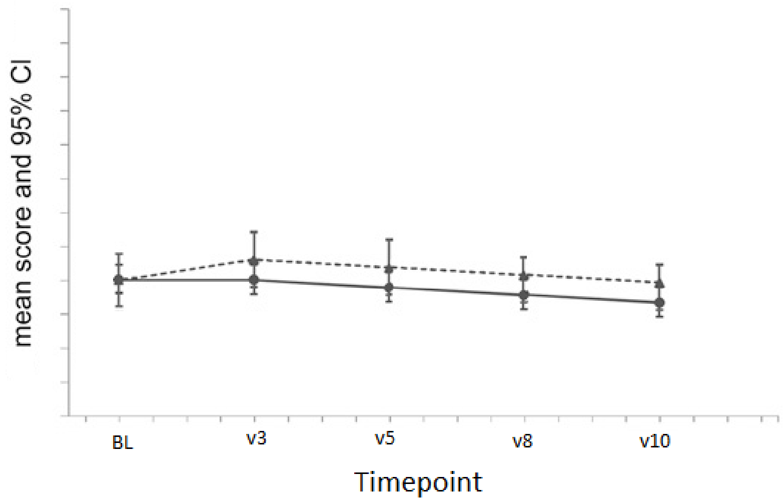

**24h Dietary Recall (6.10.01), EPIC FFQ (6.10.02)*

Tables 6.11.01a – 6.11.07a: ***ANOVA output for Post-Prandial Blood Test Analytes****

Effect	DF	SS	MS	F Value	Pr > F
Model					
Error					
Corrected Total					

Source	DF	SS	MS	F Value	Pr > F
Treatment					
Timepoint					
Treatment*Timepoint					

** Glucose (6.11.01), Insulin (6.11.02), GLP-1 (6.11.03), PYY (6.11.04), Acyl & Desacyl ghrelin (6.11.05), Plasma (6.11.06), Bile Acids (6.11.07)*

Tables 6.11.01b – 6.11.07b: ***Graphical output for Post-Prandial Blood Test Analytes ****

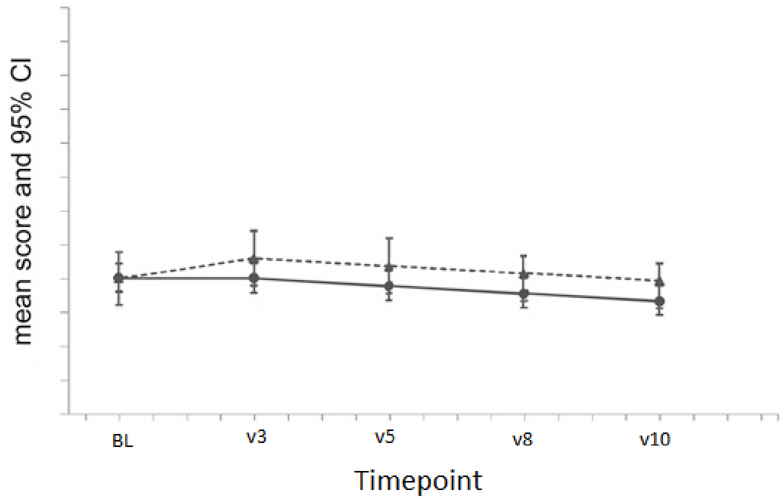

** Glucose (6.11.01), Insulin (6.11.02), GLP-1 (6.11.03), PYY (6.11.04), Acyl & Desacyl ghrelin (6.11.05), Plasma (6.11.06), Bile Acids (6.11.07)*

8.6.3 Additional Mechanistic Output for FMRI Subgroup

Tables 6.12.01a: ***Mixed-model output for Breakpoint Computerised Tasks***

Mixed Model Output will be displayed in full as per Appendix B

Tables 6.12.02a – 6.12.03a: ***Fisher's Chi-Squared output for Delay Discounting & Leeds Food Choice Computerised Tasks ****

Group / Treament	Timepoint			Total
xxxx - Control				
xxxx - Treatment				
…..				
**Total**				

Fisher's Exact Test	
Table Probability	
Pr <= P	

** Delay Discounting Results (6.12.02), Leeds Food Choice Results (6.12.03)*

Tables 6.13.01a – 6.13.07a: ***ANOVA output for Post-Prandial Blood Test Analytes****

Effect	DF	SS	MS	F Value	Pr > F
Model					
Error					
Corrected Total					

Source	DF	SS	MS	F Value	Pr > F
Treatment					
Timepoint					
Treatment*Timepoint					

<u> Glucose (6.13.01), Insulin (6.13.02), GLP-1 (6.13.03), PYY (6.13.04), Acyl & Desacyl ghrelin (6.13.05), Plasma (6.13.06), Bile Acids (6.13.07)</u>*

Tables 6.13.01b – 6.13.07b: ***Graphical output for Post-Prandial Blood Test Analytes ****

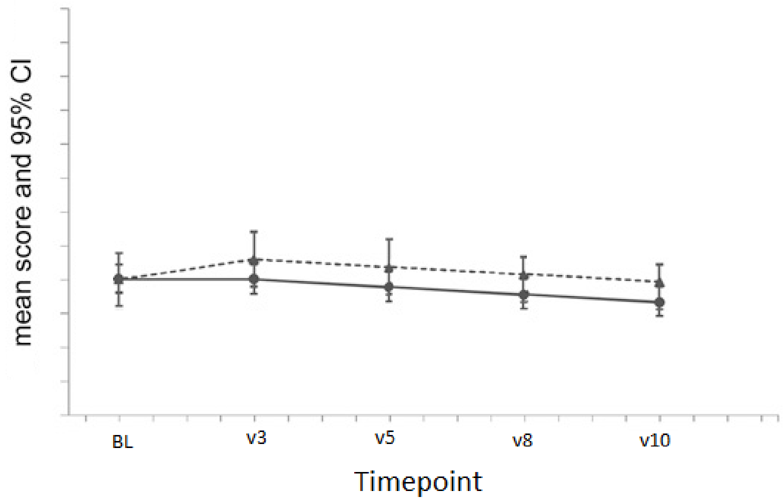

<u> Glucose (6.13.01), Insulin (6.13.02), GLP-1 (6.13.03), PYY (6.13.04), Acyl & Desacyl ghrelin (6.13.05), Plasma (6.13.06), Bile Acids (6.13.07)</u>*

Output for the following:

Tables 6.14.XX - Connectivity in salience, default, auditory & sensorimotor networks

Tables 6.15.XX - Cerebral blood flow form Arterial spin labelling.

Tables 6.16.XX - BOLD signals in the following fMRI tasks:

- Food Picture Evaluation
- Monetary Incentive Delay
- Go-NoGo
- Negative Emotional Reactivity
- Grey matter density, sub-cortical volumes, brain age, image registration from T1/T2 Anatomical readings
- White matter anisotropy & mean diffusivity from Diffusion Tensor Imaging

8.6.4 Additional Mechanistic Output for the Insulin Clamp

Tables 6.17.01a: ***Mixed-model output for Insulin Sub-group Variables***

Mixed Model Output will be displayed in full as per Appendix B

<u> Glucose (6.17.01), Insulin (6.17.02), GLP-1 (6.17.03), PYY (6.17.04), Acyl & Desacyl ghrelin (6.17.05), Plasma (6.17.06), Bile Acids (6.17.07)</u>*

Tables 6.17.01b – 6.17.07b: ***Graphical output for Insulin Sub-group Variables***

*

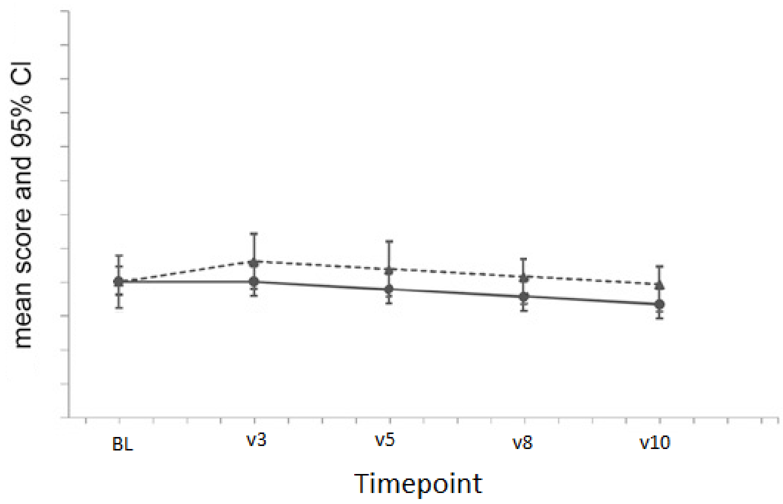

<u> Glucose (6.17.01), Insulin (6.17.02), GLP-1 (6.17.03), PYY (6.17.04), Acyl & Desacyl ghrelin (6.17.05), Plasma (6.17.06), Bile Acids (6.17.07)</u>*

8.7 Exploratory Efficacy Endpoints

Tables 7.1.01: **Summary of Exploratory Efficacy Endpoint Variables**

Laboratory Test (units)	Treatment	Visit	Samples Taken	n	Mean	SD	Median	Min.	Max.
xxx (xxxxl/xxxx)	Control								
	Treatment								

Table 7.1.02: **Summary of Change from Baseline within Exploratory Efficacy Endpoint Variables**

Laboratory Test (units)	Treatment	Visit	Samples Taken	n	Mean	SD	Median	Min.	Max.
xxx (xxxxl/xxxx)	Control								
	Treatment								

Tables 7.1.03a – 7.1.09a: ***Mixed-model output for* Exploratory Efficacy Endpoint Variables**

Mixed Model Output will be displayed in full as per Appendix B

** Glucose (7.1.01), Insulin (7.1.02), GLP-1 (7.1.03), PYY (7.1.04), Acyl & Desacyl ghrelin (7.1.05), Plasma (7.1.06), Bile Acids (7.1.07)*

Tables 7.1.03b – 7.1.09b: ***Graphical output for for Insulin Sub-group* Variables**

*****

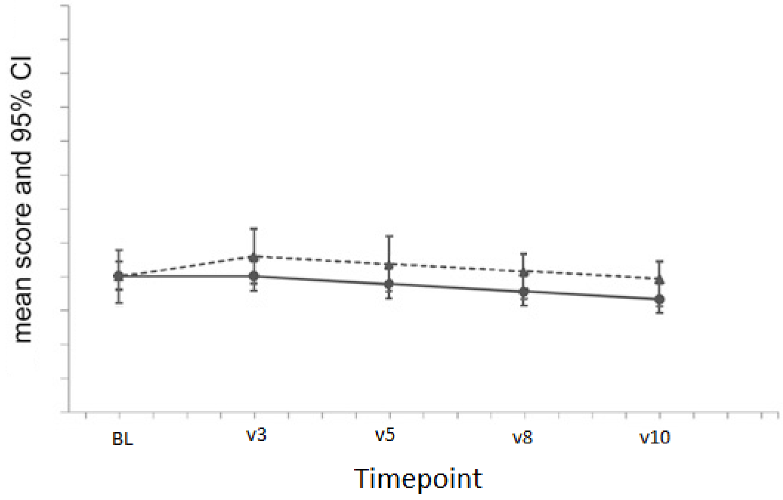

<u> Glucose (7.1.01), Insulin (7.1.02), GLP-1 (7.1.03), PYY (7.1.04), Acyl & Desacyl ghrelin (7.1.05), Plasma (7.1.06), Bile Acids (7.1.07)</u>*

8.8 Safety Endpoints

Table 8.1.01: **Summary of Haematology Values**

Laboratory Test (units)	Treatment	Visit	Samples Taken	n	Mean	SD	Median	Min.	Max.
xxx (xxxxl/xxxx)	Control								
	Treatment								

Table 8.1.02: **Summary of Change from Baseline within Haematology Values**

Laboratory Test (units)	Treatment	Visit	Samples Taken	n	Mean	SD	Median	Min.	Max.
xxx (xxxxl/xxxx)	Control								
	Treatment								

Table 8.2.01: **Summary of Clinical Biochemistry Values**

Laboratory Test (units)	Treatment	Visit	Samples Taken	n	Mean	SD	Median	Min.	Max.
xxx (xxxx/xxxx)	Control								
	Treatment								

Table 8.2.02: **Summary of Change from Baseline within Clinical Biochemistry Values**

Laboratory Test (units)	Treatment	Visit	Samples Taken	n	Mean	SD	Median	Min.	Max.
xxx (xxxx/xxxx)	Control								
	Treatment								

Table 8.3.01: **Summary of Micronutrient and Vitamin Values**

Laboratory Test (units)	Treatment	Visit	Samples Taken	n	Mean	SD	Median	Min.	Max.
xxx (xxxx/xxxx)	Control								
	Treatment								

Table 8.3.02: **Summary of Change from Baseline within Micronutrient and Vitamin Values**

Laboratory Test (units)	Treatment	Visit	Samples Taken	n	Mean	SD	Median	Min.	Max.
xxx (xxxx/xxxx)	Control								
	Treatment								

Table 8.4.01: **Summary of TSH and Thyroxin Values**

Laboratory Test (units)	Treatment	Visit	Samples Taken	n	Mean	SD	Median	Min.	Max.
xxx (xxxx/xxxx)	Control								
	Treatment								

Table 8.4.02: **Summary of Change from Baseline within TSH and Thyroxin Values**

Laboratory Test (units)	Treatment	Visit	Samples Taken	n	Mean	SD	Median	Min.	Max.
xxx (xxxx/xxxx)	Control								
	Treatment								

Table 8.5.01: **Summary of Insulin Values**

Laboratory Test (units)	Treatment	Visit	Samples Taken	n	Mean	SD	Median	Min.	Max.
xxx (xxxx/xxxx)	Control								
	Treatment								

Table 8.5.02: **Summary of Change from Baseline within Insulin Values**

Laboratory Test (units)	Treatment	Visit	Samples Taken	n	Mean	SD	Median	Min.	Max.
xxx (xxxx/xxxx)	Control								
	Treatment								

Table 8.6.01: **Summary of Fasting Lipids Values**

Laboratory Test (units)	Treatment	Visit	Samples Taken	n	Mean	SD	Median	Min.	Max.
xxx (xxxx/xxxx)	Control								
	Treatment								

Table 8.6.02: **Summary of Change from Baseline within Fasting Lipids Values**

Laboratory Test (units)	Treatment	Visit	Samples Taken	n	Mean	SD	Median	Min.	Max.
xxx (xxxx/xxxx)	Control								
	Treatment								

Table 8.7.01: **Summary of Urinalysis Values**

Variable	Statistics	Control	Treatment	All Subjects
xxx (xxxx/xxxx)	0			
	Trace			
	1+			
	2+			
	3+			
…	…			

APPENDICIES

Appendix A1: Food Preference Mechanistic Variables

Mechanistic Variable Type	Mechanistic study Variable	Outcome Variable	Sub study	Visits
**Questionniares**	Dutch Eating Behaviour Questionnaire (DEBQ)	External eating, dietary restraint, emotional eating	FP	3,8,10,14
	Eating Disorder Examination Questionnaire (EDE–Q)	Restraint, eating concern, weight concern, shape concern, global score	FP	3,8,10,14
	Behavioural Inhibition and Activation System (BIS / BAS) scales	Drive, reward responsiveness, fun seeking, behavioural inhibition	FP	3,8,10,14
	Beck Depression Inventory (BDI–II)	Depression score	FP	3,8,10,14
	Barratt Impulsivity Scale and UPPS-P	Negative urgency, premedictation, perserverance, sensation seeking, positive urgency	FP	3,8,10,14
	Fagerström Nicotine Dependence Scale	Fagerstroms nicotine dependence score	FP	3,8,10,14
	Three Factor Eating Questionnaire (TFEQ)	Disinhibition, dietary restraint, hunger	FP	3,8,10,14
	International Physical Activity Questionnaire (IPAQ)	Physical activity	FP	3,8,10,14
	Yale Food Addiction Scale	Food addiction	FP	3,8,10,14
	Alcohol Use Disorders Identification Test (AUDIT)	Alcohol dependence score	FP	3,8,10,14
	Power of Food	Power of food score	FP	3,8,10,14
	Pittsburgh Sleep Quality Index (PSQI)	Sleep quality	FP	3,8,10,14
	Dumping Syndrome	Arts, Sigstad scores	FP	3,8,10,14
	Dumping Syndrome	Arts, Sigstad scores	FP	6,7
	Hospital Anxiety and Depression Scale (HADS)	Anxiety, depression score	FP	3,8,10,14
	Short-Form 36 Health Survey Questionnaire (SF36)	Vitality, physical functioning, bodily pain, general health perceptions, physical role functioning, emotional role functioning, social role functioning, mental health	FP	3,8,10,14
	Positive and Negative Affect Schedule (PANAS)	Postive & negative affect	FP	3,8,10,14
	Barrat Impulsivity Scale	Barratt impulsivity score	FP	3,8,10,14
	EPQR	Psychoticism, neuroticism, extraversion, lying	FP	3,8,10,14
**Visual Analogue Rating Scales**	How hungry do you feel right now (Scale from 1-10)	Hunger	FP	3,5,8,10,14
	How sick do you feel right now (Scale from 1-10)	Fullness	FP	3,5,8,10,14
	How pleasant would it be to eat right now (Scale from 1-10)	Nausea	FP	3,5,8,10,14
	How anxious do you feel right now (Scale from 1-10)	Pleasantness to eat	FP	3,5,8,10,14
	How much do you think you could eat right now (Scale from 1-10)	Volume to eat	FP	3,5,8,10,14
	How feel do you feel right now (Scale from 1-10)	Sleepiness	FP	3,5,8,10,14
	How stressed do you feel right now (Scale from 1-10)	Anxiety	FP	3,5,8,10,14
	How sleepy do you feel right now (Scale from 1-10)	Stress	FP	3,5,8,10,14

Mechanistic Variable Type	Mechanistic study Variable	Outcome Variable	Sub study	Visits
**Eating and Behaviour Computerised Tasks**	Delay Discounting Task	Discounting rate (low, medium, high monetary reward)	FP	3,8,10,14
	Leeds Food Choice Task	Sugar bias, fat bias	FP	3,8,10,14
	Progressive Ratio Task	Breakpoint	FP	3,8
**Dietary**	24h Dietary Recall	Total energy intake and Energy intake by macronutrient	FP	3,5, 8,10,14
	EPIC FFQ	Total energy intake and Energy intake by macronutrient	FP	3,8,10,14
	Three-Days Food Diary	Total energy intake and Energy intake by macronutrient	FP	3,5, 8,10,14
**Food preference tasting tests**	Sweet Taste Detection	EC50 and Corrected hit rate	FP	3,5,8
	Consummatory Taste Reward	Just about right, Pleasentess, Intensity	FP	3,5,8
**Biological Samples**	Fasting bloods	Glucose	FP	3,5, 8,10,14
		Insulin	FP	3,5, 8,10,14
		GLP-1	FP	3,5, 8,10,14
		PYY	FP	3,5, 8,10,14
		Acyl & Desacyl ghrelin	FP	3,5, 8,10,14
		Plasma (stored for metabonomics)	FP	3,5, 8,10,14
		Bile Acids	FP	3,5, 8,10,14
		DNA	FP	3,5, 8,10,14
		RNA	FP	3,5, 8,10,14
	Post-prandial bloods	Glucose	FP	3,5, 8,10
		Insulin	FP	3,5, 8,10
		GLP-1	FP	3,5, 8,10
		PYY	FP	3,5, 8,10
		Acyl & Desacyl ghrelin	FP	3,5, 8,10
		Plasma (stored for metabonomics)	FP	3,5, 8,10
		Bile Acids	FP	3,5, 8,10
	Urine	Urine (stored for metabonomics)	FP	3,5, 8,10,14
	Faeces	Faeces (stored for metabonomics)	FP	3,5, 8,10,14
		Gut microbiome	FP	3,5, 8,10,14
	Biopsies	Stomach for RNA and DNA analysis	FP	4, 11
		Duodenum for RNA and DNA analysis	FP	4, 11
**Body composition**	Bio-electrical imedance analysis	% body fat	FP	3,5, 8,10,14
		Fat & fat free mass	FP	3,5, 8,10,14

Appendix A2: fMRI Mechanistic Group Variables

Mechanistic Variable Type	Mechanistic study Variable	Outcome Variable	Sub study	Visits
**Questionniares**	Dutch Eating Behaviour Questionnaire (DEBQ)	External eating, dietary restraint, emotional eating	fMRI	3,8,10,14
	Eating Disorder Examination Questionnaire (EDE−Q)	Restraint, eating concern, weight concern, shape concern, global score	fMRI	3,8,10,14
	Behavioural Inhibition and Activation System (BIS / BAS) scales	Drive, reward responsiveness, fun seeking, behavioural inhibition	fMRI	3,8,10,14
	Beck Depression Inventory (BDI−II)	Depression score	fMRI	3,8,10,14
	Barratt Impulsivity Scale and UPPS-P	Negative urgency, premedictation, perserverance, sensation seeking, positive urgency	fMRI	3,8,10,14
	Fagerström Nicotine Dependence Scale	Fagerstroms nicotine dependence score	fMRI	3,8,10,14
	Three Factor Eating Questionnaire (TFEQ)	Disinhibition, dietary restraint, hunger	fMRI	3,8,10,14
	International Physical Activity Questionnaire (IPAQ)	Physical activity	fMRI	3,8,10,14
	Yale Food Addiction Scale	Food addiction	fMRI	3,8,10,14
	Alcohol Use Disorders Identification Test (AUDIT)	Alcohol dependence score	fMRI	3,8,10,14
	Power of Food	Power of food score	fMRI	3,8,10,14
	Pittsburgh Sleep Quality Index (PSQI)	Sleep quality	fMRI	3,8,10,14
	Dumping Syndrome	Arts, Sigstad scores	fMRI	3,8,10,15
	Dumping Syndrome	Arts, Sigstad scores	FP	6,7
	Binge Eating Scale	Binge eating score	fMRI	3,8,10,14
	Hospital Anxiety and Depression Scale (HADS)	Anxiety, depression score	fMRI	3,8,10,14
	Short-Form 36 Health Survey Questionnaire (SF36)	Vitality, physical functioning, bodily pain, general health perceptions, physical role functioning, emotional role functioning, social role functioning, mental health	fMRI	3,8,10,14
	Positive and Negative Affect Schedule (PANAS)	Postive & negative affect	fMRI	3,8,10,14
	Barrat Impulsivity Scale	Barratt impulsivity score	fMRI	3,8,10,14
	EPQR	Psychoticism, neuroticism, extraversion, lying	fMRI	3,8,10,14
**Visual Analogue Rating Scales**	How hungry do you feel right now (Scale from 1-10)	Hunger	fMRI	3,5,8,10,14
	How sick do you feel right now (Scale from 1-10)	Fullness	fMRI	3,5,8,10,14
	How pleasant would it be to eat right now (Scale from 1-10)	Nausea	fMRI	3,5,8,10,14
	How anxious do you feel right now (Scale from 1-10)	Pleasantness to eat	fMRI	3,5,8,10,14
	How much do you think you could eat right now (Scale from 1-10)	Volume to eat	fMRI	3,5,8,10,14
	How feel do you feel right now (Scale from 1-10)	Sleepiness	fMRI	3,5,8,10,14
	How stressed do you feel right now (Scale from 1-10)	Anxiety	fMRI	3,5,8,10,14
	How sleepy do you feel right now (Scale from 1-10)	Stress	fMRI	3,5,8,10,14
	Ad libitum meal taste	Pleasant, wanting, sweet, creamy, just-right	fMRI	

Mechanistic Variable Type	Mechanistic study Variable	Outcome Variable	Sub study	Visits
**Eating and Behaviour Computerised Tasks**	Delay Discounting Task	Discounting rate (low, medium, high monetary reward)	fMRI	3,8,10,14
	Leeds Food Choice Task	Sugar bias, fat bias	fMRI	3,8,10,14
	Progressive Ratio Task	Breakpoint	fMRI	3,8,10,14
**Dietary**	Three-Days Food Diary	Total energy intake and Energy intake by macronutrient	fMRI	3,5, 8,10,14
**Food preference tasting tests**	Sweet Taste Detection	EC50 and Corrected hit rate	fMRI	3,5,8
	Consummatory Taste Reward	Just about right, Pleasentess, Intensity	fMRI	3,5,8
**Biological Samples**	Fasting bloods	Glucose	fMRI	3,5, 8,10,14
		Insulin	fMRI	3,5, 8,10,14
		GLP-1	fMRI	3,5, 8,10,14
		PYY	fMRI	3,5, 8,10,14
		Acyl & Desacyl ghrelin	fMRI	3,5, 8,10,14
		Plasma (stored for metabonomics)	fMRI	3,5, 8,10,14
		Bile Acids	fMRI	3,5, 8,10,14
		DNA	fMRI	3,5, 8,10,14
		RNA	fMRI	3,5, 8,10,14
	Post-prandial bloods	Glucose	fMRI	3,5, 8,10
		Insulin	fMRI	3,5, 8,10
		GLP-1	fMRI	3,5, 8,10
		PYY	fMRI	3,5, 8,10
		Acyl & Desacyl ghrelin	fMRI	3,5, 8,10
		Plasma (stored for metabonomics)	fMRI	3,5, 8,10
		Bile Acids	fMRI	3,5, 8,10
	Urine	Urine (stored for metabonomics)	fMRI	3,5, 8,10,14
	Faeces	Faeces (stored for metabonomics)	fMRI	3,5, 8,10,14
		Gut microbiome	fMRI	3,5, 8,10,14
	Biopsies	Stomach for RNA and DNA analysis	fMRI	4, 11
		Duodenum for RNA and DNA analysis	fMRI	4, 11
**Body composition**	Bio-electrical imedance analysis	% body fat	fMRI	3,5, 8,10,14
		Fat & fat free mass	fMRI	3,5, 8,10,14
**MRI**	Resting state fMRI	Connectivity in salience, default mode, auditory, sensorimotor networks	fMRI	3,5
	Arterial spin labelling	Cerebral blood flow	fMRI	3,6
	Food picture evaluation fMRI task	BOLD signal high-energy food > objects, low-energy food > objects, high-energy > low energy food	fMRI	3,7

Mechanistic Variable Type	Mechanistic study Variable	Outcome Variable	Sub study	Visits
	Monetary incentive delay fMRI task	BOLD signal anticipation win > neutral, loss > neutral, win > loss	fMRI	3,8
	Go-NoGo fMRI task	BOLD signal NoGo miss > Go hit, NoGo hit > Go hit	fMRI	3,9
	Negative emotional reactivity fMRI task	BOLD signal unpleasant > neutral pictures	fMRI	3,10
	T1/T2 anatomical	Grey matter density, sub-cortical volumes, brain age, used for image registration	fMRI	3,11
	Diffusion tensor imaging	White matter tract fractional anisotropy, mean diffusivity	fMRI	3,12
		(for all of above applies to both whole brain and region of interest analyses)	fMRI	3,13

Appendix A3: Insulin Clamp Mechanistic Group Variables

Mechanistic Variable Type	Mechanistic study Variable	Outcome Variable	Sub study	Visits
**Questionniares**	Dutch Eating Behaviour Questionnaire (DEBQ)	External eating, dietary restraint, emotional eating	Clamp	3,8,10,14
	Eating Disorder Examination Questionnaire (EDE–Q)	Restraint, eating concern, weight concern, shape concern, global score	Clamp	3,8,10,14
	Behavioural Inhibition and Activation System (BIS / BAS) scales	Drive, reward responsiveness, fun seeking, behavioural inhibition	Clamp	3,8,10,14
	Beck Depression Inventory (BDI–II)	Depression score	Clamp	3,8,10,14
	Barratt Impulsivity Scale and UPPS-P	Negative urgency, premedictation, perserverance, sensation seeking, positive urgency	Clamp	3,8,10,14
	Fagerström Nicotine Dependence Scale	Fagerstroms nicotine dependence score	Clamp	3,8,10,14
	Three Factor Eating Questionnaire (TFEQ)	Disinhibition, dietary restraint, hunger	Clamp	3,8,10,14
	International Physical Activity Questionnaire (IPAQ)	Physical activity	Clamp	3,8,10,14
	Yale Food Addiction Scale	Food addiction	Clamp	3,8,10,14
	Alcohol Use Disorders Identification Test (AUDIT)	Alcohol dependence score	Clamp	3,8,10,14
	Power of Food	Power of food score	Clamp	3,8,10,14
	Pittsburgh Sleep Quality Index (PSQI)	Sleep quality	Clamp	3,8,10,14
	Dumping Syndrome	Arts, Sigstad scores	Clamp	3,8,10,14
	Dumping Syndrome	Arts, Sigstad scores	Clamp	6,7
	Binge Eating Scale	Binge eating score	Clamp	3,8,10,14
	Hospital Anxiety and Depression Scale (HADS)	Anxiety, depression score	Clamp	3,8,10,14
	Short-Form 36 Health Survey Questionnaire (SF36)	Vitality, physical functioning, bodily pain, general health perceptions, physical role functioning, emotional role functioning, social role functioning, mental health	Clamp	3,8,10,14
	Positive and Negative Affect Schedule (PANAS)	Postive & negative affect	Clamp	3,8,10,14
	Barrat Impulsivity Scale	Barratt impulsivity score	Clamp	3,8,10,14
	EPQR	Psychoticism, neuroticism, extraversion, lying	Clamp	3,8,10,14
	Health economics	Resource use, EQ-5D-5L	Clamp	3,8,10,14

Mechanistic Variable Type	Mechanistic study Variable	Outcome Variable	Sub study	Visits
**Visual Analogue Rating Scales**	How hungry do you feel right now (Scale from 1-10)	Hunger	Clamp	3,5,8
	How sick do you feel right now (Scale from 1-10)	Fullness	Clamp	3,5,8
	How pleasant would it be to eat right now (Scale from 1-10)	Nausea	Clamp	3,5,8
	How anxious do you feel right now (Scale from 1-10)	Pleasantness to eat	Clamp	3,5,8
	How much do you think you could eat right now (Scale from 1-10)	Volume to eat	Clamp	3,5,8
	How feel do you feel right now (Scale from 1-10)	Sleepiness	Clamp	3,5,8
	How stressed do you feel right now (Scale from 1-10)	Anxiety	Clamp	3,5,8
	How sleepy do you feel right now (Scale from 1-10)	Stress	Clamp	3,5,8
**Dietary**	Three-Days Food Diary	Total energy intake and Energy intake by macronutrient	Clamp	3,5, 8,10,14
**Biological Samples**	Fasting bloods	Glucose	Clamp	3,5, 8,10,14
		Haematology (FBC)	Clamp	3,5, 8,10,14
		Haematology (HbA1C)	Clamp	5,8,10
		Biochemistry (Renal function, lipid profile, liver function, GGT, AST)	Clamp	3,5, 8,10,14
		Biochemistry (Vitamin D, Vitamin B12, Folate, iron studies, thyroid function)	Clamp	8,10
		Insulin	Clamp	3,5, 8,10,14
		GLP-1	Clamp	3,5, 8,10,14
		PYY	Clamp	3,5, 8,10,14
		Acyl & Desacyl ghrelin	Clamp	3,5, 8,10,14
		Plasma (stored for metabonomics)	Clamp	3,5, 8,10,14
		Bile Acids	Clamp	3,5, 8,10,14
		DNA	Clamp	3,5, 8,10,14
		RNA	Clamp	3,5, 8,10,14
		Fatty acids	Clamp	3,5, 8,10,14
	Urine	Urine (stored for metabonomics)	Clamp	3,5, 8,10,14
		Urinary Albumin/Creatinine ratio	Clamp	3,5, 8,10,14
	Faeces	Faeces (stored for metabonomics)	Clamp	3,5, 8,10,14
		Gut microbiome	Clamp	3,5, 8,10,14
	Biopsies	Stomach for RNA and DNA analysis	Clamp	4, 11, Unsch Exp
		Duodenum for RNA and DNA analysis	Clamp	4, 11, Unsched Exp
	Clamp samples	Glucose for GCMS	Clamp	3,5,8
		Infusates (Dextrose, spiked dextrose)	Clamp	3,5,8
		Insulin infusate	Clamp	3,5,8
		Insulin	Clamp	3,5,8

Mechanistic Variable Type	Mechanistic study Variable	Outcome Variable	Sub study	Visits
		Glucagon	Clamp	3,5,8
		NEFAs	Clamp	3,5,8
		C-Peptide	Clamp	3,5,8
**Body composition**	Bio-electrical imedance analysis	% body fat	Clamp	3,5, 8,10,14
		Total fat mass	Clamp	3,5, 8,10,14

Appendix B – Mixed Model Output

Type 3 Tests of Fixed Effects

Effect	Num DF	Den DF	F Value	Pr > F
Treatment				
Visit				
Covariates				
Interaction Terms				

Solution for Fixed Effects

Effect	Treatment	Visit	Covariates	Estimate	SE	DF	t Value	Pr > |t|
Intercept								
Treatment	…							
	…							
VISIT		…						
		…						
Covariates			…					
Interaction	…	…						

Differences of Least Squares Means / Pairwise Comparisons

Effect	Treat A	Visit A	Treat B	Visit B	Estimate	Standard Error	DF	t Value	Pr > |t|	Adjustment	Adj P
ITMRANDARMASS	Treatment		Control							Tukey-Kramer	
VISITMNEMONIC		V…		V…						Tukey-Kramer	
VISITMNEMONIC		V…		V…						Tukey-Kramer	
VISITMNEMONIC		V…		V…						Tukey-Kramer	
VISITMNEMONIC		V…		V…						Tukey-Kramer	
ITMRANDAR*VISITMNEMO	Treatment	V…	Control	V…						Tukey-Kramer	
ITMRANDAR*VISITMNEMO	Treatment	V…	Control	V…						Tukey-Kramer	
ITMRANDAR*VISITMNEMO	Treatment	V…	Control	V…						Tukey-Kramer	
ITMRANDAR*VISITMNEMO	Treatment	V…	Control	V…						Tukey-Kramer	

Appendix C: Listings

Table 2.1: Listing of protocol deviations

Treatment	Subject	Site	Type	Start Date	End Date

Table 2.3: Listing of protocol violations

Treatment	Subject	Site	Type	Start Date	End Date

Table 5.3.1: Listing of subjects change in medication

Treatment	Subj.	Visit	End Date of Existing Event	Start Date of New Event	Details of New Event
Control					
Treatment					

Table 5.4.3: Listing of All Adverse Events Categorised as Definitely Related to Study Treatment

Subj.	Site	Start Date	End Date	AE Term	Ongoing?	Severity	Action Taken	Serious	Days since Treatment

Table 5.5.1a: Listing of All Serious Adverse Events

Subj.	Details

Table 5.5.1b: Listing of All Serious Adverse Events (continued)

Subj.	Site	Severity	Expected-ness	Days since Treatment	Relative to Treatment	Action Taken	Relative to Device	Device Removed?	SAE Status
