# Supplementary material for: Duodenal-Jejunal Bypass Liner for the management of Type 2 Diabetes Mellitus and Obesity: A Multicenter Randomized Controlled Trial
Source: Ann Surg. 2021 Jun 15;275(3):440–7. doi: 10.1097/SLA.0000000000004980 (PMC8820769; doi:10.1097/SLA.0000000000004980)
Supplement: Supplemental Digital Content [file ansu-275-0440-s003.docx]

**SUPPLEMENTARY INFORMATION**

**Table of Contents**

[**1.** **List of Investigators** 1](#_Toc34038425)

[**2.** **Statistical Considerations** 2](#_Toc34038426)

[**2.1.** **Sample Size calculations** 2](#_Toc34038427)

[**2.2.** **Clarification of Per-Protocol Analysis Set (See Section 5 of SAP)** 2](#_Toc34038428)

[**2.4.** **Post-Hoc Univariate & Multivariate analysis on Baseline Data** 3](#_Toc34038429)

[**2.4.1.** **Methodology** 3](#_Toc34038430)

[**2.4.2.** **Results** 3](#_Toc34038431)

[**2.5.** **Post-Hoc Correlation Testing** 3](#_Toc34038432)

[**2.5.1.** **Methodology** 3](#_Toc34038433)

[**2.5.2.** **Results** 3](#_Toc34038434)

[**3.** **Supplementary Figures** 4](#_Toc34038435)

[4. **Supplementary Tables** 7](#_Toc34038436)

[**5.** **References** 11](#_Toc34038437)

[**6.** **Study Protocol & SAP** 12](#_Toc34038438)

1. **List of Investigators**

Aruchuna Ruban PhD, Alexander D. Miras PhD, Michael A. Glaysher MRCS, Anthony P. Goldstone PhD, Christina G. Prechtl PhD, Nicholas Johnson M.Sc., Navpreet Chhina MRCP, Werd Al-Najim PhD, Madhawi Aldhwayan PhD, Natalia Klimowska-Nassar MSt, Claire Smith PhD, Jia V. Li PhD, Lilliam Flores PhD, Moaz Al-Lababidi MRes, George Dimitriadis MRCP, Mayank Patel DM, Michael Moore FRCGP, Harvinder Chahal PhD, Ahmed R. Ahmed PhD, Jonathan Cousins FRCA, Ghadah Aldubaikhi, Ben Glover MRCP, Emanuela Falaschetti MSc, Hutan Ashrafian PhD, Carel W. le Roux PhD, Ara Darzi PhD, James Byrne MD, Julian P Teare PhD.

1. **Statistical Considerations**
   1. **Sample Size calculations**

The primary end-point of a ≥ 20% reduction in HbA1c was chosen in response to the 2011 International Diabetes Federation guidelines [1] for the conduct of studies in T2DM using obesity/metabolic surgery or devices aiming to produce standardisation and enable comparison between studies 10. It was estimated that 15% of participants in the control arm will achieve the target of 20% reduction. Up to 30% of participants in the treatment group may have the device removed early. We diluted the treatment effect from 40% vs. 15% to 35% vs. 15% achieving the target of 20% reduction in HbA1c for treatment arm vs. standard arm. 73 participants per group would give 80% power to detect a significant effect. Adding 10% loss of follow-up increased the sample size to 80 per group. An additional 5 participants per group were added to account for those who were randomised but withdrew prior to receiving allocated treatment.

- 1. **Clarification of Per-Protocol Analysis Set (See Section 5 of SAP)**

To confirm that early withdrawals/explants in the DJBL arm did not contribute to the trial results, a per-protocol analysis was run across the primary and secondary endpoint variables. The per-protocol population consists of those who adhered to study treatment. In the DJBL arm this consists of all patients who had the Endobarrier implanted for the entire 12-month treatment period. In the Control arm, this consists of all patients who attended the dietician reviews at M3, M6 and M9.

- 1. **Missing Data Analysis on the Primary Outcome (See Section 7.6 of SAP)**
     1. **Methodology**

Under the assumption that data is missing-at-random, multiple imputation by chained equations (MICE) was used to investigate the effect of missing data on the primary analysis. The values of the unobserved data was assumed to be conditional on treatment group and stratification factors (BMI groups and sites) as well as on HbA1c values at timepoints M3, M6, M9 and M12. A total of 50 imputed data sets were drawn separately for each randomised group, replacing missing outcome values with simulated values from a set of imputation models containing BMI group, sites, HbA1c values at M3, M6, M9 and M12. Using MICE, missing values for the binary outcome were imputed using a binary logistic model, including all other covariates. Missing values for any of the continuous interim HbA1c values included in the imputation model was also imputed using linear regression models.

Parameter estimates across the 50 iterations was combined using Rubin’s rules.

Alongside MICE, we also examined the difference in proportion of substantial improvement amongst those missing and those observed to obtain an alternative result from that concluded from the complete case analysis difference between the two arms. Four scenarios have been considered:

1. Missing participants within the EndoBarrier arm to have an increased rate of substantial improvement. Missing participants within the standardised treatment arm to have the same rate of substantial improvement.

2. Missing participants within the standardised treatment arm to have an increased rate of substantial improvement. Missing participants within the EndoBarrier arm to have the same rate of substantial improvement.

3. Missing participants within the EndoBarrier arm to have a lower rate of substantial improvement. Missing participants within the standardised treatment arm to have the same rate of substantial improvement.

4. Missing participants within the standardised treatment arm to have a lower rate of substantial improvement. Missing participants within the EndoBarrier arm to have the same rate of substantial improvement.

The value of the proportion required within the missing data to affect the result for each scenario was reported.

- - 1. **Results**

Using MICE, when combining the parameter estimates of all 50 iterations, the estimated treatment effect (in terms of proportion of patients achieving 20% reduction in HbA1c) was 0.025 (95% CI: -0.34, 0.39; p=.89).

Under the ITT analysis at 12 months 30 of 55 participants (54.5%) achieved a 20% reduction in HbA1c in the DJBL group, compared to 32 of 58 (55.2%) in the control group (p=.85). When fixing the success rate to 55.2% in the control group, in order to get a significant result where DJBL is superior all 30 missing patients in the DJBL arm (100%) are required to achieve a 20% reduction in HbA1c. To obtain a significant result where the control arm is superior, a maximum of 4 (13.3%) patients in the DJBL arm can achieve the required 20% reduction. Fixing the success rate to 54.5% in the DJBL group requires all 27 missing patients in the control arm (100%) to achieve a 20% reduction in HbA1c in order to get a significant result where the control group is superior. To obtain a significant result where the DJBL arm is superior, a maximum of only 1 (3.7%) patient in the control arm can achieve the 20% reduction in HbA1c. For each of the above four scenarios, an event rate significantly different from that found in the current population would be required.

- 1. **Post-Hoc Univariate & Multivariate analysis on Baseline Data**
     1. **Methodology**

In both DJBL and control groups, just over half of patients reported a 20% reduction in HbA1c. One question that arose from this outcome was whether there were any identifiers that allowed us to predict whether a patient receiving DJBL or strict dietary advice would be successful in reducing their HbA1c levels after 1 year. To investigate this, a univariate analysis was carried out separately within both arms. Looking only at patients in the per-protocol population who adhered to treatment (did not have early explant in DJBL group or attended all dietician appointments in control group) we tested (via one-way ANOVA) percentage HbA1c change at 12 months against; baseline variables, primary and secondary endpoint variables LFTs and lipids. Test results with a p-value of less than 0.1 were subsequently recorded and used together in a multivariate model to establish any potential baseline markers for endpoint adherence. The multivariate model was analysed using linear regression when investigating HbA1c as percentage change at 1 year and logistic regression when investigating HbA1c as those achieving 20% reduction at 1 year.

A similar method was introduced incorporating the data from both treatment groups. Each baseline variable was individually tested within an ANCOVA including terms for treatment and the corresponding interaction. A variable was selected for the corresponding ‘multivariate’ approach when either the variable term, the treatment term or the interaction term was less than 0.1. The selected variables and their corresponding interaction terms with treatment were included in a stepwise regression model investigating primary endpoint achievement alongside additional terms for treatment, site and BMI subgroup.

- - 1. **Results**

In the DJBL group, univariate analysis selected 4 variables to be included in a multivariate model; sex (p=.08), log_BMI (p=.01), log_AST(p=.08) and log_ALT (p=.002). The corresponding multivariate model when used to investigate a 20% reduction in HbA1c levels at 1 year established a potential relation between log_ALT (p=.03) and a positive outcome.

In the control group, univariate analysis selected 6 variables to be included in a multivariate model; log_HbA1c (p=.06), log_triglycerides (p=.09), log_LDL (p=.07), log_AST (p=.04), log_ALP (p=.08) and log_ALT (p=.007). The corresponding multivariate model when used to investigate a 20% reduction in HbA1c levels at 1 year did not report any significant variables with the closest being log_HbA1c p=.05).

The results do not conclusively suggest that a baseline (or combination of) variable(s) can accurately predict whether a patient is likely to achieve a 20% reduction in HbA1c after one year of treatment, whether via using DJBL or via dietary assistance.

- 1. **Post-Hoc Correlation Testing**
     1. **Methodology**

In a similar vein to the above testing. A correlation test was carried out using PROC CORR (SAS v9.4) between change in HbA1c and weight levels at month 3 to see how they correlate to the change at month 12 (end of treatment). Additional correlation testing was carried out between change in HbA1c and weight at 12 months against 12-month change in vitals output, liver function tests output and fasting lipids levels. Pearson correlation-coefficients (PCC’s) of over |0.4| have been reported.

- - 1. **Results**

Weight loss at 3 months was highly correlated with weight loss at 12 months with correlation coefficients of 0.82 (DJBL) and 0.86 (control). HbA1c reduction at 3 months was moderately correlated with the 12-month response with correlation coefficients of 0.56 (DJBL) and 0.62 (control). There was no correlation between weight loss and improvements in glycaemic, lipid or blood pressure parameters.

1. **Supplementary Figures**


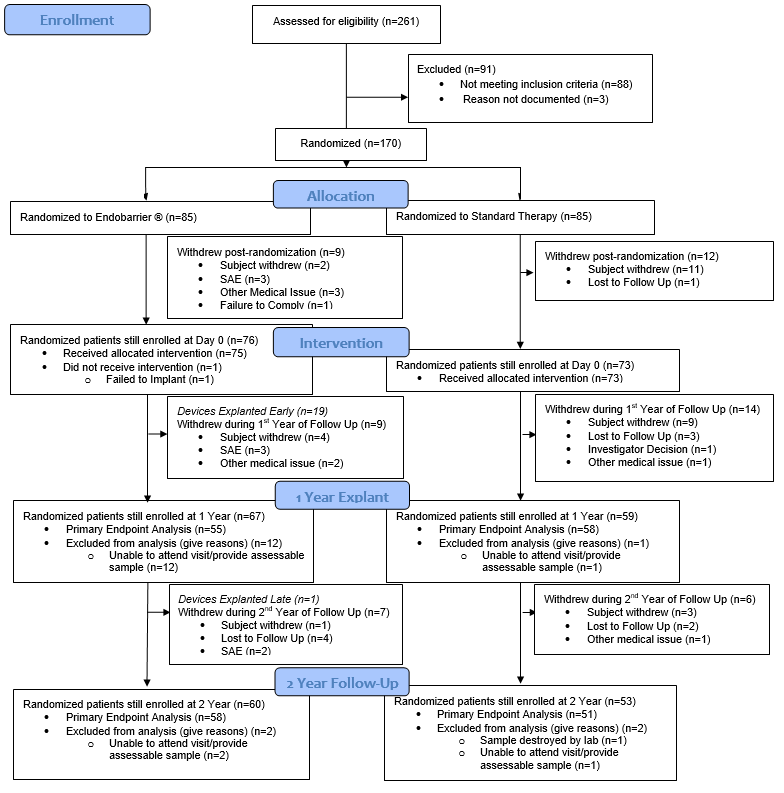


SAE = Serious Adverse Event

**eFigure1: CONSORT flowchart of patient recruitment and retention**

**eFigure2: HbA1c Per-Protocol Output**

Figure showing plot of mean ± SD HbA1c levels per visit. DJBL = Duodenal jejunal bypass liner, SD = Standard Deviation


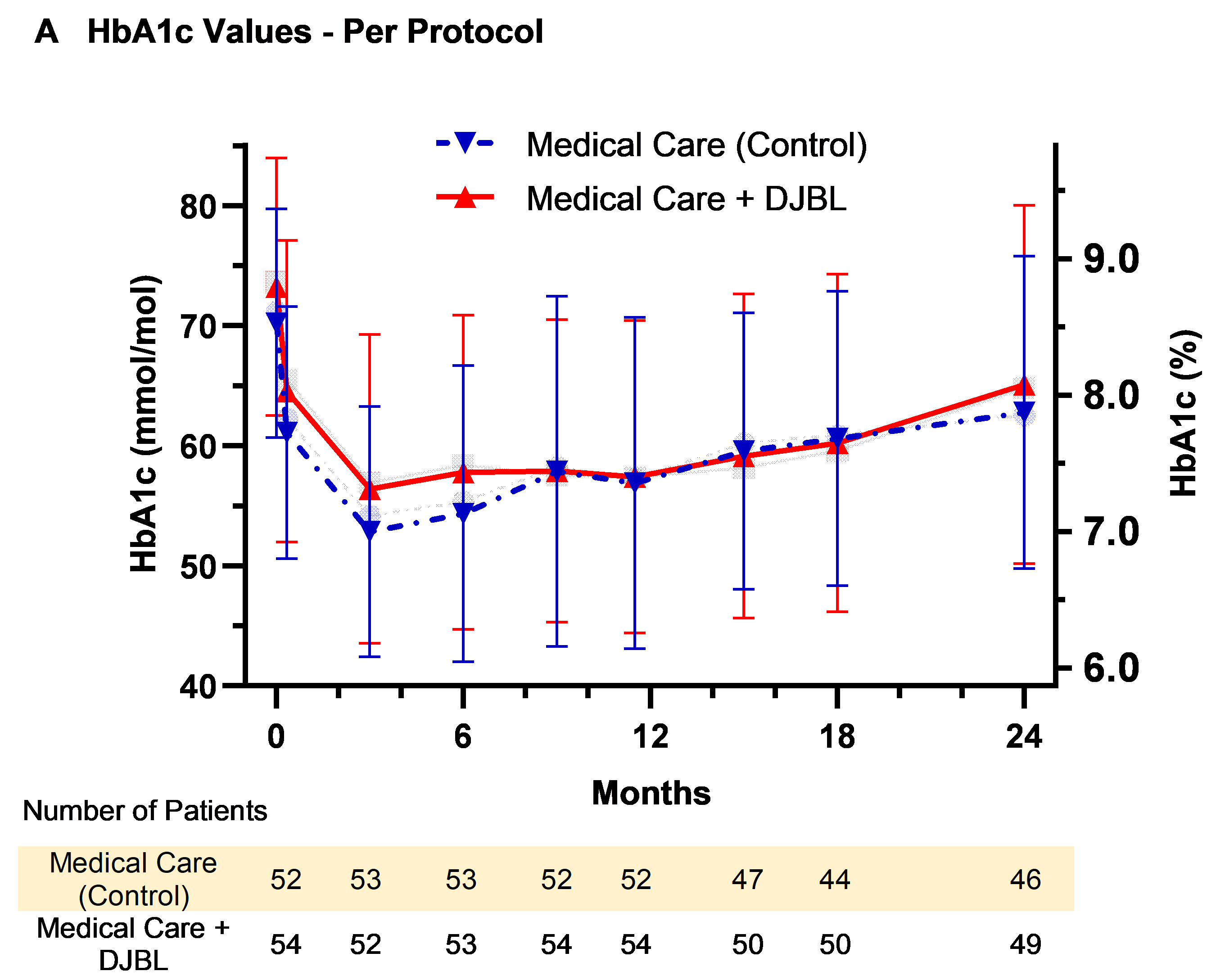

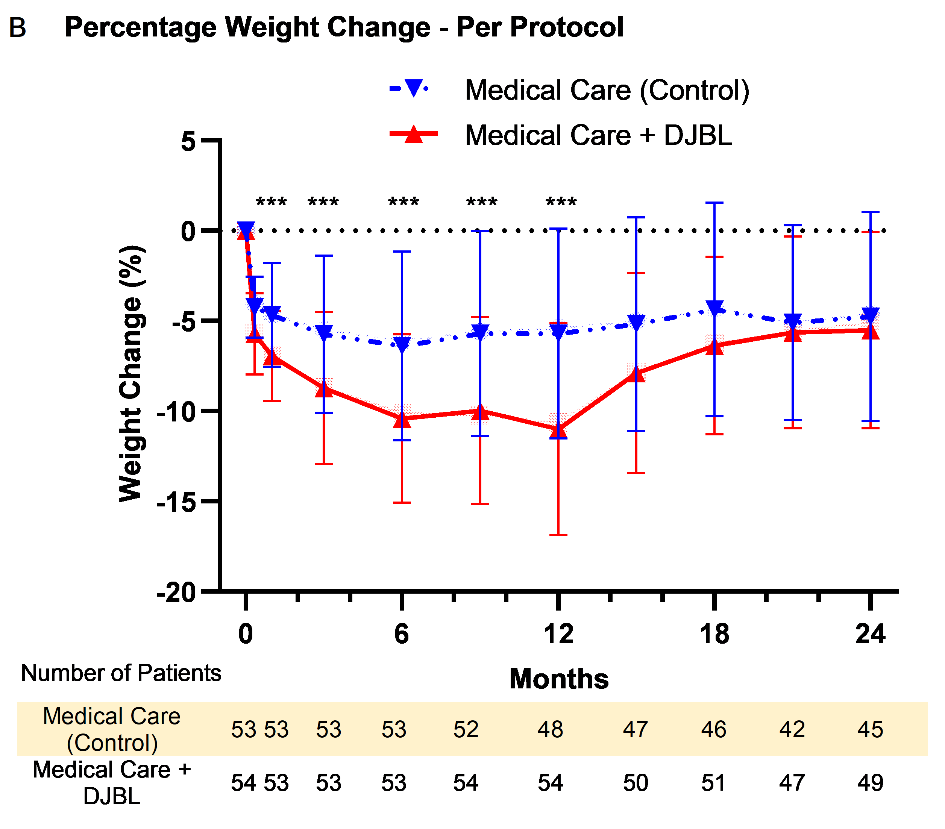


**eFigure3: Frequency distribution of weight-loss**

The above figure highlights the significant difference in weight-loss distributions between treatment groups at M12. By M24 the DJBL distribution curve has shifted back, almost overlaying that in the control medical care group. DJBL = Duodenal Jejunal Bypass Liner. SD = Standard Deviation.


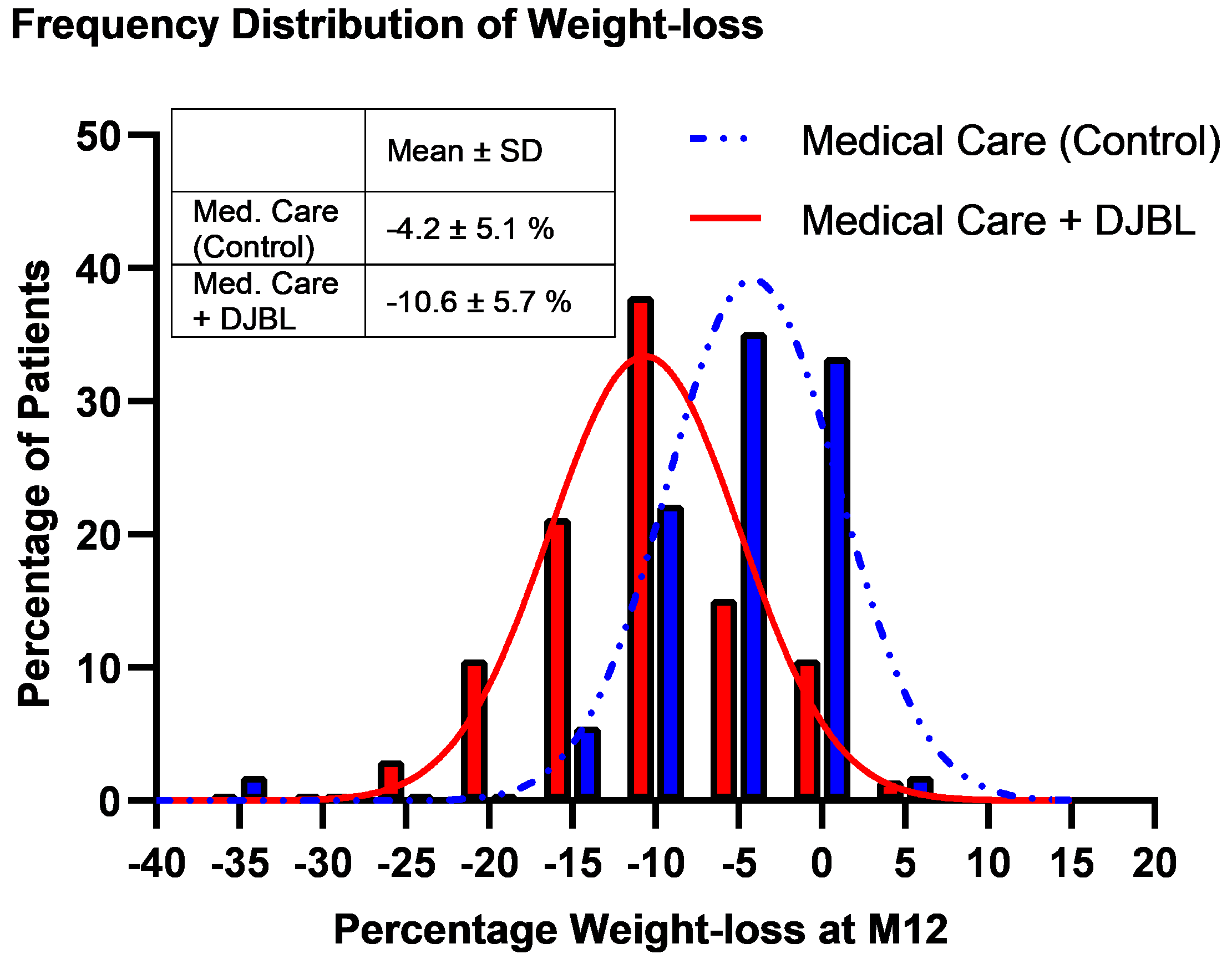

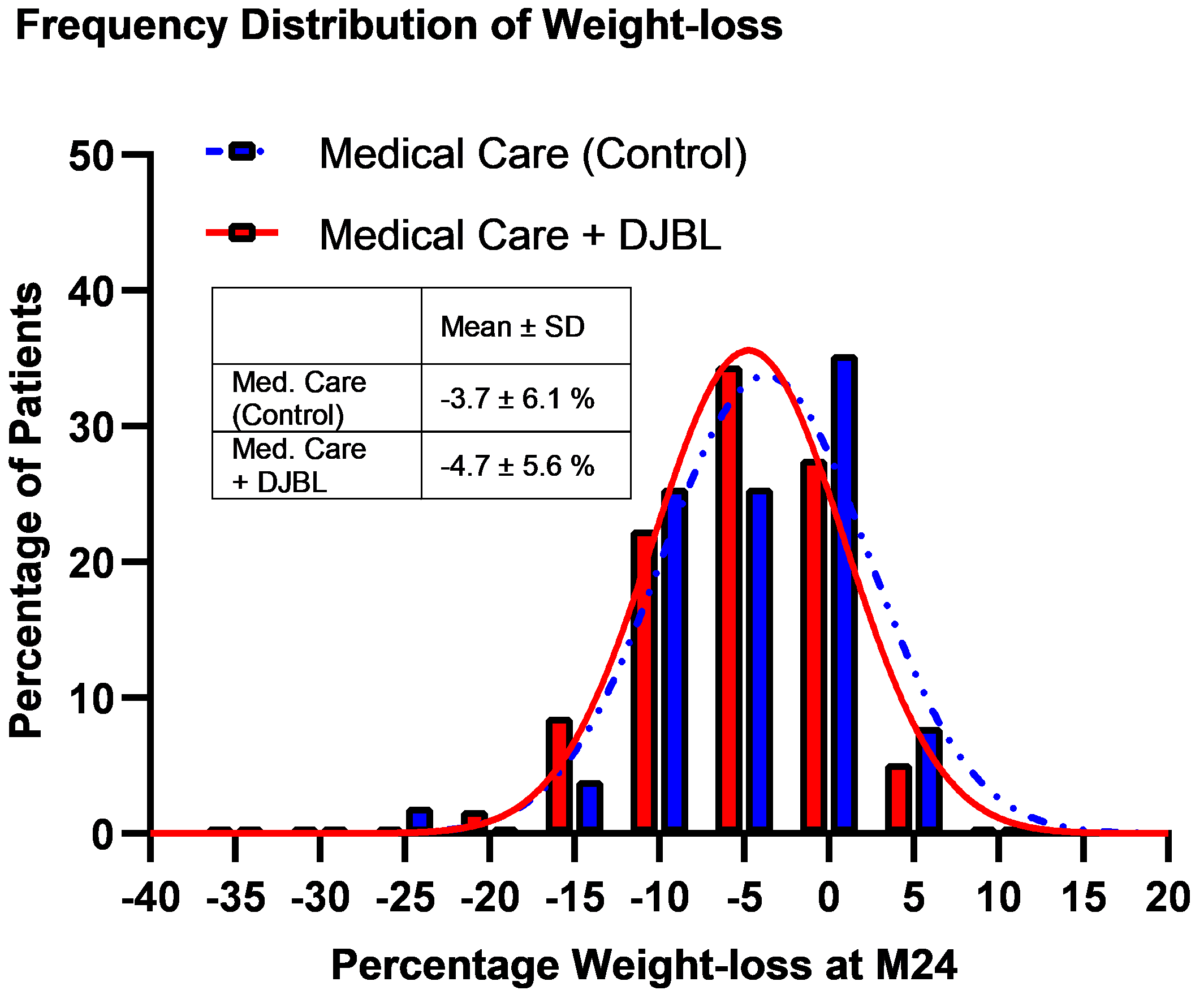


1. **Supplementary Tables**

| eTable1. Adverse Events by Class & Term ^a^ | | | |
| --- | --- | --- | --- |
| Class | **Term** | **DJBL ^b^**  **(N=85)** | **Control**  **(N=85)** |
| Gastrointestinal Disorders | Abdominal Pain | 81 | 9 |
|  | Diarrhoea | 30 | 27 |
|  | Nausea | 35 | 4 |
|  | Vomiting | 36 | 3 |
|  | Constipation | 19 | 12 |
|  | Abdominal Discomfort | 22 | . |
|  | Abdominal Distension | 8 | 3 |
|  | Gastroenteritis | 5 | 5 |
|  | Gastro-oesphageal Reflux Disease | 7 | 2 |
|  | Abdominal Cramps | 5 | 2 |
|  | Loose Stool | 3 | 2 |
|  | Dyspepsia | 3 | 1 |
|  | Belching | 1 | 1 |
|  | Flatulence | 1 | 1 |
|  | Indigestion | 2 | . |
|  | Rectal Haemorrhage | 1 | 1 |
|  | Abdominal Bloating | 1 | . |
|  | Anal Fissure | 1 | . |
|  | Change In Bowel Habit | . | 1 |
|  | Colonic Polyps | 1 | . |
|  | Diverticular Disease | 1 | . |
|  | Dumping Syndrome | 1 | . |
|  | Duodenitis | 1 | . |
|  | Dysphagia | . | 1 |
|  | Eructation | 1 | . |
|  | Frequent Urination | 1 | . |
|  | Gastritis | 1 | . |
|  | Haemorrhoids | 1 | . |
|  | Kidney Stones | 1 | . |
|  | Tooth Ache | 1 | . |
|  | Tooth Pain | . | 1 |
| Musculoskeletal And Connective Tissue Disorders | Back Pain | 18 | 13 |
|  | Pain In Extremity | 5 | 12 |
|  | Musculoskeletal Pain | 2 | 14 |
|  | Joint Swelling | 2 | 5 |
|  | Arthralgia | 2 | 3 |
|  | Athralgia | 3 | 1 |
|  | Carpal Tunnel Syndrome | 3 | . |
|  | Elbow Pain | 2 | . |
|  | Lower Back Pain | . | 2 |
|  | Neck Pain | 1 | 1 |
|  | Abscess Neck | 1 | . |
|  | Ankle Fracture | . | 1 |
|  | Arthritis Bacterial | 1 | . |
|  | Bursitis | . | 1 |
|  | Clavicle Fracture | 1 | . |
|  | Elbow Fracture | 1 | . |
|  | Leg Pain | 1 | . |
|  | Ligament Rupture | . | 1 |
|  | Ligament Sprain | 1 | . |
|  | Musculoskeletal Chest Pain | 1 | . |
|  | Myalgia | 1 | . |
|  | Nerve Impingement | . | 1 |
|  | Osteoarthritis | . | 1 |
|  | Periarthritis | . | 1 |
|  | Rib Fracture | 1 | . |
|  | Shoulder Pain | 1 | . |
|  | Tendinosis | . | 1 |
|  | Tendonitis | 1 | . |
|  | Thumb Fracture | . | 1 |
|  | Wrist Sprain | . | 1 |
| Infections And Infestations | Urinary Tract Infection | 9 | 5 |
|  | Pneumonia | 2 | 7 |
|  | Candida Infection | 5 | 2 |
|  | Helicobacter Infection | 4 | 2 |
|  | Tooth Infection | 3 | 1 |
|  | Infected Bite | . | 3 |
|  | Oral Candidiasis | 3 | . |
|  | Sinusitis | . | 3 |
|  | Viral Infection | 1 | 2 |
|  | Cellulitis | . | 2 |
|  | Breast Abscess | 1 | . |
|  | Conjunctivitis | . | 1 |
|  | Ear Infection | 1 | . |
|  | Finger Infection | . | 1 |
|  | Gum Disease | 1 | . |
|  | Peritonsillar Abscess | . | 1 |
|  | Pollakiuria | . | 1 |
|  | Sepsis | 1 | . |
|  | Toe Infection | . | 1 |
|  | Vulvovaginal Candidiasis | 1 | . |
| Respiratory, Thoracic And Mediastinal Disorders | Influenza | 3 | 17 |
|  | Lower Respiratory Tract Infection | 7 | 13 |
|  | Cough | 7 | 4 |
|  | Nasopharyngitis | 5 | 6 |
|  | Upper Respiratory Tract Infection | 2 | 4 |
|  | Dyspnoea | 2 | . |
|  | Oropharyngeal Pain | 2 | . |
|  | Seasonal Allergy | . | 2 |
|  | Allergy | 1 | . |
|  | Asthma | 1 | . |
|  | Bronchitis | . | 1 |
|  | Rhinorrhoea | . | 1 |
| Nervous System Disorders | Dizziness | 6 | 8 |
|  | Headache | 5 | 6 |
|  | Epileptic Seizure | 4 | . |
|  | Sciatica | 3 | 1 |
|  | Paraesthesia | 2 | . |
|  | Burning Sensation | . | 1 |
|  | Charcot's Disease | 1 | . |
|  | Facial Paralysis | . | 1 |
|  | High-Grade Glioma | . | 1 |
|  | Insomnia | 1 | . |
|  | Presyncope | . | 1 |
|  | Trigeminal Neuralgia | . | 1 |
| Endocrine Disorders | Hypoglycaemia | 9 | 20 |
|  | Goitre | . | 1 |
|  | Hyperglycaemia | 1 | . |
|  | Thyroxine Overreplacement | . | 1 |
| Surgical And Medical Procedures | Dental Operation | 1 | 3 |
|  | Tooth Extraction | . | 2 |
|  | Cardioversion | 1 | . |
|  | Cyst Excision | . | 1 |
|  | Hernia Repair | . | 1 |
|  | Knee Arthroscopy And Debridement | . | 1 |
|  | Mole Excision | . | 1 |
|  | Plastic Surgery | . | 1 |
|  | Polypectomy | . | 1 |
|  | Renal Stone Removal | 1 | . |
|  | Renal Surgery | 1 | . |
|  | Retinal Laser Coagulation | . | 1 |
|  | Rotator Cuff Repair | . | 1 |
|  | Shoulder Operation | 1 | . |
|  | Skin Biopsy | 1 | . |
|  | Skin Cyst Excision | . | 1 |
|  | Skin Lesion Removal | . | 1 |
|  | Spinal Decompression | 1 | . |
|  | Thyoidectomy | . | 1 |
|  | Tooth Filling | . | 1 |
|  | Vaginal Hysterectomy | 1 | . |
|  | Vitrectomy | 1 | . |
| Metabolism And Nutrition Disorders | Vitamin D Deficiency | 3 | 2 |
|  | Vitamin B12 Deficiency | 2 | 2 |
|  | Gout | 3 | . |
|  | Hyperglycaemia | 2 | . |
|  | Dehydration | 2 | . |
|  | Iron Deficiency | 2 | . |
|  | Hepatic Steatosis | . | 1 |
|  | Decreased Appetite | 1 | . |
|  | Ft4 Elevated | . | 1 |
|  | Hypercholesterolaemia | 1 | . |
|  | Hypokalaemia | 1 | . |
|  | Hyponatraemia | . | 1 |
|  | Ketosis | 1 | . |
| Investigations | Liver Function Test Abnormal | 6 | 3 |
|  | Gamma-Glutamyltransferase Increased | . | 2 |
|  | Nuclear Magnetic Resonance Imaging Abnormal | 1 | 1 |
|  | Abnormal Liver Function Tests | 1 | . |
|  | Alanine Aminotransferase Increased | . | 1 |
|  | Arthroscopy | . | 1 |
|  | Breast Biopsy | . | 1 |
|  | Computerised Tomogram | . | 1 |
|  | Endoscopy Nos | 1 | . |
|  | Laparoscopy | 1 | . |
|  | Liver Function Test Increased | . | 1 |
|  | White Blood Cell Count Increased | 1 | . |
| General Disorders And Administration Site Conditions | Fatigue | 3 | 2 |
|  | Surgical Failure | 5 | . |
|  | Chest Pain | 2 | 1 |
|  | Peripheral Swelling | 1 | 1 |
|  | Balanitis | 1 | . |
|  | Hot Flush | . | 1 |
|  | Malaise | . | 1 |
|  | Menopausal Symptoms | 1 | . |
|  | Oedema | . | 1 |
| Injury, Poisoning And Procedural Complications | Fall | 5 | . |
|  | Back Injury | . | 4 |
|  | Limb Injury | . | 4 |
|  | Accidental Overdose | 1 | . |
|  | Arthropod Bite | . | 1 |
|  | Head Injury | . | 1 |
|  | Muscle Sprain | . | 1 |
|  | Road Traffic Accident | . | 1 |
|  | Skin Abrasion | 1 | . |
|  | Whiplash Injury | . | 1 |
| Skin And Subcutaneous Tissue Disorders | Cellulitis | 1 | 2 |
|  | Skin Ulcer | 1 | 2 |
|  | Rash | 2 | . |
|  | Adiposis Dolorosa | . | 1 |
|  | Fungal Skin Infection | . | 1 |
|  | Impetigo | 1 | . |
|  | Itching | 1 | . |
|  | Lipoma | 1 | . |
|  | Nail Infection | . | 1 |
|  | Sacral Cyst Abscess | 1 | . |
|  | Shingles | . | 1 |
|  | Skin Disorder | 1 | . |
|  | Skin Lesion | 1 | . |
|  | Urticaria | 1 | . |
| Product Issues | Device Malfunction | 10 | . |
|  | Device Migration | 7 | . |
| Blood And Lymphatic System Disorders | Anaemia | 5 | 1 |
|  | Microcytic Anaemia | 4 | . |
|  | Normocytic Anaemia | . | 2 |
|  | Hypoalbuminaemia | 1 | . |
| Eye Disorders | Retinopathy | 2 | 1 |
|  | Vitreous Haemorrhage | 2 | . |
|  | Blepharitis | . | 1 |
|  | Blurred Vision | 1 | . |
|  | Corneal Ulcer | . | 1 |
|  | Diabetic Retinopathy | . | 1 |
|  | Dry Eye | 1 | . |
|  | Eye Infection | 1 | . |
|  | Fluid Build-Up | . | 1 |
|  | Glaucoma | . | 1 |
| Renal And Urinary Disorders | Haematuria | 2 | . |
|  | Microalbuminuria | 1 | 1 |
|  | Pollakiuria | . | 1 |
|  | Micturition Urgency | . | 1 |
|  | Nephrolithiasis | 1 | . |
|  | Pyelonephritis | 1 | . |
|  | Renal Colic | 1 | . |
|  | Ureterolithiasis | 1 | . |
|  | Urinary Incontinence | 1 | . |
| Vascular Disorders | Bradycardia | . | 2 |
|  | Upper Gastrointestinal Haemorrhage | 2 | . |
|  | Rectal Haemorrhage | . | 1 |
|  | Hot Flush | 1 | . |
|  | Cerebellar Infarction | 1 | . |
|  | Varicose Vein Operation | . | 1 |
| Psychiatric Disorders | Anxiety | 3 | . |
|  | Depression | 1 | 2 |
|  | Panic Attack | 1 | . |
| Ear And Labyrinth Disorders | Vertigo Positional | 2 | 2 |
|  | Otitis Media | . | 1 |
|  | Vertigo | . | 1 |
| Cardiac Disorders | Acute Coronary Syndrome | 1 | . |
|  | Atrial Fibrillation | 1 | . |
|  | Palpitations | 1 | . |
|  | Stroke | . | 1 |
|  | Ventricular Fibrillation | 1 | . |
| Hepatobiliary Disorders | Hepatic Steatosis | 1 | 1 |
|  | Cholecystitis Acute | 1 | . |
|  | Liver Abscess | 1 | . |
| Immune System Disorders | Crohns Disease | 1 | . |
|  | Psoriatic Arthropathy | 1 | . |
|  | Sarcoidosis | . | 1 |
| Reproductive System And Breast Disorders | Pv Bleed | . | 2 |
|  | Balanitis Candida | 1 | . |
| Pregnancy, Puerperium And Perinatal Conditions | Breast Abscess | 1 | 1 |
| a Figures are per patient. In the event where a reported AE contains more than one affliction both classes/terms have been counted. | | | |
| b DJBL = Duodenal Jejunal Bypass Liner | | | |

1. **References**

1. Dixon, J.B., et al., *Bariatric surgery: an IDF statement for obese Type 2 diabetes.* Diabet Med, 2011. **28**(6): p. 628-42.

1. **Study Protocol & SAP**

Protocol Version 5.0, 5^th^ March 2018

SAP Version 1.0, 21th February 2019
